# Pharmacokinetics, safety, and efficacy of Fuqi Guben Gao in the treatment of kidney-yang deficiency syndrome: a randomized, double-blind phase I trial

**DOI:** 10.3389/fphar.2024.1351871

**Published:** 2024-06-25

**Authors:** Wei-Yi Cao, Jun-Yu Liu, Min Sun, Jing-Kun Wang, Fang Lu, Qiao-Ning Yang, Wan-Tong Zhang, Ming-Jie Zi, Bai-E Zhang, Hong-Bin Liu, Shu-Ge Wang, Yi Wu, Rong-Zu Wu, Wen-Di Wu, Rui Li, Zhao-Yun Zhu, Rui Gao

**Affiliations:** ^1^ Institute of Clinical Pharmacology of Xiyuan Hospital, National Clinical Research Center for Chinese Medicine Cardiology, China Academy of Chinese Medical Sciences, Beijing, China; ^2^ NMPA Key Laboratory for Clinical Research and Evaluation of Traditional Chinese Medicine, National Clinical Research Center for Chinese Medicine Cardiology, Beijing, China; ^3^ Yunnan Province Company Key Laboratory for TCM and Ethnic Drug of New Drug Creation, Yunnan Institute of Materia Medica, Kunming, Yunnan, China; ^4^ Yunnan Baiyao Group Co., Ltd., Kunming, Yunnan, China; ^5^ Kunming Municipal Hospital of Traditional Chinese Medicine, Kunming, Yunnan, China

**Keywords:** Fuqi Guben Gao, pharmacokinetics, metabonomics, efficacy, kidney-yang deficiency syndrome

## Abstract

**Introduction:** Fuqi Guben Gao (FQGBG) is a botanical drug formulation composed of FuZi (FZ; *Aconitum carmichaelii* Debeaux [Ranunculaceae; Aconiti radix cocta]), Wolfberry (*Lycium barbarum* L. [Solanaceae; Lycii fructus]), and Cinnamon (*Neolitsea cassia* (L.) Kosterm. [Lauraceae; Cinnamomi cortex]). It has been used to clinically treat nocturia caused by kidney-yang deficiency syndrome (KYDS) for over 30 years and warms kidney yang. However, the pharmacological mechanism and the safety of FQGBG in humans require further exploration and evaluation.

**Methods:** We investigated the efficacy of FQGBG in reducing urination and improving immune organ damage in two kinds of KYDS model rats (hydrocortisone-induced model and natural aging model), and evaluated the safety of different oral FQGBG doses through pharmacokinetic (PK) parameters, metabonomics, and occurrence of adverse reactions in healthy Chinese participants in a randomized, double-blind, placebo-controlled, single ascending dose clinical trial. Forty-two participants were allocated to six cohorts with FQGBG doses of 12.5, 25, 50, 75, 100, and 125 g. The PKs of FQGBG in plasma were determined using a fully validated LC-MS/MS method.

**Results:** FQGBG significantly and rapidly improved the symptoms of increased urination in both two KYDS model rats and significantly resisted the adrenal atrophy in hydrocortisone-induced KYDS model rats. No apparent increase in adverse events was observed with dose escalation. Major adverse drug reactions included toothache, thirst, heat sensation, gum pain, diarrhea, abdominal distension, T-wave changes, and elevated creatinine levels. The PK results showed a higher exposure level of benzoylhypaconine (BHA) than benzoylmesaconine (BMA) and a shorter half-life of BMA than BHA. Toxic diester alkaloids, aconitine, mesaconitine, and hypaconitine were below the lower quantitative limit. Drug-induced metabolite markers primarily included lysophosphatidylcholines, fatty acids, phenylalanine, and arginine metabolites; no safety-related metabolite changes were observed.

**Conclusion:** Under the investigated dosing regimen, FQGBG was safe. The efficacy mechanism of FQGBG in treating nocturia caused by KYDS may be related to the improvement of the hypothalamus-pituitary-adrenal axis function and increased energy metabolism.

**Clinical Trial Registration:** https://www.chictr.org.cn/showproj.html?proj=26934, identifier ChiCTR1800015840.

## 1 Introduction

Nocturia, frequent night-time urination, affects up to 60% of people over 65, increasing risks of falls, fractures, depression, and death (Bliwise et al., 2019; Lombardo et al., 2020). Aging and diseases, such as changes in the central nervous system and reduced renal function, are primary causes (Wolff et al., 2021). Traditional Chinese Medicine (TCM) attributes nocturia to kidney-yang deficiency (KYDS), where the kidney, responsible for storing the essence of yin and yang, fails to control water. Warming kidney yang is a common TCM treatment for this condition (Lu et al., 2009; Xu et al., 2019). Fuqi Guben Gao (FQGBG), a botanical drug formulation to treat nocturia caused by KYDS, is derived from the clinical experience in Yunnan Province, China. It is composed of FuZi (FZ; *Aconitum carmichaelii* Debeaux [Ranunculaceae; Aconiti radix cocta]), Wolfberry (*Lycium barbarum* L. [Solanaceae; Lycii fructus]), and Cinnamon (*Neolitsea cassia* (L.) Kosterm. [Lauraceae; Cinnamomi cortex]). In 2013, Kunming Hospital of TCM conducted a randomized double-blind clinical study to evaluate the efficacy and safety of FQGBG in 72 patients with KYDS. The results showed that the effective rate in the treatment group was 95% (Sun et al., 2017). The FZ-Cinnamon pair is commonly used in the clinical treatment of KYDS and has been reported to improve the adrenal cortex function and physical fitness of hydrocortisone-induced KYDS in mice (Qian, 2019). Preclinical pharmacological study results indicated that FQGBG substantially improved polyuria in four kinds of KYDS rat models, including the excessive fatigue, adenine-induced, hydrocortisone-induced, and natural aging types. In addition, the effect observed was faster than positive controls (Gui-fu-di-huang-wan and You-gui-wan). Moreover, FQGBG can significantly counteract hydrocortisone-induced adrenal atrophy in rats with KYDS, increase their adrenocorticotropic hormone response, and significantly prolong the ice-water swimming time of mice with KYDS. Based on the above findings, FQGBG was developed by the Yunnan Institute of Materia Medica and Yunnan Baiyao Group Co., Ltd., and has been approved for clinical trials by the National Medical Products Administration (NMPA, No. 2016L10230) as an investigational agent in Chinese medicine.

**FIGURE 1:**
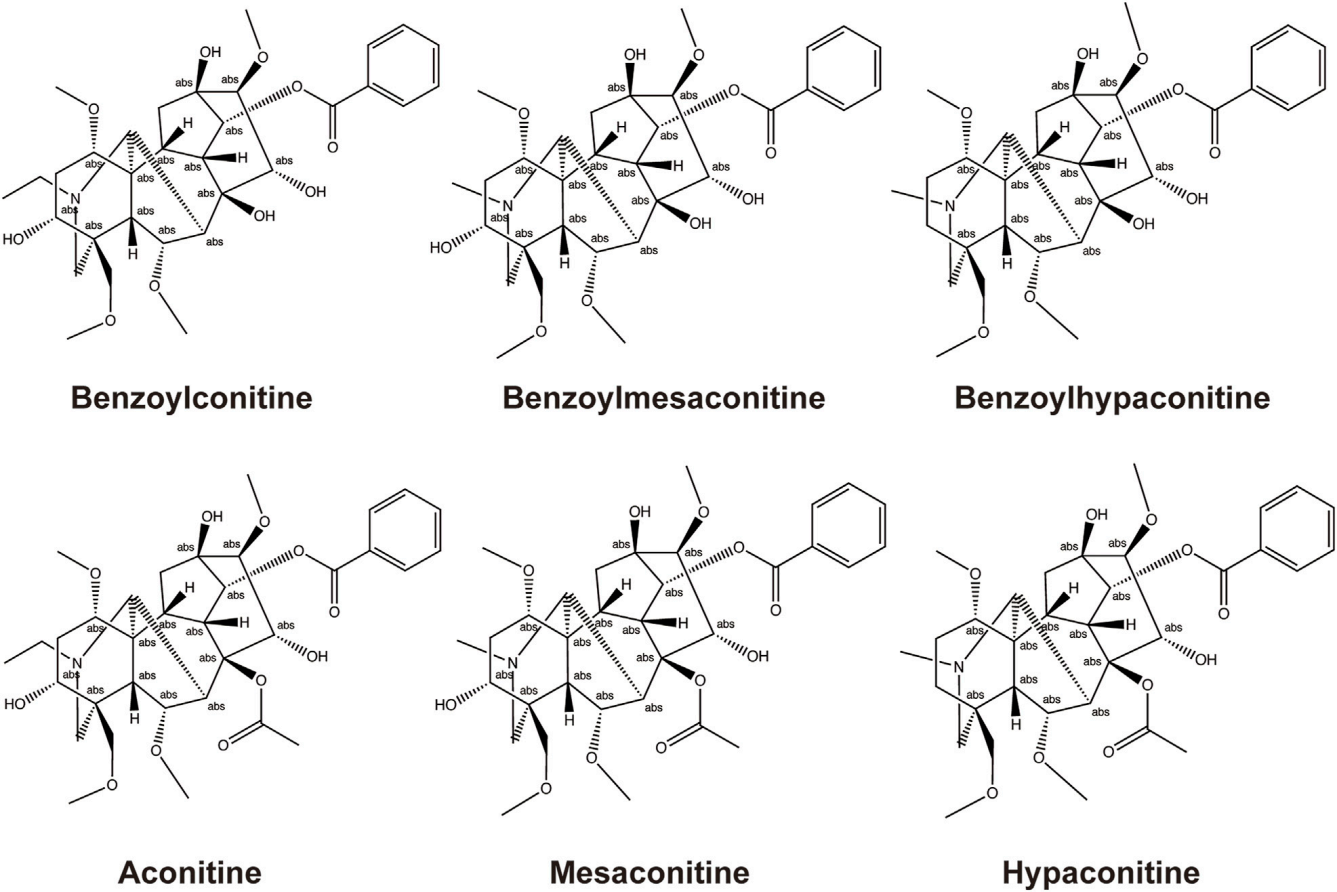
Chemical structural formulas of the major diterpene alkaloids in FQGBG.

The main active metabolites of FZ include three highly toxic diester–diterpene alkaloids: aconitine (AC), mesaconitine (MA), and hypaconitine (HA). According to a previous report, the narrow therapeutic window of AC limits the clinical application of AC-containing botanical drugs; overdosing on AC always induces ventricular tachyarrhythmia and heart arrest. However, emerging evidence shows that low doses of AC or its metabolites could generate cardioprotective effects and are necessary to the clinical efficacy of aconites (Zhou et al., 2021). Thus, owing to this toxicity, FZ must be adequately processed by hydrolyzing AC, MA, and HA into less toxic monoester-diterpene alkaloids, benzoylaconine (BAC), benzoylmesaconine (BMA), and BHA (Singhuber et al., 2009), and its drug safety must be closely monitored. The structural formula of BAC, BMA, BHA, AC, MA, and HA is shown in Figure 1.

Pharmacokinetic (PK) studies help explain and predict various events related to drug efficacy and toxicity. Thus, it is valuable to determine PK parameters in evaluating the rationality and safety of clinical prescriptions (Yan et al., 2018). Metabonomics is a high-throughput detection method for endogenous small molecules and provides information on the entire organism’s functional integrity over time following exposure to a perturbation (Lu et al., 2011). It is widely used in mining pathological, pharmacological, and drug safety biomarkers and in establishing disease diagnosis models. Until now, the potential toxic mechanism of Aconitum alkaloids has been studied through metabonomics (Ji et al., 2023).

In this study, we investigated the efficacy of FQGBG in improving the symptoms of kidney-yang deficiency in two types of KYDS model rats, and evaluated the safety of different oral doses of FQGBG through PK parameters, metabonomics and occurrence of adverse reactions in healthy Chinese participants. This study attempts to implement an evaluation model that combines drug exposure, endogenous metabolites, and clinical manifestations, and will provide evidence for the mechanism of exploration of the safety and efficacy of FQGBG in humans.

## 2 Methods

### 2.1 Materials

FQGBG comprises FuZi (FZ *Aconitum carmichaelii* Debeaux [Ranunculaceae; Aconiti radix cocta]), Wolfberry (*Lycium barbarum* L. [Solanaceae; Lycii fructus]), and Cinnamon (*Neolitsea cassia* (L.) Kosterm. [Lauraceae; Cinnamomi cortex]). The plants’ names were verified at http://mpns.kew.org/mpns-portal/. All pharmaceutical raw materials were processed and quality tested according to the Pharmacopoeia of China (2015). The processing method for FZ involves taking the daughter roots of the Ranunculaceae family plant *Aconitum carmichaelii* Debeaux, immersing them in a bile salts aqueous solution for several days, and then boiling them with the soaking solution until they are thoroughly penetrated. They are then removed, rinsed with water, and longitudinally sliced into pieces about 0.5 cm thick. The slices are further soaked and rinsed with water, and then a coloring liquid is applied to dye the slices a deep tea color. After being dyed, they are steamed until an oily surface and luster appear, and finally, they are dried and sun-dried. Quality control tests for FZ include appearance, thin-layer chromatography (TLC) identification, moisture content, total diester diterpenoid alkaloids, and total monoester diterpenoid alkaloids. The processing method for Wolfberry entails harvesting the red fruit of *Lycium barbarum* L. during the summer and autumn seasons, followed by hot air drying. The fruit is left to air until the skin wrinkles, after which it is sun-dried. Quality control for Wolfberry includes assessment of shape, TLC identification, moisture, total ash content, heavy metals, extractables, polysaccharides of Lycium barbarum, and betaine content. Cinnamon is processed by peeling the bark of *Neolitsea cassia* (L.) Kosterm. in the autumn and allowing it to air dry. Quality control tests for Cinnamon include appearance, TLC identification, moisture content, total ash content, volatile oil, and cinnamaldehyde content. Upon verification, the raw materials FZ (lot 20130731, YP2421601), Wolfberry (lots P20171152, P20190404), and Cinnamon (lots 171,001, Y180301) all met the quality standards of the Pharmacopoeia of China (2015).

The preparation method for FQGBG is as follows: FZ were decocted twice with water, first under a pressure of 0.10 MPa for 2 h and then at atmospheric pressure for 1 h. The filtrates from both decoctions were combined and concentrated to a relative density of 1.1 at 60°C for use. Separately, Cinnamon were ground and subjected to steam distillation for 4 h. The distilled Cinnamon oil was then complexed with β-cyclodextrin at 40°C for 2 h and set aside. The leftover Cinnamon residue and Wolfberry were extracted with hot water at 85°C twice, each for 1 h. This extract was also concentrated to a relative density of 1.1 at 60°C for later use. To finalize the formulation, the two concentrated extracts were blended with honey, potassium sorbate, and span-80. The relative density was adjusted to between 1.3 and 1.4 at 25°C to yield the final product.

FQGBG (25 g per bottle, lot number: ZAA1801/ZEA 1901S) and placebo (25 g per bottle, lot number: ZAB1801/ZEB 1901S) were provided by the Yunnan Institute of Materia Medica and Yunnan Baiyao Group Co., Ltd. (Kunming, Yunnan, China), with an expiration period of 18 months. The content of monoester alkaloids (BMA, BHA, and BAC) in the two batches of FQGBG was 241 μg/g (ZAA1801) and 116 μg/g (ZEA1901S), respectively. The HA, MA, and AC concentration of both batches were lower than the lower limit of quantitation. The two drug batches met the draft quality standards. The quality control methods for FQGBG are provided in Supplementary Material S1, which includes three types of identification fingerprinting (TLC, HPLC-UV, and UPLC-QTOF-MS), as well as methods for quantification of six aconitine alkaloids (AC, MA, HA, BAC, BMA, and BHA). Three monoester alkaloids (BAC, BMA, and BHA) are used as efficacy markers. These quality control methods comply with the requirements of the Pharmacopoeia of China (2015).

Gui-fu-di-huang-wan (360 pills per bottle, lot number: 12031565) and You-gui-wan (10 pills per box, lot number: 2013110) were purchased from Beijing Tongren Tang Science and Technology Development Co., Ltd. (Beijing, China). AC (lot 20180316, purity 98.01%) was provided by Yunnan Institute of Materia Medica. MA (lot 110799–201608, purity 98.5%), HA (lot 110789–201609, purity 99.2%), BAC (lot 111794–201705, purity 99.1%), BMA (lot 111795–201604, purity 94.0%), and BHA (lot 111796–201705, purity 98.6%) were purchased from the National Institute for Food and Drug Control (Biomedicine Industry Base, Daxing District, Beijing, China). Norverapamil hydrochloride (lot 1331-063A1, purity 99.1%, internal standard, IS) was purchased from TLC Pharmaceutical Standards (Newmarket, Ontario, Canada). Blank plasma (Bioreclamation IVT, Westbury, NY, United States) was used to prepare the standard curve and quality control samples.

### 2.2 Study approval

The study protocol was approved by the Human Research Ethics Committees of Xiyuan Hospital, China Academy of Chinese Medical Sciences (2018XL003), and the Chinese Clinical Trial Registry (A phase I clinical trial of Fuqi Gubengao, ChiCTR1800015840, registered 24 April 2018, https://www.chictr.org.cn/showprojEN.html?proj=26934). The clinical study was conducted at Xiyuan Hospital, and all clinical procedures were performed in compliance with the International Conference on Harmonization - Good Clinical Practice and the Declaration of Helsinki.

### 2.3 Participants

Forty-two healthy Chinese volunteers aged between 18 and 50 years with body mass indices (BMI) between 19.0 and 25.0 kg/m^2^ and normal physical examinations, imagological electrocardiogram examinations, and clinical laboratory tests were enrolled in the trial. The exclusion criteria are provided in Supplementary Material S1. All participants provided written informed consent before the start of the study.

### 2.4 Study design

**FIGURE 2:**
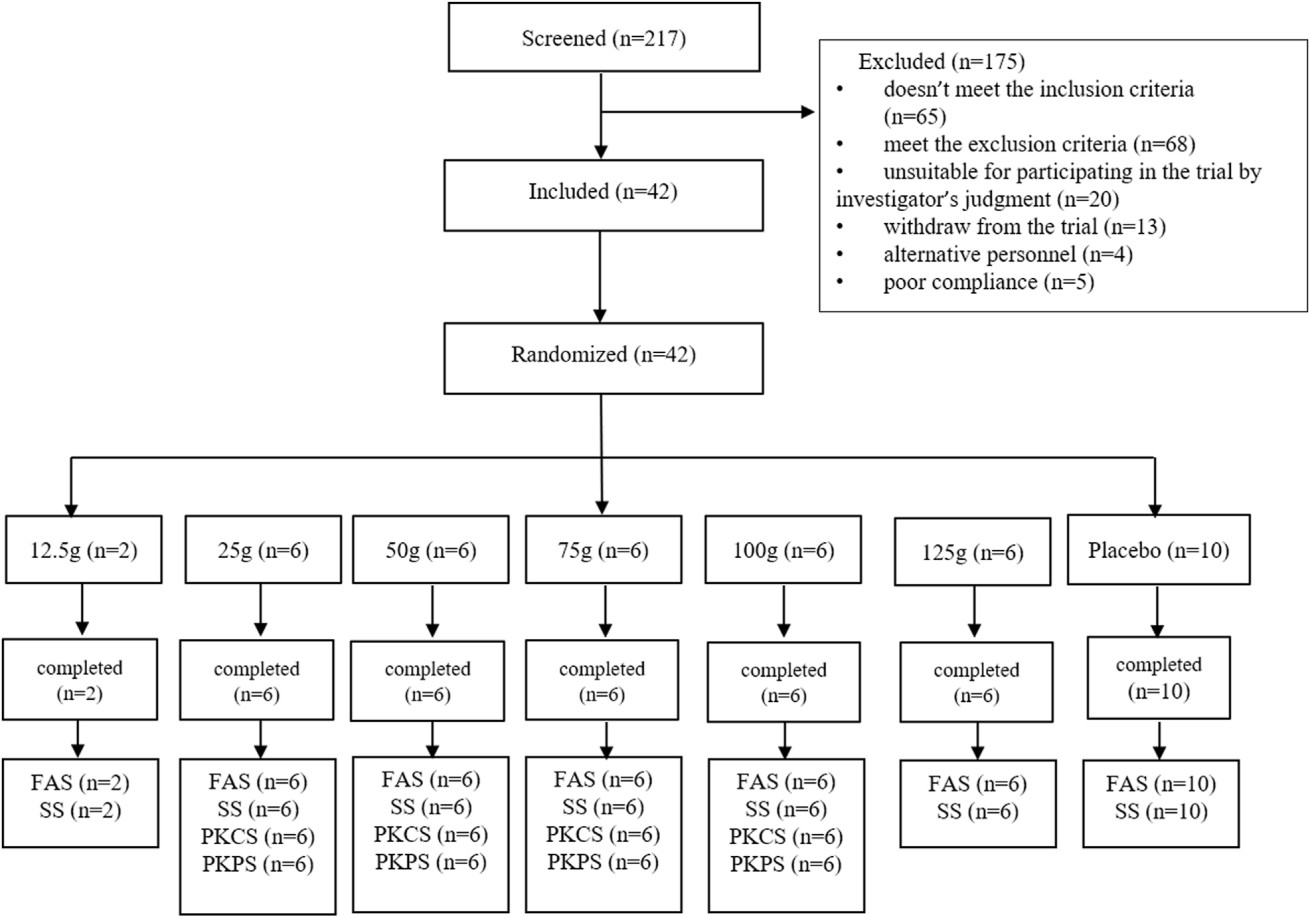
Study flowchart.

This phase I, randomized, double-blind, placebo-controlled, single ascending dose clinical trial involved six cohorts with oral doses of 12.5 g (±1 g), 25 g (±1.5 g), 50 g (±2.5 g), 75 g (±3 g), 100 g (±4.5 g), and 125 g (±7 g) FQGBG. As shown in Figure 2, the participants (*n* = 42) were allocated to the cohorts (eight participants per group, including six with FQGBG and two with placebo, at a 3:1 ratio), except for the first cohort (only two participants were treated with FQGBG). Participants were administered FQGBG or placebo at the respective dose levels with 240 mL water under fasting conditions (overnight fasting for ≥12 h). The procedures of administration were: 10 mL of water was poured into the FQGBG bottle, shook, and blended. The mixture was then poured into a paper cup, after which the above operation was repeated twice. The remaining water was poured into the paper cup and stirred evenly, and the fluid was swallowed at one time.

### 2.5 Plasma sample collection and preparation

Sampling for PKs was designed based on PK results in our previous study and collected at different time points before and after the administration of FQGBG: at 0, 5, 10, 20, 30, 45 min, 1, 2, 4, 8, 12, 24, 48, 72, 96, and 120 h in four dose groups (25, 50, 75, and 100 g). The preliminary study results indicated that the blood concentration of the three monoester-type alkaloids was reduced to 1/10th of the maximum concentration (C_max_) at 60–120 h; thus, 120 h after dosing was chosen as the last PK sampling time point. For the metabonomics analysis, blood samples were collected before and after 30 min and 24 h, and urine specimens were collected before and after 24 and 48 h to compare the differences in endogenous metabolites. Blood and urine samples were centrifuged at 1,500 ×*g* for 10 min at 4°C and the supernatant of the urine was added to preservatives. All sample supernatants were collected and stored at −80°C. Plasma (100 μL) samples spiked with 20 μL of IS were mixed evenly on a shaker. Then, 500 μL ACN containing 0.1% formic acid was added to the sample. Protein precipitation was performed by vortexing the mixture for 10 min and then centrifuging at 4,000 rpm for 10 min at 4°C. Supernatant (150 μL) was added to an equal volume of water containing 0.1% formic acid. The mixture was analyzed using a UPLC-MS/MS method.

### 2.6 LC-MS/MS conditions

A UPLC system (Waters, Milford, MA, United States) coupled with an MS/MS system (Triple Quad™ 6500, Sciex, Framingham, MA, United States) was applied for the determination of AC, MA, HA, BAC, BMA, and BHA. The data were collected and processed using Analyst software (version 1.6.3, Sciex, Framingham, MA, United States) and Watson LIMS (version 7.4.2 and 7.6SP1). Chromatographic separation was achieved using a Phenomenex Luna Omega Polar C18 column (2.1 × 50 mm, 1.6 µm) at 60°C. The mobile phase comprised water containing 5 mM ammonium acetate and 5% ACN (solvent A) and ACN containing 0.1% formic acid (solvent B). The gradient elution program for AC, MA, and HA was 0–2 min, 68%–50% A; 2–2.2 min, 50%–5% A; 2.2–2.4 min, 5% A; 2.4–2.41 min, 5%–68% A; 2.41–2.5 min, 68% A. The gradient elution program for BAC, BMA, and BHA was 0–2 min, 80%–60% A; 2–2.2 min, 60%–10% A; 2.2–2.4 min, 10% A; 2.4–2.41 min, 10%–80% A; 2.41–2.5 min, 80% A. The flow rate was 0.6 mL/min, and the injection volume was 10 µL.

The mass spectrometer was operated in the positive ion electrospray ionization mode. The analytes were detected using the multiple-reaction monitoring (MRM) mode with m/z transitions at 646.2→586.1 for AC, 632.2→572.1 for MA, 616.2→338.0 for HA, 604.2→554.2 for BA, 590.2→540.2 for BMA, 574.2→542.2 for BHA, and 441.3→165.1 for the IS. The optimized instrumental conditions were gas source temperature, 550°C; ion spray voltage, 5,500 V; entrance potential, 10 V; curtain gas, 40 psi; gas 1, 55 psi; and gas 2, 55 psi.

### 2.7 Methodological evaluation

The selectivity, calibration standard curve, matrix effect, extraction recovery, precision, accuracy, and stability of the developed method was validated according to the USA Food and Drug Administration (FDA) bio-analytical method validation guidance (U.S. Food and Drug Administration, 2018) (Supplementary Material S1). The calibration standard curves for AC, MA, HA, and BMA ranged from 0.05 to 50 ng/mL, for BAC from 0.1 to 100 ng/mL, and for BHA from 0.01 to 10 ng/mL. The UPLC-MS/MS method for AC, MA, HA, BAC, BMA, and BHA in human plasma produced the required biological quantitative standards. Therefore, this method could be used for the analysis of clinical samples.

### 2.8 Pharmacokinetic analysis

PK parameters, including the C_max_, area under the curve (AUC) from administration (*t* = 0 h) to the last measured concentration (*t* = 120 h) (AUC_0-t_), total AUC from administration to infinity (AUC_0-∞_), time to reach C_max_ (T_max_), half-life (T_1/2z_), apparent volume of distribution adjusted for bioavailability (V_z_/F), drug clearance (CL_z_), mean residence time (MRT) from administration (*t* = 0 h) to the last measured concentration (MRT_0-t_), and total MRT from administration to infinity (MRT_0-∞_) were calculated by non-compartmental methods using the WinNonlin software (version 8.1, Pharsight Corporation, Mountain View, CA, United States) with the blood concentration data of different participants at different time points.

### 2.9 Metabonomics analysis

The endogenous metabolites in the plasma and urine samples were analyzed using UPLC coupled with quadrupole time-of-flight mass spectrometry operating in MS^E^ mode (UPLC-QTOF-MS^E^). The sample pretreatment and analysis methods are detailed in Supplementary Material S1. The MassLynx™ 4.1 workstation was used for data acquisition and processing. Progenesis QI (Nonlinear Dynamics, Newcastle, United Kingdom) was used to select and align the peaks from the metabonomics raw data file. EZinfo 3.0 software (U-Metrics, Version 3.0.3.0; Waters Corporation, Wilmslow, United Kingdom) was used for principal component analysis (PCA), partial least squares-discriminant analysis (PLS-DA), and orthogonal partial least squares-discriminant analysis (OPLS-DA). The variable importance in the projection (VIP) and a Student’s t-test was used to screen for significantly changed metabolites before and after administration. Potential biomarkers were identified by comparing the tandem mass spectrometry (MS/MS) fragments with the human metabolome public database (HMDB) (http://www.hmdb.ca/) or the Mass bank database (https://massbank.us/). Metabolite pathway enrichment analysis was obtained using MetaboAnalyst 5.0 (https://www.metaboanalyst.ca/).

### 2.10 Safety evaluation

Echocardiography, B-mode ultrasound, chest radiography, breath alcohol tests, and infection screening were performed during the screening period. Routine clinical laboratory tests (blood biochemistry, routine blood, routine urine, and routine stool), blood coagulation tests, and testing for microalbuminuria, cystatin C, urine N-acetyl-β-D-glucosaminidase (NAG), troponin T, and N-terminal pro-brain natriuretic peptide (NT-pro-BNP) were performed at baseline and 24, 72, and 120 h after dosing. A 24 h Holter electrocardiogram (ECG) was performed on day 1. ECG and vital signs (blood pressure, pulse, respiratory rate, and body temperature) were recorded at baseline and each day after dosing. Urine and blood pregnancy tests in women of childbearing age were performed at baseline and on the day of completion.

### 2.11 Adverse events (AEs)

All adverse medical events after dosing, including abnormal laboratory test results, symptoms and diseases, abnormalities, and their clinical significance and relevance to the investigational product, were recorded and evaluated by investigators. Because of the known toxicity of *Aconitum carmichaelii* Debeaux, symptoms of cardiotoxicity, neurotoxicity, and gastrointestinal symptoms were closely monitored.

### 2.12 Animals

All Sprague–Dawley rats (weighing 190 ± 20 g) were purchased from Guangdong Province Medical Experimental Animal Center (Guangdong, China, license number: SCXK (Yue) 2008-0002). All rats were housed at the Animal Experiment Center of the Yunnan Institute of Materia Medica with free access to food and drinking water (room temperature 20°C–26°C, relative humidity 40%–70%, and 12 h of light/darkness). All animal protocols were approved by the Yunnan Institute of Materia Medica Ethics Committee in conformity to the guidelines for the Care and Use of Laboratory Animals by the National Institutes of Health (Approval No. 2014-08).

### 2.13 Grouping and modeling of animals

Hydrocortisone-induced KYDS model: After 5 days of acclimatization feeding, 72 male rats were randomly divided into the control group (CON), model group (MOD), Gui-fu-di-huang-wan group (GF, 4.5 g/kg), You-gui-wan group (YG, 4.8 g/kg), low-dose FQGBG group (FQGB-L, 1.29 g/kg), and high-dose FQGBG group (FQGB-H, 2.57 g/kg), with 12 rats in each group. The control group was gavaged with physiological saline, the model group was gavaged with hydrocortisone, and the treatment groups were gavaged with hydrocortisone in the morning and therapeutic drugs in the afternoon, all once daily. The modeling and drug administration were performed simultaneously for 30 consecutive days. The rat model was considered established by observing the appearance of kidney-yang deficiency symptoms in model rats, such as cold fear, huddling, hunchback, mental fatigue, emaciation, etc*.*

Natural aging KYDS model: 75 male rats were randomly divided into the model group (MOD), GF group (4.5 g/kg), YG group (4.8 g/kg), FQGB-L group (1.29 g/kg), and FQGB-H group (2.57 g/kg) after being raised to 18 months old, with 15 rats in each group. The control group was gavaged with physiological saline, whereas the other groups were gavaged with therapeutic drugs, all once daily for three consecutive months.

### 2.14 Urine volume

To investigate the effects of hydrocortisone-induced KYDS rats on urine output, rats were placed in metabolic cages for 8 h with free access to water and food at weeks 1, 2, 3, and 4 after the establishment of the model and drug administration. Average urine output was compared with the model group and statistically analyzed. For the natural aging KYDS rats, urine samples were collected for 8 h at weeks 3, 6, 8, 10, and 12 after drug administration. The average urine output was compared with the control group and was statistically analyzed.

### 2.15 Immune organ index

Following administration on day 30, the hydrocortisone-induced KYDS rats in each group were anesthetized by intraperitoneal injection of 3.5% hydrated chloral hydrate (10 mL/kg). The abdominal cavity was opened, and the adrenals, thymus, and spleen were removed. The immune organs are wiped clean with absorbent paper to remove surface blood and then weighed after removing the surrounding connective tissue. The index of the immune organs is calculated as the ratio of the weight of the immune organ to the weight of the experimental animal.

### 2.16 Statistical analysis

A descriptive analysis method was adopted to elucidate the demographic characteristics and safety indicators as number, mean, standard deviation, maximum, minimum, and median of each group. The PK parameters were expressed as the mean and standard deviation (SD). GraphPad Prism v.9.1 (GraphPad Software, Inc., San Diego, CA, United States) was used for statistical analyses and plotting. Statistical significance was set at *p <* 0.05.

## 3 Results

### 3.1 Urine volume observation

As shown in Table 1, compared with the control group, urine output in the hydrocortisone-induced KYDS model group increased at weeks 1 (8.4 ± 4.2 mL/8 h), 2 (7.8 ± 4.1 mL/8 h), and 3 (8.9 ± 3.7 mL/8 h), with significant differences. Urine output at week 4 (10.2 ± 3.4 mL/8 h) also increased, but without a significant difference. The GF and YG groups served as positive control drugs. The GF and YG group inhibited the increase in urine output of the model rats at weeks 2 (GF: 3.5 ± 2.6 mL/8 h, YG: 3.6 ± 2.3 mL/8 h), 3 (GF: 2.2 ± 2.4 mL/8 h, YG: 4.2 ± 2.3 mL/8 h), and 4 (GF: 7.4 ± 2.8 mL/8 h, YG: 6.6 ± 3.1 mL/8 h), with significant differences compared to the model group. The FQGB-L and FQGB-H groups significantly inhibited the increased urine output of the model rats from week 1 to week 4. In the first observation week, the urine output of the FQGB-L group (4.9 ± 3.1 mL/8 h) and the FQGB-H group (5.8 ± 2.1 mL/8 h) was lower than the positive control group (GF: 6.5 ± 4.3 mL/8 h, YG: 7.7 ± 3.0 mL/8 h), indicating that FQGBG acts more rapidly.

**TABLE 1:** Effects of FQGBG on urine output in hydrocortisone-induced KYDS rats.

Group	Urine volume in 8 h (mL)			
	Week 1	Week 2	Week 3	Week 4
CON	5.2 ± 1.9	4.4 ± 1.4	2.3 ± 2.2	7.8 ± 4.8
MOD	8.4 ± 4.2^▼^	7.8 ± 4.1^▼^	8.9 ± 3.7^▼^	10.2 ± 3.4
GF	6.5 ± 4.3	3.5 ± 2.6**	2.2 ± 2.4**	7.4 ± 2.8*
YG	7.7 ± 3.0	3.6 ± 2.3**	4.2 ± 2.3**	6.6 ± 3.1*
FQGB-H	5.8 ± 2.1*	4.9 ± 3.2*	4.6 ± 4.1**	6.6 ± 3.0*
FQGB-L	4.9 ± 3.1*	4.5 ± 2.5*	4.2 ± 3.2**	7.0 ± 2.6*

Values were presented as mean ± SD (*n* = 12).

^▼^ As compared with the control group (CON); ^▼^ *p <* 0.05; ^▼▼^ *p <* 0.01.

* As compared with KYDS, group (MOD); *p < 0.05; ** *p* < 0.01.

For the KYDS natural aging model rats (Table 2), the positive drugs GF and YG showed a reduction in rat urine production at weeks 10 and 12, with significant differences compared to the model group. The FQGB-L group significantly inhibited rat urine production at week 10 (4.6 ± 1.7 mL/8 h), and the FQGB-H group significantly inhibited rat urine production at weeks 3 (4.3 ± 1.9 mL/8 h), 8 (2.3 ± 1.7 mL/8 h), 10 (2.9 ± 1.3 mL/8 h), and 12 (2.1 ± 1.3 mL/8 h). This indicates that the high-dose FQGBG group has a faster onset of action than the positive control group.

**TABLE 2:** Effects of FQGBG on urine output in natural aging KYDS rats.

Group	Urine volume in 8 h (mL)				
	week 3	week 6	week 8	week 10	week 12
MOD	6.0 ± 2.1	3.9 ± 3.6	6.4 ± 2.1	6.0 ± 2.1	3.6 ± 1.7
GF	7.0 ± 3.2	2.3 ± 1.7	4.9 ± 2.6	3.3 ± 2.0**	2.1 ± 1.4*
YG	7.3 ± 3.8	3.1 ± 2.0	5.9 ± 3.4	3.7 ± 2.0**	2.0 ± 1.6*
FQGB-H	4.3 ± 1.9*	2.6 ± 2.2	2.3 ± 1.7**	2.9 ± 1.3**	2.1 ± 1.3*
FQGB-L	8.4 ± 3.9	3.8 ± 4.0	6.3 ± 3.7	4.6 ± 1.7*	2.2 ± 2.0

Values were presented as mean ± SD (*n* = 15).

*As compared with KYDS, group (MOD); *p < 0.05; **p < 0.01.

### 3.2 Change of immune organ index

As shown in Table 3, compared with the control group, the adrenal gland (0.088 ± 0.014 mg/g), the thymus (0.78 ± 0.14 mg/g), and the spleen (1.32 ± 0.15 mg/g) showed significant atrophy in hydrocortisone-induced KYDS rats. GF, YG, and FQGBG all had antagonistic effects on adrenal atrophy, but only the FQGB-H group (0.100 ± 0.010 mg/g) showed statistically significant efficacy. GF, YG, and FQGB-H mildly antagonized hydrocortisone-induced thymus atrophy, but there were no significant differences. YG and FQGBG also mildly antagonized hydrocortisone-induced splenic atrophy, but there were no significant differences.

**TABLE 3:** Effects of FQGBG on the immune organ index in hydrocortisone-induced KYDS rats.

Group	Adrenals (mg/g)	Thymus (mg/g)	Spleen (mg/g)
CON	0.132 ± 0.014	1.60 ± 0.32	1.82 ± 0.14
MOD	0.088 ± 0.014^▼▼^	0.78 ± 0.14^▼▼^	1.32 ± 0.15^▼▼^
GF	0.098 ± 0.013	0.90 ± 0.28	1.27 ± 0.18
YG	0.094 ± 0.010	0.83 ± 0.20	1.43 ± 0.43
FQGB-H	0.100 ± 0.010*	0.80 ± 0.24	1.37 ± 0.19
FQGB-L	0.103 ± 0.018	0.69 ± 0.18	1.48 ± 0.18

Values were presented as mean ± SD (*n* = 12).

^▼^ As compared with the control group (CON); ^▼^ *p* < 0.05; ^▼▼^ *p* < 0.01.

* As compared with KYDS, group (MOD); *p < 0.05; **p < 0.01.

### 3.3 Pharmacokinetics results

**FIGURE 3:**
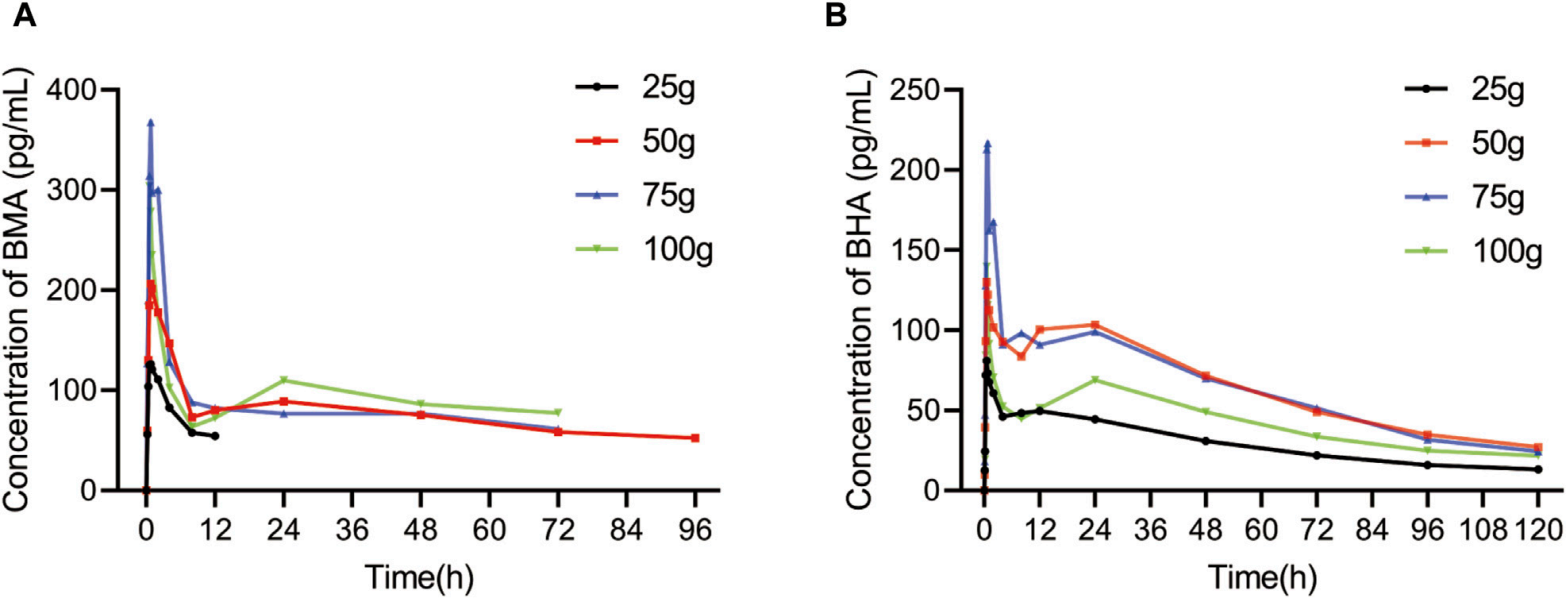
Average blood drug concentration-time of BMA **(A)** and BHA **(B)**.

Among all participants, 32 healthy Chinese volunteers in the 25, 50, 75, and 100 g groups were included in the PK study. The mean plasma concentration vs. time profiles of BMA and BHA are shown in Figure 3, and the corresponding major PK parameters are shown in Table 4. The plasma concentrations of AC, MA, HA, and BAC were all below the lower limit of quantification (LLOQ) (50 pg/mL for AC, MA, and HA; 100 pg/mL for BAC). The C_max_ of BMA (C_max_: 138.57–409.00 pg/mL) was higher than BHA (C_max_: 84.37–245.67 pg/mL), whereas the AUC_0-t_ of BMA (AUC_0-t_: 625.02–4283.40 h × pg/mL) was lower than BHA (AUC_0–t_: 3471.15–7627.07 h × pg/mL). The results indicate that the overall exposure level of BHA was higher than BMA. The AUC_0–t_ values of the 50 and 75 g groups of BMA and BHA were similar, suggesting that a saturable absorption was reached after the 50 g treatment. The AUC_0–t_ values of the 100 g group decreased slightly, reflecting the differences in the drug batches. The T_max_ of BMA (T_max_: 0.88–1.38 h) was faster than BHA (T_max_: 0.76–6.38 h), whereas the half-life of BMA (T_1/2_: 14.24–64.03 h) was shorter than BHA (T_1/2_: 43.59–86.27 h). The power function model was used to analyze the associations between PK parameters and the dose in BMA and BHA (Supplementary Figures S2–S4; Supplementary Table S2). The results showed that BMA had linear PK characteristics in the dose range of 25–100 g, and the exposure increase ratio of BHA from 25 to 100 g was lower than the dose increase ratio.

**TABLE 4:** Major PK parameters of BMA and BHA at doses of 25–100 g (Mean ± SD).

Analyte	Parameter	Group of doses			
		25 g	50 g	75 g	100 g
BMA	C_max_ (pg/mL)	138.57 ± 39.25	216.67 ± 75.26	409.00 ± 268.30	321.33 ± 208.62
	AUC_0-t_ (h×pg/mL)	625.02 ± 395.36	4,102.95 ± 3,026.13	4,283.40 ± 2,200.76	2,710.99 ± 2,723.85
	AUC_0-∞_ (h×pg/mL)	1,963.07 ± 739.08	7,813.99 ± 5,017.74	10,119.87 ± 4,345.04	6,101.52 ± 6,360.16
	T_max_ (h)	1.13 ± 0.70	1.00 ± 0.52	1.38 ± 0.68	0.88 ± 0.56
	T_1/2z_ (h)	14.24 ± 10.26	44.85 ± 36.03	64.03 ± 28.05	32.28 ± 39.99
	V_z_/F (×10^8^ L)	2.40 ± 0.93	3.76 ± 1.15	6.84 ± 1.81	5.82 ± 3.74
	CL_z_/F (×10^7^ L/h)	1.35 ± 0.40	1.04 ± 0.95	0.89 ± 0.51	5.27 ± 4.74
	MRT_0-t_ (h)	3.19 ± 1.40	19.33 ± 13.15	17.42 ± 10.58	12.09 ± 13.23
	MRT_0-∞_ (h)	20.19 ± 14.26	63.93 ± 49.32	87.39 ± 42.24	47.33 ± 56.07
BHA	C_max_ (pg/mL)	84.37 ± 25.69	149.33 ± 49.52	245.67 ± 167.69	148.03 ± 91.16
	AUC_0-t_ (h×pg/mL)	3,471.15 ± 467.60	7,634.24 ± 3,263.18	7,627.07 ± 2,604.34	4,831.67 ± 2,780.52
	AUC_0-∞_ (h×pg/mL)	5,150.81 ± 1,682.08	9,744.52 ± 4,764.55	9,151.92 ± 3,109.76	6,136.92 ± 3,431.34
	T_max_ (h)	0.76 ± 0.62	6.38 ± 9.77	4.96 ± 9.35	4.46 ± 9.57
	T_1/2z_ (h)	86.27 ± 79.27	49.67 ± 10.28	43.59 ± 4.75	47.72 ± 12.18
	V_z_/F (×10^8^ L)	5.27 ± 2.50	4.04 ± 1.24	5.59 ± 2.03	13.36 ± 4.55
	CL_z_/F (×10^7^ L/h)	0.51 ± 0.12	0.60 ± 0.27	0.88 ± 0.28	2.04 ± 0.89
	MRT_0-t_ (h)	43.10 ± 4.89	43.45 ± 6.32	42.81 ± 4.53	41.74 ± 10.29
	MRT_0-∞_ (h)	114.50 ± 89.49	73.01 ± 16.45	66.29 ± 5.34	71.01 ± 20.86

### 3.4 Metabonomics results

**FIGURE 4:**
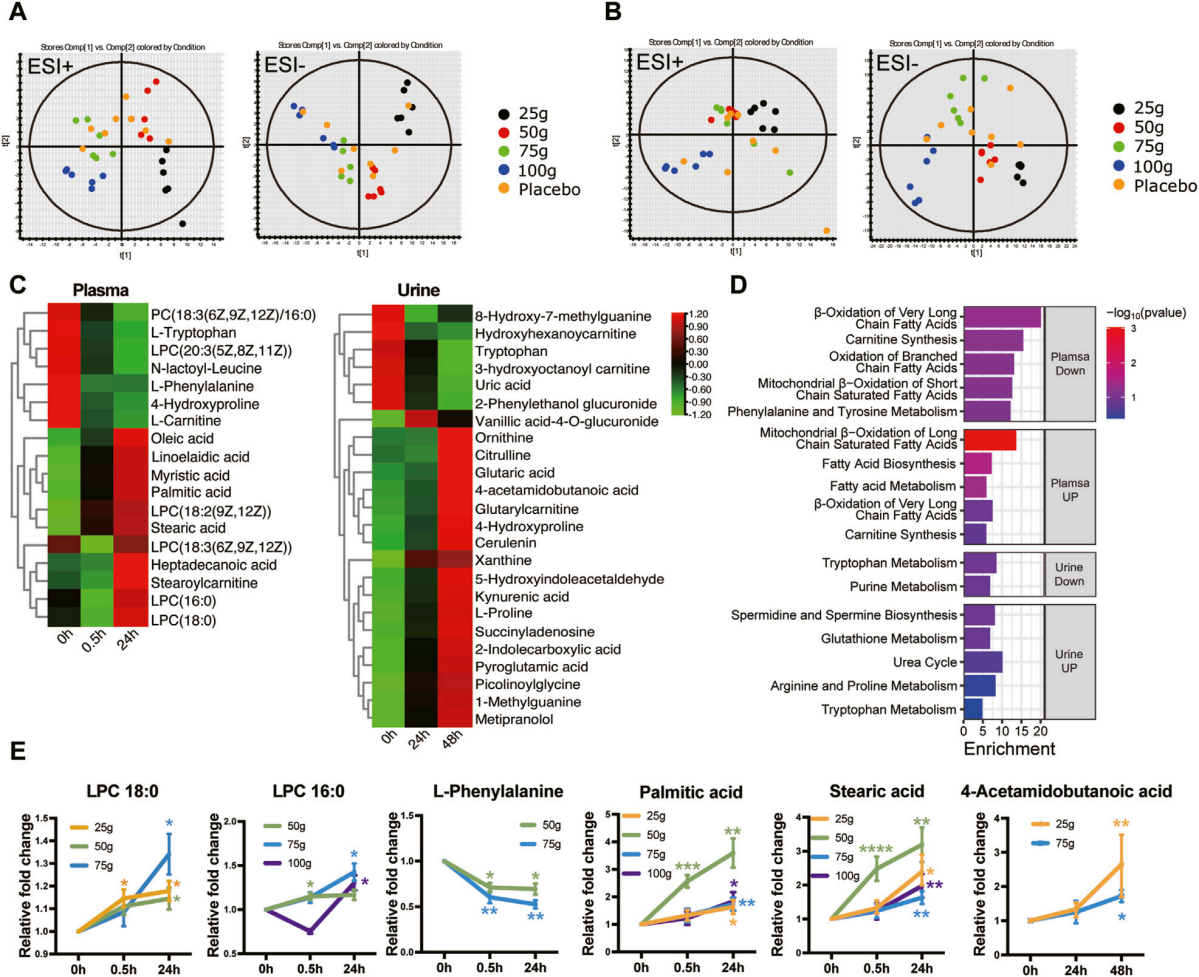
Metabonomic characteristics induced by the different dosages of FQGBG. **(A)** PLS-DA scoring plot of plasma collected 24 h after treatment in positive and negative ion modes. (ESI + scores: R2Y = 0.366, Q2 = 0.223; ESI- scores: R2Y = 0.341, Q2 = 0.130). **(B)** PLS-DA scoring plot of urine collected 48 h after treatment in both positive and negative ion modes (ESI + scores: R2Y = 0.655, Q2 = 0.093; ESI- scores: R2Y = 0.720, = 0.232). **(C)** Heat maps illustrate the time-dependent changes of abundance levels of metabolites. **(D)** Metabolite pathway enrichment analysis of upregulated and downregulated biomarkers in plasma and urine, respectively. **(E)** Commonly changed metabolites induced by the different dosages of FQGBG at different time points. *p < 0.05, **p < 0.01, ***p < 0.001.

PCA and PLS-Da maps were used to determine whether the administration group was well discriminated from the placebo group. As shown in Figures 4A, B and Supplementary Figure S3, the plasma (24 h post-administration) and urine (48 h post-administration) results in each dose group could not be separated from the placebo group; nevertheless, they were well-differentiated among the dose groups. This may be related to the small effect of a single administration on metabolome changes. To search for differential metabolites, metabolites with a VIP ≥1 and *p <* 0.05 were screened by OPLS-DA. FQGBG treatment of 25, 50, 75 and 100 g produced 19, 10, 10 and 6 biomarkers, respectively (Table 5). Among these, the common biomarkers in the different dosage groups were LPC18:0, LPC16:0, L-phenylalanine, palmitic acid, and stearic acid in the plasma sample and 4-acetamidobutanoic acid in the urine sample. The heat map displayed the regulation of biomarkers in the plasma and urine before and after administration (Figure 4C). Upregulated biomarkers in plasma were mainly free fatty acids and LPCs, with metabolic pathway enrichment in mitochondrial β-oxidation of long-chain saturated fatty acids (Figure 4D). Among them, LPC18:0, LPC16:0, palmitic acid, and stearic acid were significantly upregulated in multiple treatment groups (Figure 4E). Downregulated metabolites included L-carnitine, with pathway enrichment mainly focused on fatty acid oxidation-related pathways. This indicated that the changes in plasma metabolites were mainly associated with fatty acid metabolism, mitochondrial β-oxidation, and energy metabolism. In addition, phenylalanine was significantly downregulated in plasma (Figure 4E), suggesting that FQGBG may lead to phenylalanine consumption. In urine, the secondary metabolite of arginine, 4-acetamidobutyric acid, was upregulated after administration (Figure 4E). Furthermore, in the 25 g dose group, ornithine, citrulline, proline, and 4-hydroxyproline were all increased, indicating that the metabolism pathways of arginine and proline may be influenced by FQGBG. Pathway enrichment analysis revealed that biomarkers in urine were mainly associated with tryptophan, purine, arginine, and proline metabolisms, and the urea cycle. In the high-dose group, these biomarkers showed more significant changes (Supplementary Figure S6), suggesting that these metabolites may contribute to understanding the safety and pharmacological effects of FQGBG. All biomarkers were identified by comparison with secondary spectrograms in the HMDB and Massbank databases (Supplementary Figure S7), with two or more characteristic fragment ions as the basis for identification and validation.

**TABLE 5:** Altered metabolite levels after FQGBG treatment.

Group (g)	No.	m/z	Error (ppm)	Ion	Retention time (min)	Identify	Molecular formula	Sample	*p*-value	Trend
25	1	428.3698	−9.66	ESI+	10.17	Stearoylcarnitine	C_25_H_49_NO_4_	Plasma	0.0004	↑
	2	524.3633	−8.51	ESI+	10.96	LPC (18:0)	C_26_H_54_NO_7_P	Plasma	0.0464	↑
	3	518.3160	−7.42	ESI+	8.86	LPC (18:3 (6Z,9Z,12Z))	C_26_H_48_NO_7_P	Plasma	0.0328	↑
	4	546.3476	−8.19	ESI+	9.85	LPC (20:3 (5Z,8Z,11Z))	C_28_H_52_NO_7_P	Plasma	0.0042	↓
	5	281.2488	0.10	ESI-	10.12	Oleic acid	C_18_H_34_O_2_	Plasma	0.0212	↑
	6	283.2647	0.78	ESI-	10.96	Stearic acid	C_18_H_36_O_2_	Plasma	0.0221	↑
	7	255.2327	−1.98	ESI-	9.81	Palmitic acid	C_16_H_32_O_2_	Plasma	0.0456	↑
	8	144.0658	−7.58	ESI-	3.00	4-Acetamidobutanoic acid	C_16_H_32_O_2_	Urine	0.0020	↑
	9	133.0976	5.85	ESI+	1.02	Ornithine	C_5_H_12_N_2_O_2_	Urine	0.0051	↑
	10	116.0709	4.83	ESI+	1.01	L-Proline	C_5_H_9_NO_2_	Urine	0.0068	↑
	11	203.0819	−4.90	ESI-	4.49	Tryptophan	C_11_H_12_N_2_O_2_	Urine	0.0075	↓
	12	169.0359	3.32	ESI+	1.47	Uric acid	C_5_H_4_N_4_O_3_	Urine	0.0108	↓
	13	166.0728	4.83	ESI+	3.44	1-Methylguanine	C_6_H_7_N_5_O	Urine	0.0153	↑
	14	384.1156	2.32	ESI+	3.93	Succinyladenosine	C_14_H_17_N_5_O_8_	Urine	0.0169	↑
	15	176.0713	5.66	ESI+	6.28	5-Hydroxyindoleacetaldehyde	C_10_H_9_NO_2_	Urine	0.0229	↑
	16	162.0555	4.75	ESI+	4.79	2-Indolecarboxylic acid	C_9_H_7_NO_2_	Urine	0.0254	↑
	17	188.0344	−6.35	ESI-	4.78	Kynurenic acid	C_10_H_7_NO_3_	Urine	0.0305	↑
	18	131.0344	−6.82	ESI-	3.77	Glutaric acid	C_5_H_8_O_4_	Urine	0.0311	↑
	19	132.0659	5.43	ESI+	1.18	4-Hydroxyproline	C_5_H_9_NO_3_	Urine	0.0415	↑
50	1	524.3633	−8.51	ESI+	10.96	LPC (18:0)	C_26_H_54_NO_7_P	Plasma	0.0231	↑
	2	166.0862	1.44	ESI+	2.51	L-Phenylalanine	C_9_H_11_NO_2_	Plasma	0.0441	↓
	3	496.3357	−7.59	ESI+	9.78	LPC (16:0)	C_24_H_50_NO_7_P	Plasma	0.0228	↑
	4	269.2492	0.98	ESI-	10.38	Heptadecanoic acid	C_17_H_34_O_2_	Plasma	0.0112	↑
	5	283.2647	0.78	ESI-	10.96	Stearic acid	C_18_H_36_O_2_	Plasma	0.0045	↑
	6	227.2009	−4.26	ESI-	8.70	Myristic acid	C_14_H_28_O_2_	Plasma	0.0221	↑
	7	279.2325	−2.54	ESI-	9.42	Linoelaidic acid	C_18_H_32_O_2_	Plasma	0.0074	↑
	8	255.2327	−1.98	ESI-	9.81	Palmitic acid	C_16_H_32_O_2_	Plasma	0.0077	↑
	9	182.0678	4.73	ESI+	3.18	8-Hydroxy-7-methylguanine	C_6_H_7_N_5_O_2_	Urine	0.0334	↓
	10	304.2125	2.92	ESI+	6.60	3-Hydroxyoctanoyl carnitine	C_15_H_29_NO_5_	Urine	0.0460	↓
75	1	132.0661	5.43	ESI+	0.86	4-Hydroxyproline	C_5_H_9_NO_3_	Plasma	0.0068	↓
	2	524.3633	−8.51	ESI+	10.96	LPC (18:0)	C_26_H_54_NO_7_P	Plasma	0.0272	↑
	3	756.5519	−2.11	ESI+	12.45	PC (18:3 (6Z,9Z,12Z)/16:0)	C_42_H_78_NO_8_P	Plasma	0.0218	↓
	4	166.0862	1.44	ESI+	2.51	L-Phenylalanine	C_9_H_11_NO_2_	Plasma	0.0028	↓
	5	205.0978	5.08	ESI+	3.20	L-Tryptophan	C_11_H_12_N_2_O_2_	Plasma	0.0063	↓
	6	496.3357	−7.59	ESI+	9.78	LPC (16:0)	C_24_H_50_NO_7_P	Plasma	0.0321	↑
	7	283.2647	0.78	ESI-	10.96	Stearic acid	C_18_H_36_O_2_	Plasma	0.0065	↑
	8	255.2327	−1.98	ESI-	9.81	Palmitic acid	C_16_H_32_O_2_	Plasma	0.0069	↑
	9	151.0256	−5.26	ESI-	2.04	Xanthine	C_5_H_4_N_4_O_2_	Urine	0.0033	↑
	10	144.0658	−7.58	ESI-	3.00	4-Acetamidobutanoic acid	C_6_H_11_NO_3_	Urine	0.0294	↑
100	1	520.3328	−9.00	ESI+	9.42	LPC (18:2 (9Z,12Z))	C_26_H_50_NO_7_P	Plasma	0.0011	↑
	2	496.3357	−7.59	ESI+	9.78	LPC (16:0)	C_24_H_50_NO_7_P	Plasma	0.0429	↑
	3	283.2647	0.78	ESI-	10.96	Stearic acid	C_18_H_36_O_2_	Plasma	0.0096	↑
	4	255.2327	−1.98	ESI-	9.81	Palmitic acid	C_16_H_32_O_2_	Plasma	0.0212	↑
	5	224.1288	4.70	ESI+	9.02	Cerulenin	C_12_H_17_NO_3_	Urine	0.0289	↑
	6	130.0505	6.97	ESI+	7.26	Pyroglutamic acid	C_5_H_7_NO_3_	Urine	0.0086	↑

### 3.5 Demographics and safety

Overall, 217 participants were screened, 175 were excluded, and 42 were included. Two participants were assigned to the 12.5 g group, and 30 participants were randomly assigned to the 25, 50, 75, 100, and 125 g groups, respectively (Figure 2). Ten participants were assigned to the placebo group. All participants completed the trial and were included in the full analysis set (FAS) and safety analysis set (SS). In addition, 32 participants in the 25, 50, 75, and 100 g groups, who also participated in the PK study, were included in the pharmacokinetics concentration set (PKCS) and pharmacokinetics parameter set (PKPS) analyses. The detailed demographic information and AEs are listed in Tables 6, 7 and Supplementary Table S1. The safety analysis of treatment-emergent adverse events (TEAEs) by system shows varying incidence rates across different dose groups and the placebo. The most common TEAEs included gastrointestinal disorders, with rates of 50% in the 12.5 g group, 33.3% in the 125 g group, and 20% in the placebo group. Investigations of abnormal laboratory results were also notable, especially elevated NAG enzyme levels (33.3%) and positive urine protein (33.3%) in the 75 g group. Cardiac disorders, particularly ventricular extrasystoles, were observed in the 12.5 g (50%) and 25 g (16.7%) groups. General disorders such as heat sensation were frequent in the 125 g group (66.7%). However, the overall incidence of TEAEs and adverse drug reactions (ADRs) were 56.3% in the treatment groups compared to 50% in the placebo group, with no statistical difference observed between the two groups.

**TABLE 6:** Demographics of participants.

	12.5 g	25 g	50 g	75 g	100 g	125 g	Placebo
*N*	2	6	6	6	6	6	10
Age (years, mean ± SD)	43.5 ± 2.12	36.0 ± 3.69	27.7 ± 1.75	28.0 ± 3.74	30.8 ± 3.13	28.2 ± 2.64	31.6 ± 3.53
Male (*n*, %)	1 (50.0)	3 (50.0)	3 (50.0)	3 (50.0)	3 (50.0)	3 (50.0)	5 (50.0)
Female (*n*, %)	1 (50.0)	3 (50.0)	3 (50.0)	3 (50.0)	3 (50.0)	3 (50.0)	5 (50.0)
Height (cm, mean ± SD)	159.00 ± 1.84	163.73 ± 10.2	168.82 ± 4.66	163.13 ± 7.15	167.25 ± 9.08	163.57 ± 8.92	161.21 ± 8.49
Weight (kg, mean ± SD)	56.750 ± 7.92	62.500 ± 9.81	63.550 ± 9.43	56.450 ± 7.39	62.058 ± 8.04	58.925 ± 7.18	57.705 ± 5.78
BMI (kg/m^2^, mean ± SD)	22.45 ± 2.62	23.20 ± 1.61	22.20 ± 2.35	21.13 ± 1.23	22.13 ± 1.81	22.03 ± 2.33	22.20 ± 1.43

*N* and % represent the number and percentage of subjects; BMI, body mass index; SD, standard deviation.

**TABLE 7:** Occurrence rate of AEs.

	12.5 g	25 g	50 g	75 g	100 g	125 g	Total	Placebo
*N*	2	6	6	6	6	6	32	10
TEAE *n* (%)	1 (50.0)	2 (33.3)	2 (33.3)	5 (83.3)	3 (50.0)	5 (83.3)	18 (56.3)	5 (50.0)
ADR *n* (%)	1 (50.0)	2 (33.3)	2 (33.3)	5 (83.3)	3 (50.0)	5 (83.3)	18 (56.3)	5 (50.0)
SAE *n* (%)	0 (0)	0 (0)	0 (0)	0 (0)	0 (0)	0 (0)	0 (0)	0 (0)
SADR *n* (%)	0 (0)	0 (0)	0 (0)	0 (0)	0 (0)	0 (0)	0 (0)	0 (0)

TEAE, treatment-emergent adverse event; ADR, adverse drug reaction; SAE, serious adverse event; SADR, serious adverse drug reaction; Relationships to FQGBG that refer to confirm, probability, and suspicion are defined as ADR.

## 4 Discussion

FQGBG contains diterpenoid alkaloids, including diester, monoester, and lipid alkaloids that are both active and toxic. The diester alkaloids have the highest toxicity, and the literature indicates that cardiotoxicity and neurotoxicity are the major toxic reactions of diester diterpenoid alkaloids in *Aconitum carmichaelii* Debeaux (Yang et al., 2018). The AE related to cardiac damage was ventricular premature beat in the 12.5 and 25 g groups. Considering that ventricular premature beats existed at baseline, and there was no remarkable increase in the frequency of ventricular premature beats after dosing or an increased occurrence frequency with dose escalation, the conclusion that the ventricular premature beat was caused by the medication could not be drawn. Other AEs mainly manifested as abnormal laboratory results, but the severity was mild, and AEs had an optimistic prognosis. Elevated NAG enzyme levels and positive urine protein in the 75 g group suggest a possible link to FQGBG’s diterpenoid alkaloids, which can be nephrotoxic. These findings may indicate renal stress or damage, particularly at higher doses. In the practical application of TCM, the clinical timing of using “toxic” herbal pieces is particularly crucial. Although the toxic plant metabolites present a risk of harming the body, they can yield significant therapeutic effects when used appropriately. Therefore, the potential renal damage caused by FQGBG might be due to individual intolerance when the drug is applied to healthy subjects, emphasizing the need for careful monitoring and appropriate clinical use. Other AEs mainly manifested as abnormal laboratory results, but the severity was mild, and AEs had an optimistic prognosis. Some AEs may be related to changes in the diet and lifestyle during hospitalization; therefore, whether these AEs were caused by FQGBG, or other reasons, is uncertain and further research is needed. FQGBG warms and invigorates the kidney yang; therefore, the primary AEs, including thirst, heat sensation, gum pain, aphtha, feverish palms, insomnia, diarrhea, and abdominal distension, were likely due to the injured body fluid by excessively tonifying the yang and deficient fire flare-ups.

Aconitine alkaloids are the main toxic metabolites and active metabolites of FZ, which have good anti-inflammatory, analgesic, and cardiotonic effects and are widely used in the treatment of various diseases. Among them, a series of diterpenoid alkaloids such as AC, MA, and HA have strong neurotoxic and cardiotoxic effects. In the extraction and preparation process, prolonging high-temperature decoction time can promote the degradation of diester alkaloids into monoester alkaloids, reducing the toxicity and improving the efficacy of the drug (Zhang et al., 2016). When patients experience toxic reactions induced by FZ, the blood concentrations of AC, MA, and HA can reach 0.031, 0.086, and 0.125 ng/mL (Zhang et al., 2019), respectively, which are 0.6, 1.72, and 2.5 times the LLOQ of AC, MA, and HA obtained in this study, whereas the blood concentrations of the three diester alkaloids in this study were all lower than the LLOQ. Furthermore, the blood concentration of the monoester alkaloid, BMA, in FZ poisoning patients, reached 4.665 ng/mL, which is 11.4 times the C_max_ of BMA in this study. The above evidence demonstrates the safety of FQGBG at the studied doses.

The PK behavior of monoester alkaloids of FZ exhibits differences. In this study, the blood concentration of BAC was lower than the LLOQ, which may be related to its fast absorption (T_max_: 0.31 ± 0.17 h) and low bioavailability (Zhang et al., 2016). BMA is the most abundant monoester alkaloid in FZ, with analgesic and anti-inflammatory effects, and is a potential drug to treat inflammation-related diseases (Zhou et al., 2022). The PK results indicated that BMA is eliminated slowly and that the T1/2z of BMA in the 25–75 g dosage group showed a certain dose dependence (Liu et al., 2014). This suggests that the metabolic enzymes or transporters responsible for BMA’s clearance could become saturated at higher doses. This saturation may be related to the influence of other metabolites in FQGBG on the activity of the BMA-metabolizing enzyme CYP3A4 (Dai et al., 2014). Furthermore, Vz/F of BMA gradually increased in the 25–75 g groups, suggesting that higher doses increased the distribution of BMA in the peripheral compartment, which may be related to the increase in T_1/2_.

In contrast, this situation was not observed for BHA, indicating that BHA’s metabolic pathways do not experience the same level of saturation within the studied dosage range. Additionally, significant interindividual variability was observed in the PK parameters of BMA and BHA in healthy participants. Our analysis revealed that the CV for most PK parameters of both BMA and BHA exceeded 30%. It is might partly be attributed to the relatively small sample size in our study. A larger sample size would provide a more robust dataset, potentially allowing for a more accurate assessment of the variability and its clinical implications. This study found that the C_max_, AUC_0–t_, and AUC_0–∞_ of BMA and BHA in the 100 g group were lower than those in the 75 g group. One possible reason for this was that the monoester alkaloid content in the new batch (116 μg/g) was lower than that in the previous batch (241 μg/g) during the clinical trial.

Metabonomics was used to observe the changes in endogenous metabolites in healthy participants after drug administration to evaluate the safety of drugs in humans and explore potential therapeutic targets of drugs. AC is the main plant metabolites in FQGBG, and owing to its known toxicity, it was the focus of the drug safety analysis in this study. Phenylalanine is converted mainly into tyrosine by phenylalanine hydroxylase in the liver; therefore, when MA causes liver damage, this physiological transformation is blocked, resulting in elevated phenylalanine levels (Chen et al., 2022). The upregulation of phenylalanine and tyrosine levels was also associated with inflammation in chronic heart failure, suggesting that phenylalanine can also be an indicator of cardiac function (Cheng et al., 2021). However, our study found that the phenylalanine level was significantly reduced in the 50 and 75 g treatment groups, which may characterize the safety of FQGBG in healthy individuals. AC poisoning also upregulates various amino and fatty acids (Zhang et al., 2019). The abnormal peroxidation of polyunsaturated fatty acids such as arachidonic acid and its metabolites can damage the myocardial mitochondria, leading to cardiac arrhythmia (Cai et al., 2013). However, similar results were not obtained in this study. Therefore, combined with clinical AE observations, metabonomics results can supplement the elucidation of the good safety and tolerability of FQGBG.

The pathogenesis of KYDS involves mainly dysfunction of the hypothalamic-pituitary-target gland axis (adrenal, thyroid, and gonad) marked by cold limbs, polyuria, and slow responses (Chen et al., 2019). Pharmacological results demonstrate that FQGBG can alleviate increased urination in rats with two models of KYDS models (hydrocortisone-induced model and natural aging model) and its efficacy is faster compared to other commonly used TCMs (Gui-fu-di-huang-wan, You-gui-wan) to treat KYDS. In addition, it effectively counteracts hydrocortisone-induced adrenal atrophy in KYDS rat models by hydrocortisone, which suggest that FQGBG primarily exerts its therapeutic effects by counteracting the adrenal dysfunction caused by KYDS, repairing the function of the hypothalamus-pituitary-adrenal axis, and this may be related to the restoration of adrenal responsiveness to pituitary hormones, promoting the release of cortisol and aldosterone, which regulate the water and sodium balance in the kidney. In contrast, altered metabolites in urine, including acetylamino butyric acid, ornithine, guanidinoacetic acid, proline, and 4-hydroxyproline, are all associated with the arginine metabolism pathway. Arginine can regulate the release of adrenocortical hormones and cortisol in patients with diabetes insipidus (Bologna et al., 2020), which may be related to the improvement of polyuria symptoms by FQGBG in KYDS.

Observation of experimental animals, both the FQGBG and the positive drug group improved the symptoms of cold fear, huddling, hunchback, and mental fatigue. This indicates that FQGBG and the positive drug have a certain alleviating effect on the symptoms of decreased energy metabolism and insufficient heat production caused by KYDS. FZ and Cinnamon in FQGBG are TCM that generate heat in the interior body and eliminates cold. Alkaloids in FZ, including fuziline, neoline, and BMA, are identified as β-adrenergic receptor (β-AR) agonists, for regulating the thermogenic function and regulate glucose and lipid metabolism by activating the β3-AR receptor of adipocytes (Gao et al., 2023). Cinnamaldehyde, the main metabolite of Cinnamon, activated PKA signaling, increased thermogenic gene expression levels, and induced phosphorylation of hormone-sensitive lipase (HSL) and Perilipin 1 in primary murine primary adipocytes (Jiang et al., 2017). Therefore, in this study, elevated levels of palmitic acid and stearic acid, which are the main substrates for lipid breakdown and beta-oxidation, may be associated with enhanced lipid breakdown and enhanced energy metabolism induced by FQGBG. Furthermore, upregulation of palmitic acids and stearic acid may also be associated with increased secretory phospholipase (sPLA) activity and degradation of glycerophospholipids. sPLA has been reported to increase the expression of mitochondrial uncoupling markers such as UCP1 and PPAR in brown and white adipocytes of mice (25, 26), thus enhancing mitochondrial oxidation and thermogenesis. sPLA regulates lipolysis in adipocytes by enhancing HSL activity through the ERK signaling pathway (Zhi et al., 2015).

## 5 Conclusion

The PK and safety of single-dose oral administration of FQGBG were investigated for the first time for healthy Chinese volunteers. The results indicated that the half-lives of FZ monoester alkaloids were prolonged, and there were significant individual differences. Therefore, further evaluation of drug accumulation is needed in PK studies involving continuous administration. Under the experimental dosing regimen, FQGBG was safe in healthy subjects. The pharmacodynamics and metabonomics results suggest that FQGBG exerts its efficacy by improving the function of the hypothalamus-pituitary-adrenal axis, effectively treating the increased urination caused by KYDS, and alleviating the symptoms of cold fear in KYDS rats through enhancing energy metabolism.

## Data Availability

The original contributions presented in the study are included in the article/Supplementary Material, further inquiries can be directed to the corresponding authors.
