# Supplementary material for: Pharmacokinetics, safety, and efficacy of Fuqi Guben Gao in the treatment of kidney-yang deficiency syndrome: a randomized, double-blind phase I trial
Source: Front Pharmacol. 2024 Jun 25;15:1351871. doi: 10.3389/fphar.2024.1351871 (PMC11250459; doi:10.3389/fphar.2024.1351871)
Supplement: Supplementary file 1 [file DataSheet1.docx]

Supplementary Material

A randomized, double-blind, single-escalating dose study to determine the tolerability, pharmacokinetics, and metabonomics of Fuqi Guben Gao in healthy participants

1. **Supplementary Materials S1**

**1.1 Triple chemical fingerprinting and quality control**

**1.1.1 UPLC-QTOF-MS fingerprinting of FQGBG**

100 μL FQGBG samples were diluted with 200 μl water, treated with 3 μL formic acid (Sample 1) or 20 μL 30% ammonia (Sample 2), then extracted with 300 μL ethyl acetate for liquid-liquid extraction, and centrifuged at 12000 r/min for 10 min after vortex oscillation. The upper liquid was dried with nitrogen at room temperature and redissolved with 100 μL methanol. 2 μL of the sample was injected into an Acquity UPLC HSS T3 column (2.1 mm × 100 mm, 1.8 μm, Waters, Milford, MA, USA), and was analyzed by the ultra-performance liquid chromatography with quadrupole time-of-flight mass spectrometry instrument (UPLC-QTOF-MS).

Eleven components of FQGBG were identified, as shown in Table S4. Benzoylaconitine, cinnamaldehyde, cinnamic acid, coumarin, and caffeic acid were identified as standard substances and characteristic fragments. Hypaconitine (HA), mesaconitine (MA), benzoylhypaconitine (BHA), benzoylmesaconitine (BMA), cinnamyl alcohol, and betaine were identified based on references and the Massbank database in Fig. S8-S9.

Table S4. Components in FQGBG

| Peak | Component | Formula | Time  (min) | Ion type | Measured mass(m/z) | Error  (ppm) | Fragments | Source herb | Reference |
| --- | --- | --- | --- | --- | --- | --- | --- | --- | --- |
| 1 | Betaine | C_5_H_11_NO_2_ | 0.78 | [M+H]^+^ | 118.0855 | -6.77 | 59.0751, 58.0652 | Wolfberry | Massbank |
| 2 | Caffeic acid | C_9_H_8_O_4_ | 3.82 | [M-H]^-^ | 179.0357 | 4.47 | 135.0459 | Wolfberry | Standard |
| 3 | Benzoylmesaconitine | C_31_H_43_NO_10_ | 5.30 | [M+H]^+^ | 590.2899 | -3.39 | 540.2676, 508.2415, 105.0358 | FuZi | Liu et al., 2019 |
| 4 | Benzoylaconitine | C_32_H_45_NO_10_ | 5.62 | [M+H]^+^ | 604.3071 | -7.52 | 554.2603, 552.2348, 105.0314 | FuZi | Standard |
| 5 | Benzoylhypaconitine | C_31_H_43_NO_9_ | 5.75 | [M+H]^+^ | 574.3076 | 6.09 | 542.2789, 510.2120, 105.0314 | FuZi | Liu et al., 2019 |
| 6 | Coumarin | C_9_H_6_O_2_ | 5.86 | [M+H]^+^ | 147.0478 | 5.44 | 103.0540, 91.0549 | Cinnamon | Standard |
| 7 | Mesaconitine | C_33_H_45_NO_11_ | 6.07 | [M+H]^+^ | 632.2971 | -8.54 | 572.2377，540.2372，512.2950，105.0269 | FuZi | Massbank |
| 8 | Cinnamic alcohol | C_9_H_10_O | 6.14 | [M-H_2_O+H]^+^ | 117.0677 | -9.39 | 115.0544, 105.0696,91.0539,77.0350 | Cinnamon | Su et al., 2013 |
| 9 | Cinnamic acid | C_9_H_8_O_2_ | 6.25 | [M+H]^+^ | 149.0560 | 4.03 | 147.0460, 131.0501，103.0501，91.0534，77.0380 | Cinnamon | Standard |
| 10 | Hypaconitine | C_33_H_45_NO_10_ | 6.72 | [M+H]^+^ | 616.3144 | 4.54 | 556.2562，524.2822，338.2043, 105.0358 | FuZi | Massbank |
| 11 | Cinnamaldehyde | C_9_H_8_O | 12.05 | [M+H]+ | 133.0655 | 5.26 | 105.0701,91.0548,79.0549,77.0391,55.0545 | Cinnamon | Standard/Massbank |


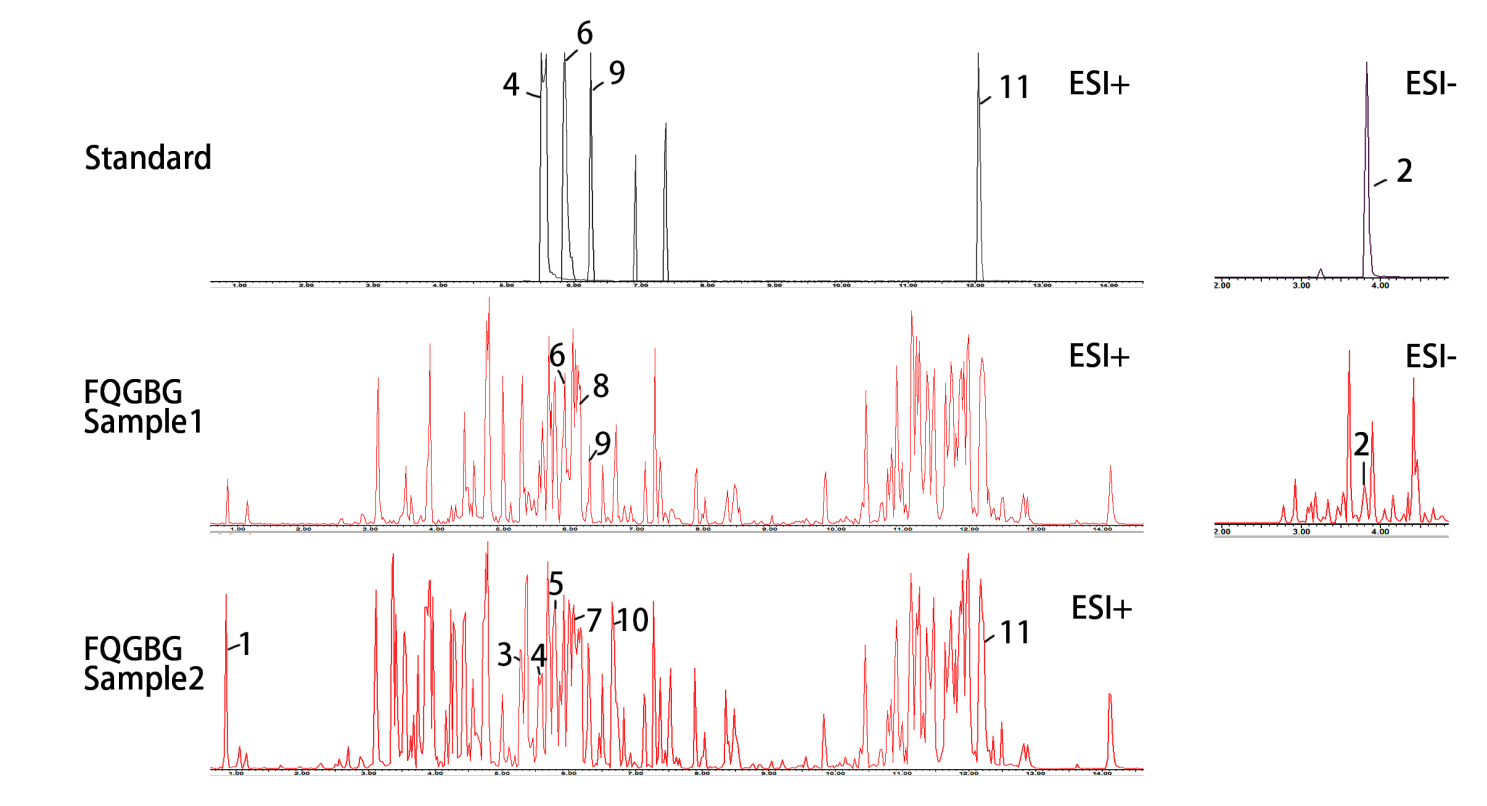


Fig. S8. UPLC-QTOF-MS chromatograms of FQGBG. Peaks: Betaine (1), Caffeic acid (2), Benzoylmesaconitine (3), Benzoylaconitine (4), Benzoylhypaconitine (5), coumarin (6), Mesaconitine (7), Cinnamic alcohol (8), Cinnamic acid (9), Hypaconitine (10), cinnamaldehyde (11). Sample 1 was obtained by acidifying the sample and adding ethyl acetate for liquid-liquid extraction. Sample 2 was obtained by alkalizing the sample and adding ethyl acetate for liquid-liquid extraction.

**_
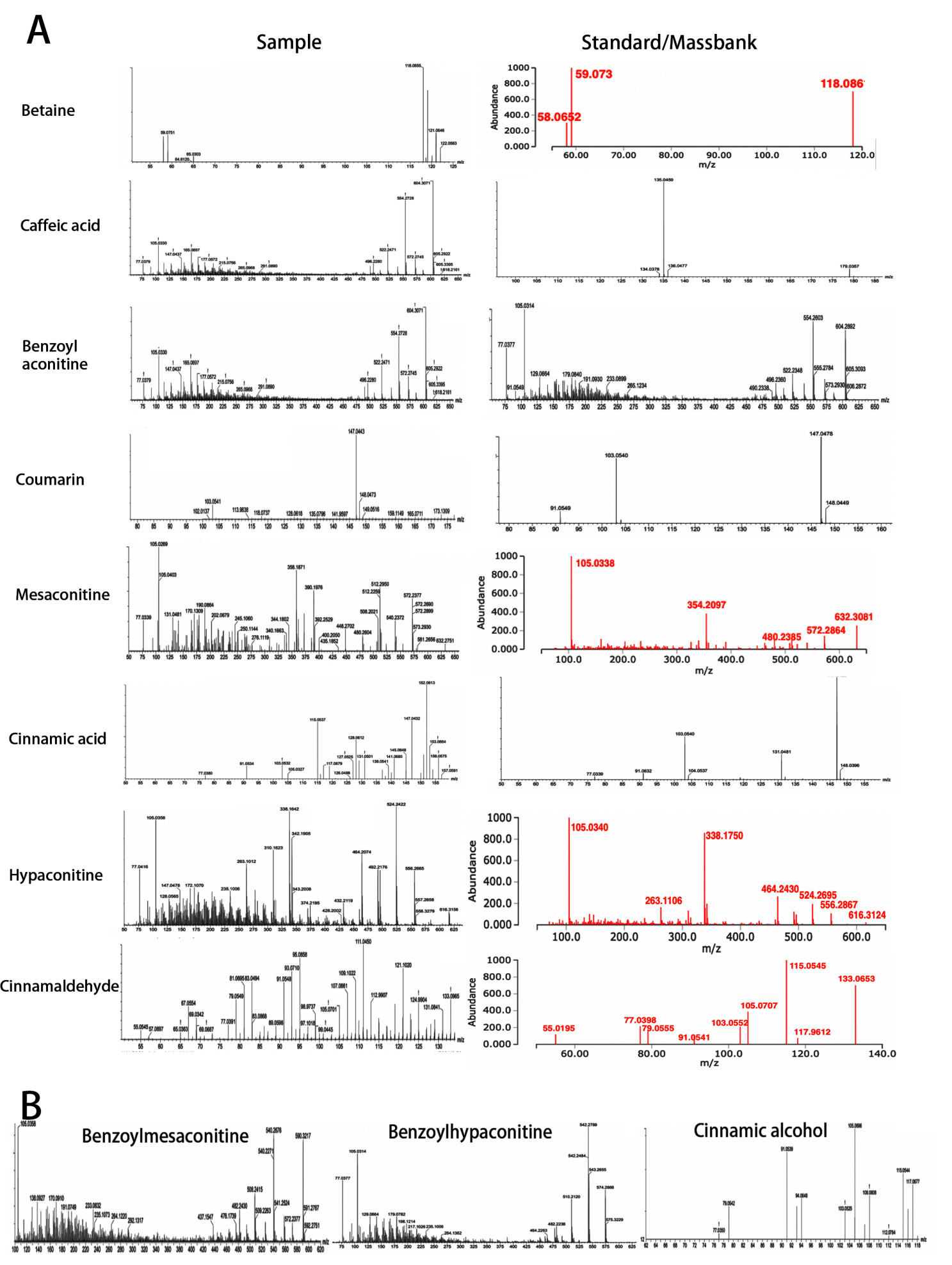
_**

Fig. S9. MS/MS spectra of components in FQGBG. (A) Components identified by standard sample or Massbank database. (B) Components identified with reference.

**1.1.2 Thin layer chromatography (TLC) fingerprinting of FQGBG**

Take 5g of FQGBG, dilute with 10mL of water, adjust the pH to 9~10 with aqueous ammonia, and perform a two-phase extraction using an ether-chloroform mixture (3:1 v/v), 20mL each time. Combine the organic phases, and remove the solvent under vacuum at 40°C until dry. Redissolve the residue in 1mL of an isopropanol-dichloromethane mixture (1:1 v/v), and spot onto a silica gel G thin-layer chromatography (TLC) plate. Develop the chromatogram using a mobile phase of n-hexane:ethyl acetate:methanol (4:2:1 v/v/v) pre-saturated with ammonia vapor for 20 minutes. After development, air-dry the plate and visualize the compounds by spraying with a dilute solution of bismuth potassium iodide. This procedure is employed to identify Fuzi metabolites in FQGBG. In the analysis for Wolfberry metabolites, take 10g of FQGBG, dissolve in 20mL of water, centrifuge, and transfer the supernatant to a separatory funnel. Extract twice with ethyl acetate (30mL, then 25mL), combine the ethyl acetate extracts, and evaporate the solvent under reduced pressure until dry. Dissolve the dry residue in 1mL of ethyl acetate, and spot onto a silica gel G TLC plate. Develop the plate with a solvent system of toluene:ethyl acetate:formic acid (7:3:2 v/v/v). Air-dry the plate and examine the chromatogram under UV light at 365nm. For identification of Cinnamon metabolites, dissolve 5g of the sample in 25mL water, and extract three times with petroleum ether (60–90°C) (30mL, 25mL, and 25mL). Pool the petroleum ether extracts, concentrate under vacuum until dry, and redissolve the residue in 1mL of petroleum ether (60–90°C). Spot the solution onto a silica gel G TLC plate, and develop using a solvent system of petroleum ether (60–90°C):ethyl acetate (5:1 v/v). After development, air-dry the plate, then spray with an anisaldehyde-sulfuric acid reagent, and heat at 105°C to enable spot visualization. The results were depicted in Fig. S10.


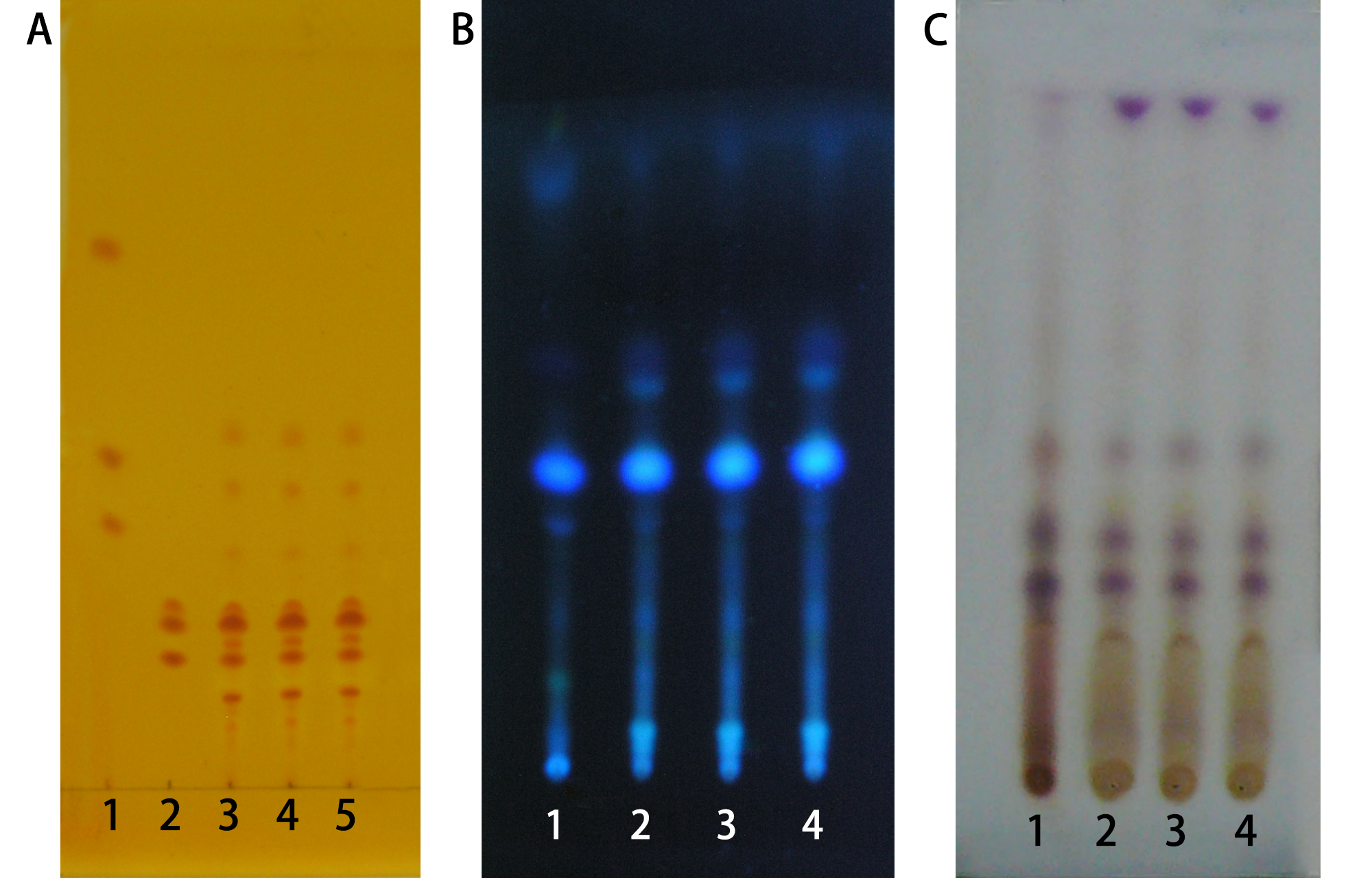


Fig. S10 TLC Profiles of FQGBG( A: Identification of Fuzi metabolites in FQGBG, Lane 1: Diester-diterpenoid alkaloids reference (top to bottom: HA, aconitine (AC), MA), Lane 2: Monoester-diterpenoid alkaloids reference (top to bottom: BHA, benzoylaconine (BAC), BMA), Lanes 3-5: FQGBG samples; B: Identification of Wolfberry metabolites in FQGBG, Lane 1: Wolfberry herbal reference, Lanes 2-4: FQGBG samples; C: Identification of Cinnamon metabolites in FQGBG, Lane 1: Cinnamon herbal reference, Lanes 2-4: FQGBG samples)

**1.1.3 HPLC-UV fingerprinting of FQGBG and Quality control**

The contents of diester alkaloids (AC, HA, MA) and monoester alkaloids (BAC, BHA, BMA) in FQGBG were determined by the HPLC-UV method (Chinese Pharmacopoeia, 2010, Appendix VI D) to control the quality of FQGBG. The total content of AC, HA, and MA was required not to exceed 1μg per 1g, and the total amount of BAC, BHA, and BMA should not be less than 80 μg per 1g in this drug, respectively. In addition, The drug was semisolid thick brown, aromatic, sweet taste. The relative densities were in the range of 1.3 to 1.4. The total number of Aerobic Bacteria, Moulds and Yeasts did not exceed 10^3^ cfu/g, and the Escherichia coli must not be detected.

The C18 chromatographic column was used for sample analysis. Acetonitrile: tetrahydrofuran (25:15,v/v) was used as mobile phase A, and the 0.1mol/L ammonium acetate solution was used as mobile phase B. The detection wavelength was set at 235 nm. The gradient elution program for AC, MA, and HA was as follows: 0–15 min, 15→18% A, 15–55 min, 18% A, The flow rate was 0.8 mL/min, and the injection volume was 10 μL. The gradient elution program for BAC, BMA, and BHA was as follows: 0 min, 12% A, 0-40 min, 12→15% A, The flow rate was 1.0 mL/min, and the injection volume was 10 μL. An external standardization determined the content of six compounds. The calibration curve for AC was 0.199–9.95 µg, for HA was 0.1804–9.020 µg, for MA was 0.2018–10.09 µg, for BAC was 0.0425–1.70 µg, for BHA was 0.04420–1.768 µg, and for BMA was 0.045–1.8 µg. Fingerprintings were shown in Fig. S11 (A, B).

Identification of Wolfberry metabolites——betaine in FQGBG using a NH_2_-bonded silica chromatographic column, with acetonitrile-water (88:12) as the mobile phase for isocratic elution. The detection wavelength was set at 195 nm with a flow rate of 0.8 mL/min. Results are shown in Fig. S11 (C).


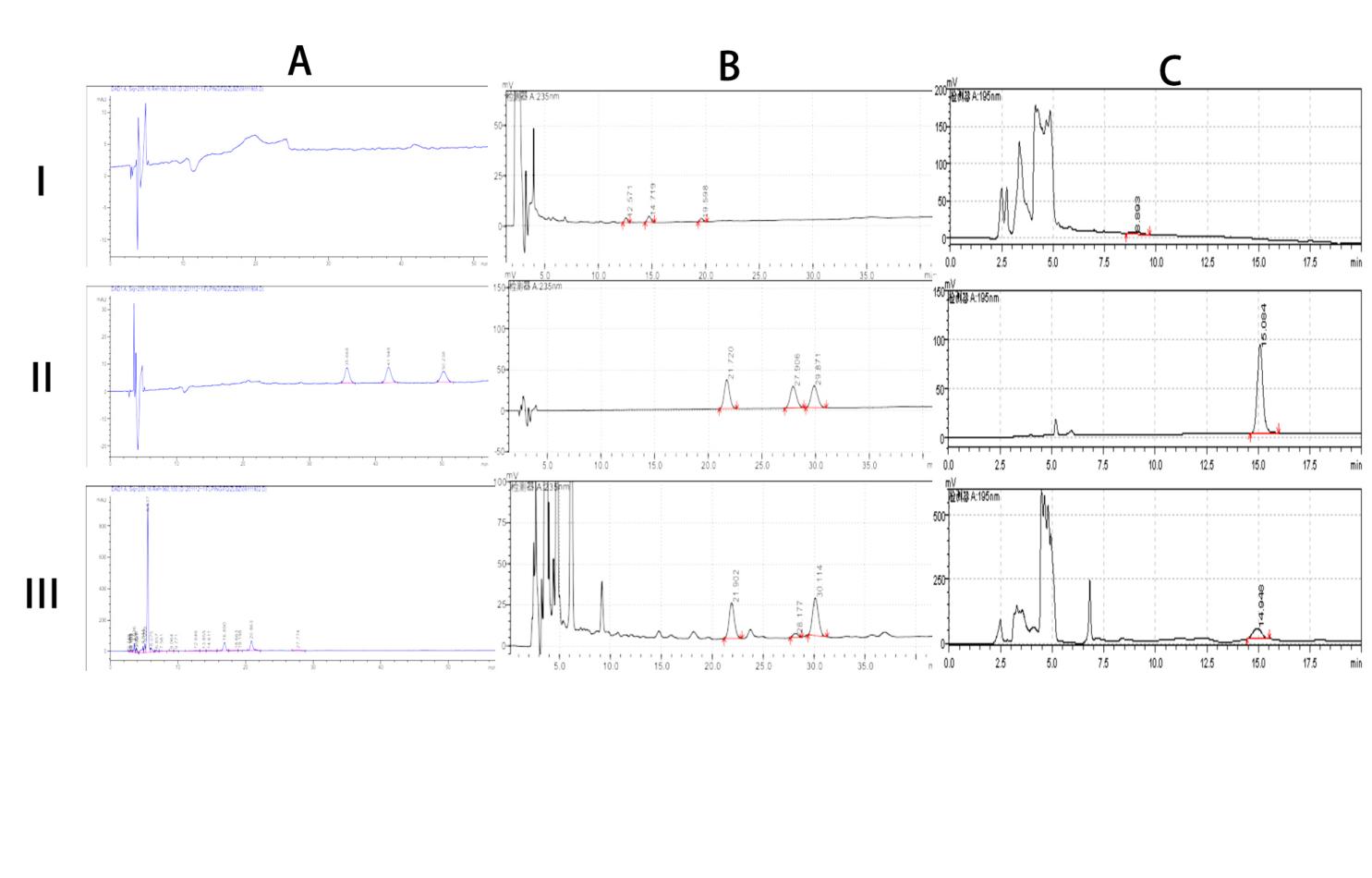


Fig. S11: FQGBG HPLC-UV Chromatograms.(A: HPLC-UV profile for AC, MA, HA; B: HPLC-UV profile for BAC, BMA, BHA; C: HPLC-UV profile for Betaine; I represents the blank control solution; II represents the reference standard solution; III represents the FQGBG test sample solution.)

**1.2 Exclusion criteria**

Those meeting the following criteria were excluded: (1) symptoms of fever, afraid of heat, thirst, red face, restlessness, phlegm and thick yellow nasal mucous, decreased yellow urine, dry stool, red and dry tongue fluid, yellow-coated tongue, rapid pulse, and other heat syndrome manifestations; (2) smokers who smoked more than 10 cigarettes/day or individuals with high alcohol consumption (>35 g/day); (3) drug abuse; (4) blood donation or loss of more than 400 mL within the past 3 months; (5) pregnant, breast-feeding, or menstruating, as well as planned pregnancy within 3 months; (6) history of central nervous, cardiovascular, respiratory, digestive, circulatory, urogenital, blood, metabolic and endocrine, or motor system diseases that may have implications on the clinical trial as per the investigator’s judgment; (7) use of any food or drugs that affected the drug metabolism enzyme activity within 14 days; (8) participation in a clinical trial in the past 3 months; (9) use of drugs known to cause damage to a certain organ in the past 3 months; (10) drug allergy history or allergic constitution; (11) positive breath alcohol test and infectious disease screening results; and (12) poor compliance or unsuitability for the clinical trial as per the investigator’s judgment.

**1.3 Preparation of standard solutions and QC sample preparation for PK analysis**

In order to prepare the primary stock solutions, aconitine (AC) was accurately weighed and dissolved in a hydrochloric methanol solution, mesaconitine (MA) and hypaconitine (HA) were dissolved in methanol, whereas benzoylaconitine (BAC) benzoylmesaconine (BMA), and benzoylhypacoitine (BHA) were dissolved in DMSO. The stock solutions were mixed and serially diluted with ACN: DMSO: H2O (60: 20: 20, v/v/v) to obtain standard working and quality control (QC) solutions. The nor verapamil hydrochloride (Internal standard, IS) stock solution was prepared in DMSO and diluted with ACN: H2O (50: 50, v/v) to obtain a working solution at concentrations of 10 ng/mL (for analysis of BAC, BMA, and BHA) and 50 ng/mL(for analysis of AC, MA, and HA). All solutions were stored at 4°C.

The calibration standards and QC samples were prepared by diluting the working solution 20 times with blank plasma. 100 μL samples, including calibration standards and QC samples, spiked with 20 μL of IS were mixed evenly on a shaker. Then, 500 μL ACN containing 0.1% formic acid was added to the sample. Protein precipitation was performed by vortexing the mixture for 10 min and then centrifuged at 4000 rpm for 10 min at 4° C. 150 μL supernatant was added into equal volume water containing 0.1% formic acid. The mixture was analyzed by UPLC-MS/MS method.

**1.4 Methodological evaluation**

**1.4.1 Specificity**

The method for this study was fully validated according to the USA Food and Drug Administration (FDA) bio-analytical method validation guidance (CDER., 2011). As shown in Fig. S12-S13, the baseline of AC, MA, HA, BAC, BMA, BHA, and IS was separated under the selected chromatographic conditions. There was no interference from endogenous compounds at the retention times of the analytes.

**
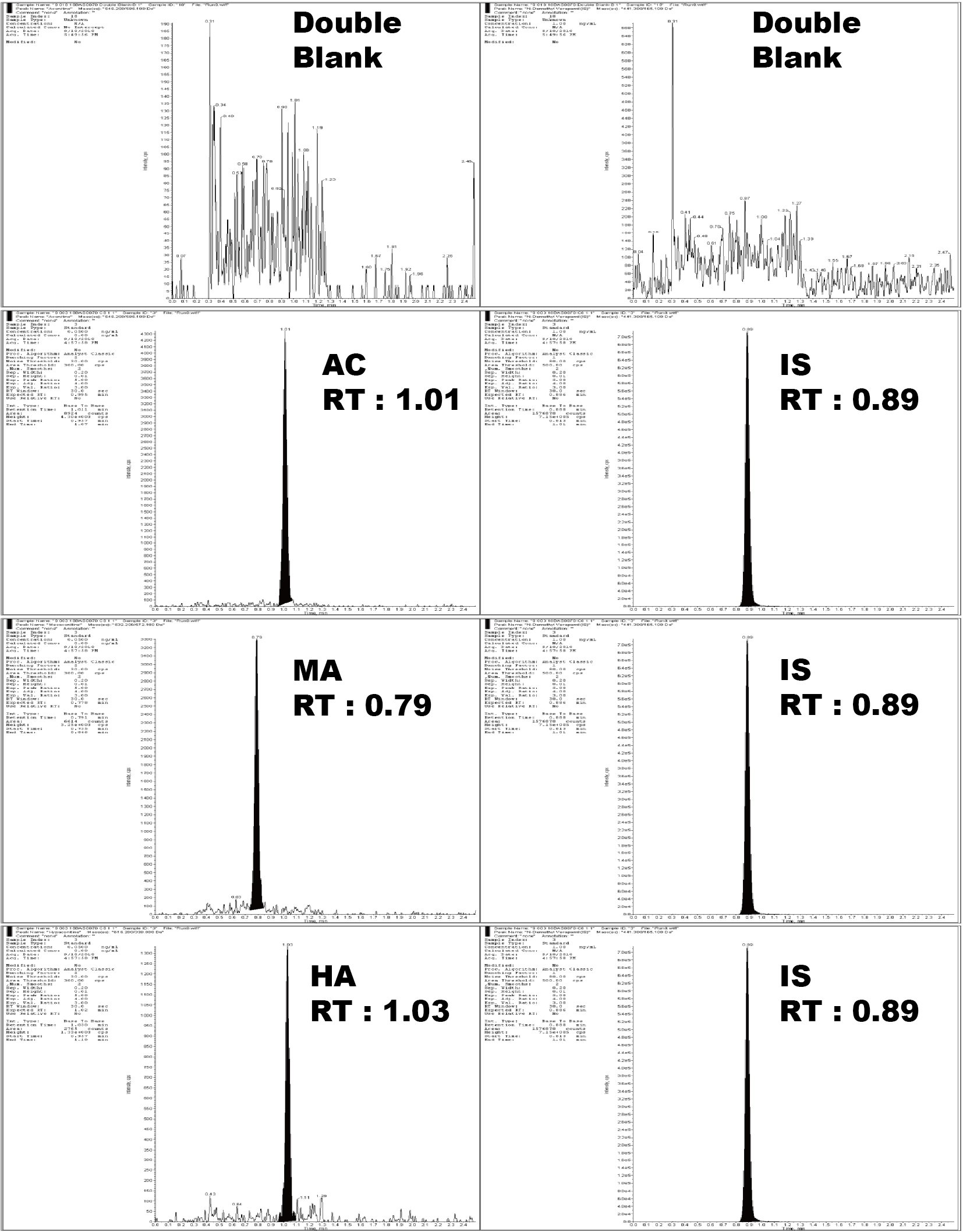
**

Fig. S12. Representative MRM chromatograms of AC, MA, HA, and IS in double blank plasma and Lower limit of quantitation (LLOQ) sample.


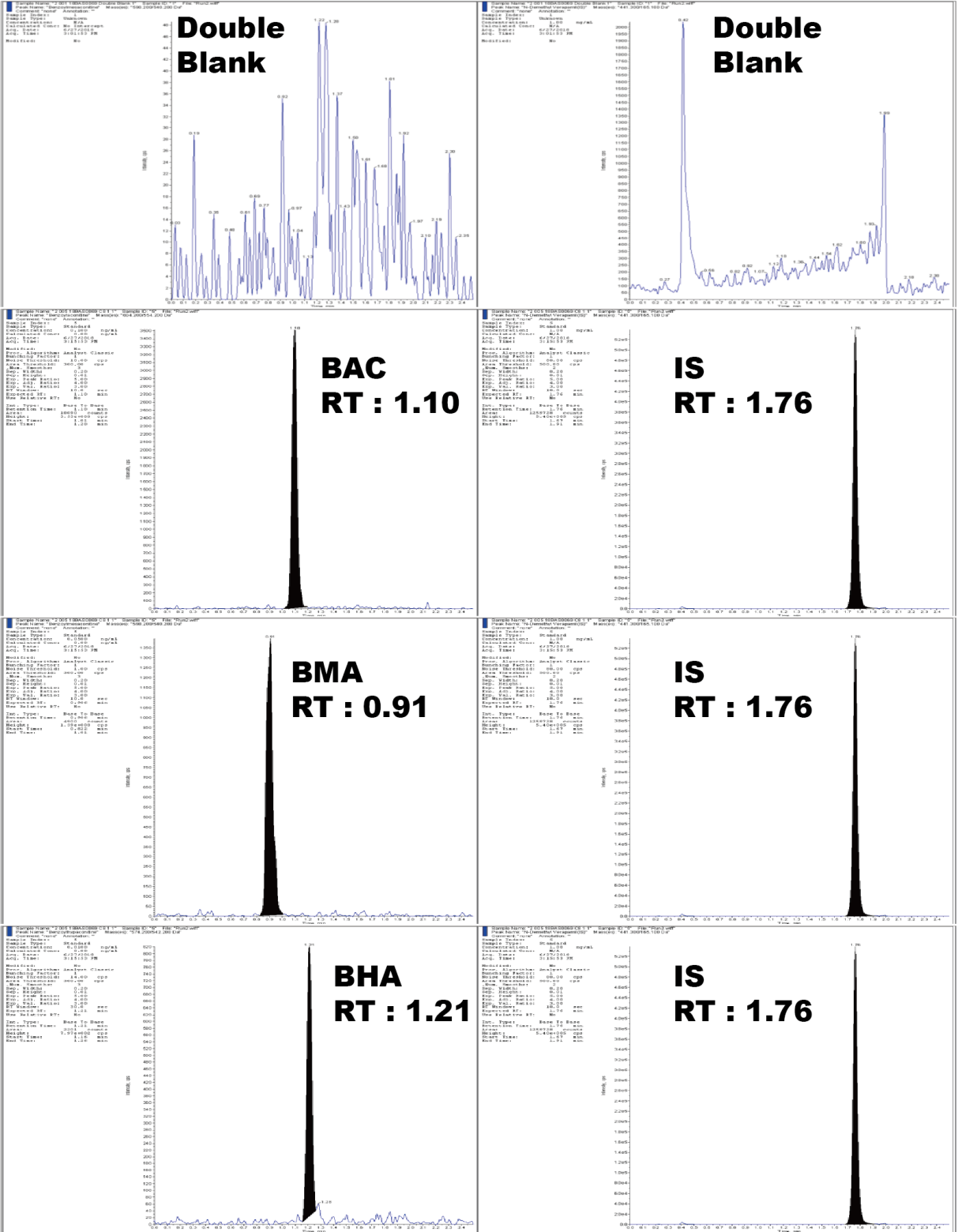


Fig. S13. Representative MRM chromatograms of BAC, BMA, BHA, and IS in double blank plasma and LLOQ sample.

**1.4.2 Calibration curve linearity and Sensitivity**

The Calibration standard curves for AC, MA, HA, and BMA ranged from 0.05 to 50 ng/mL, for BAC from 0.1 to 100 ng/mL, and for BHA from 0.01 to 10ng/mL. As shown in Table S5, the calibration curves of all analytes showed good linearity (r^2^ > 0.99).

Table S5. Calibration curves and correlation coefficient

| Components | Equation | Correlation coefficient (r) | LLOQ (ng/mL) |
| --- | --- | --- | --- |
| AC | Y=0.08068X+0.0002457 | 0.9979 | 0.050 |
| MA | Y=0.06549X+0.0002174 | 0.9977 | 0.050 |
| HA | Y=0.2378 X+0.0001426 | 0.9975 | 0.050 |
| BAC | Y=0.09938X+0.0005401 | 0.9975 | 0.100 |
| BMA | Y=0.09569X+0.0001764 | 0.9968 | 0.050 |
| BHA | Y=0.2365 X+0.0001471 | 0.9972 | 0.010 |

**1.4.3 Accuracy and precision**

The intra-day (n = 6) and inter-day (n = 6) accuracies and precisions were evaluated by testing four concentrations of samples, including LLOQ and QC samples. As shown in Table S6, the absolute value of RSD was not higher than 15%, and the accuracy rates were within 14%. The results indicated that the precision and accuracy met the acceptability criteria for biological sample analysis.

Table S6. Accuracy and precision

| Nominal Conc. (ng/mL) | Observed Conc. (ng/mL) | Accuracy  (RE%) | Intra-day precision (RSD%) | Inter-day precision (RSD%) |
| --- | --- | --- | --- | --- |
| AC |  |  |  |  |
| 0.050 | 0.0549±0.00140 | 9.8 | 2.6 | 4.5 |
| 0.15 | 0.157±0.00349 | 4.7 | 2.2 | 2.7 |
| 25 | 23.7±0.223 | -5.2 | 0.9 | 3.7 |
| 40 | 38.2±0.458 | -4.5 | 1.2 | 1.9 |
| MA |  |  |  |  |
| 0.050 | 0.0540±0.000905 | 8.0 | 1.7 | 3.9 |
| 0.15 | 0.158±0.00214 | 5.3 | 1.4 | 3.3 |
| 25 | 23.6±0.190 | -5.6 | 0.8 | 3.4 |
| 40 | 38.0±0.426 | -5.0 | 1.1 | 1.9 |
| HA |  |  |  |  |
| 0.050 | 0.0514±0.00320 | 2.8 | 6.2 | 8.3 |
| 0.15 | 0.159±0.00313 | 4.0 | 2.0 | 4.0 |
| 25 | 23.8±0.186 | -4.8 | 0.8 | 3.6 |
| 40 | 38.5±0.314 | -3.8 | 0.8 | 1.8 |
| BAC |  |  |  |  |
| 0.10 | 0.0896±0.00515 | -10.4 | 5.7 | 8.0 |
| 0.30 | 0.296±0.0071 | -1.3 | 2.6 | 5.8 |
| 50 | 53.2±3.54 | 6.4 | 6.7 | 5.4 |
| 80 | 84.7±5.65 | 5.9 | 6.7 | 6.0 |
| BMA |  |  |  |  |
| 0.050 | 0.0452±0.00236 | -9.6 | 5.2 | 6.6 |
| 0.15 | 0.158±0.00480 | 5.3 | 3.0 | 5.6 |
| 25 | 28.5±1.89 | 14.0 | 6.6 | 5.5 |
| 40 | 45.4±3.00 | 13.5 | 6.6 | 6.7 |
| BHA |  |  |  |  |
| 0.010 | 0.0104±0.00179 | 4.0 | 17.2 | 14.5 |
| 0.030 | 0.0301±0.00122 | 4.1 | 0.3 | 7.0 |
| 5.0 | 5.25±0.349 | 7.0 | 6.5 | 5.4 |
| 8.0 | 8.59±0.580 | 7.4 | 6.8 | 5.9 |

RSD% = (standard deviation/mean) × 100%. RE% = (mean of observed Conc. nominal Conc.)/nominal Conc. ×100%.

**1.4.4 Extraction recovery and matrix effect**

The extraction recovery was calculated by comparing the signal of each compound and internal standard in blank plasma samples before extraction with that of samples to which each compound and internal standard had been added post-extraction at the same concentration. As shown in Table S7, the extraction recovery of all compounds was between 85.4% and 102.0% at all the three concentrations. The extraction recovery data indicated that the extraction procedure was efficient for AC, MA, HA, BAC, BMA, and BHA at three QC levels. The matrix effects (ME) were calculated by comparing the signals of compounds and internal markers added post-extraction in blank plasma with those in blank solvents. After normalization of the ME data with the IS, the ME values of each analyte were reduced, which was related to the matrix's enhancement of the IS signal. All compounds had almost no matrix effect for LQC and HQC.

Table S7. Extraction recovery and matrix effect

| Nominal Conc. (ng/mL) | Extraction recovery  (mean ± SD%) | RSD%  (Extraction recovery) | IS-normalized ME (mean ± SD%) | RSD% (IS-normalized ME) |
| --- | --- | --- | --- | --- |
| AC |  |  |  |  |
| 0.15 | 91.4±2.14 | 2.3 | 0.92±0.05 | 5.1 |
| 25 | 99.0±7.25 | 7.3 | - | - |
| 40 | 100.4±5.63 | 5.6 | 0.93±0.04 | 3.9 |
| MA |  |  |  |  |
| 0.15 | 89.7±1.25 | 1.4 | 0.91±0.03 | 3.4 |
| 25 | 98.1±6.36 | 6.5 | - | - |
| 40 | 99.7±4.91 | 4.9 | 0.94±0.04 | 3.7 |
| HA |  |  |  |  |
| 0.15 | 94.7±3.37 | 3.6 | 0.96±0.06 | 6.1 |
| 25 | 99.9±6.52 | 6.5 | - | - |
| 40 | 102.0±5.08 | 5.0 | 0.93±0.04 | 4.5 |
| BAC |  |  |  |  |
| 0.30 | 85.4±6.67 | 7.8 | 0.99±0.03 | 2.5 |
| 50 | 88.5±7.06 | 8.0 | - | - |
| 80 | 90.9±6.34 | 7.0 | 0.99±0.01 | 1.0 |
| BMA |  |  |  |  |
| 0.15 | 85.7±8.64 | 10.1 | 0.99±0.04 | 4.3 |
| 25 | 87.5±6.96 | 8.0 | - | - |
| 40 | 88.8±5.89 | 6.6 | 0.97±0.01 | 0.5 |
| BHA |  |  |  |  |
| 0.030 | 89.4±8.21 | 9.2 | 1.03±0.10 | 9.8 |
| 5.0 | 89.1±7.22 | 8.1 | - | - |
| 8.0 | 90.9±5.53 | 6.1 | 0.98±0.01 | 1.4 |

**1.4.5 Stability**

The stability was determined at two concentrations in plasma 24 h storage in the ice, in autosampler after preparation at 6℃for 95 h, and 62-days storage at −80 °C. As shown in Table S8, the accuracy of all compounds under all storage conditions was between 85% and 115%. Results suggested that plasma samples were stable during our experimental period.

Table S8. Stability

| Nominal Conc. (ng/mL) | Accuracy% | | |
| --- | --- | --- | --- |
|  | Storage in the ice for 24 h | Storage in autosampler at 6℃ | Storage at-80℃ for 62 days |
| AC |  |  |  |
| 0.15 | 96.7 | 100.7 | 98.7 |
| 40 | 91.5 | 97.2 | 98.0 |
| MA |  |  |  |
| 0.15 | 95.3 | 100.7 | 92.7 |
| 40 | 91.0 | 96.8 | 90.5 |
| HA |  |  |  |
| 0.15 | 96.7 | 104.7 | 96.0 |
| 40 | 95.7 | 97.7 | 94.5 |
| BAC |  |  |  |
| 0.30 | 103.0 | 110.3 | 107.3 |
| 80 | 97.2 | 104.9 | 100.1 |
| BMA |  |  |  |
| 0.15 | 104.7 | 107.3 | 106.0 |
| 40 | 99.0 | 105.5 | 102.0 |
| BHA |  |  |  |
| 0.030 | 104.0 | 105.3 | 108.3 |
| 8.0 | 100.6 | 106.5 | 103.1 |

**1.5 Metabonomics sample preparation**

300 μL methanol was added into 100 μL plasma to scavenge protein. Then, the samples were vortexed in a vortexer for 15 sec and centrifuged at 12000 r/min at 4 ℃ for 10 min. The resultant supernatant was collected and evaporated to dryness by nitrogen flow at room temperature. The residual was dissolved and vortexed in 100 μL ACN: Water (50:50, v/v) and centrifuged at 12000 r/min at 4 ℃ for 10 min. 100 μL urine was mixed with 100 μL acetonitrile. After vortexed for 30 sec, the sample was centrifuged at 12000 r/min at 4 ℃ for 10 min. 2 μL of the supernatant of each sample was analyzed by UPLC-QTOF-MS.

Before analysis, 5 μL of each sample was pipetted into a centrifuge tube to prepare quality control (QC) samples to investigate instrument precision, method repeatability, and sample stability.

**1.6 Chromatographic and Mass Spectrometric Conditions**

Metabonomics research was performed using a Waters Acquity ^TM^ UPLC combined with Waters Synapt G2-Si High Definition Mass Spectrometer (HDMS) system (Waters, Milford, MA, USA). The MassLynx^TM^ 4.1 workstation was utilized for following data acquisition and processing.

Plasma and urine samples were injected into an Acquity UPLC HSS T3 column (2.1 mm × 100 mm, 1.8 μm, Waters, Milford, MA, USA). The column temperature was set at 45 ℃, and the flow rate was 0.3 mL/min. The mobile phase consisted of solvent A (0.1% formic acid in water) and solvent B (0.1% formic acid in acetonitrile). The gradient for plasma was set as follows: 0−1 min, 95% A; 1−12 min, 95-0% A; 12−15 min, 0% A; 15−15.1 min, 95% A; 15.1-16 min, 95% A. The gradient for urine was set as follows: 0−1 min, 99% A; 1−4 min, 99-85% A; 4−9 min, 85-50% A; 9−13 min, 50-5% A; 13−15.1 min, 5% A; 15−15.1 min, 5-99% A; 15.1-16 min, 99% A.

The MS data were acquired in the positive and negative ion modes using a data-independent acquisition approach (MS^E^). An electrospray ionization source (ESI source) parameters were as follows: a capillary voltage of 3.0 kV for positive mode, of -2.5 kV for negative mode; cone voltage of 30 V; desolvation temperature of 400 ℃ for plasma sample, of 550 ℃ for urine sample; desolvation gas flow of 800 L/h; source temperature of 120 ℃; ion scan, *m/z* 50-1500 Da for the plasma sample*, m/z* 50-1200 Da for the urine sample.

**Reference**

Liu H, Qiu F, Yang X, Zhao H, Bian B, Wang L. Pharmacokinetics of the Yougui pill in experimental autoimmune encephalomyelitis model rats and its pharmacological activity in vitro. Drug Des Devel Ther. 2019 Jul 16;13:2357-2370. https://doi.org/10.2147/DDDT.S203874.

Su S, Cui W, Zhou W, Duan JA, Shang E, Tang Y. Chemical fingerprinting and quantitative constituent analysis of Siwu decoction categorized formulae by UPLC-QTOF/MS/MS and HPLC-UV-DAD. Chin Med. 2013 Mar 1;8(1):5. [https://doi.org/10.1186/1749-8546-8-5](https://doi.org/10.1002/bmc.3102).

C. P. Commission, Pharmacopoeia of the People’s Republic of China, China Medical Science Press, Beijing, China, 2010.

1. **Supplementary Material S2**

**2.1 Supplementary Tables**

Table S1. Safety analysis of TEAE by system

| SOC  PT | 12.5 g | | 25 g | | 50 g | | 75 g | | 100 g | | 125 g | | placebo | |
| --- | --- | --- | --- | --- | --- | --- | --- | --- | --- | --- | --- | --- | --- | --- |
|  | Number of cases | cases | Number of cases | cases | Number of cases | cases | Number of cases | cases | Number of cases | cases | Number of cases | cases | Number of cases | cases |
| **n** | 2 |  | 6 |  | 6 |  | 6 |  | 6 |  | 6 |  | 10 |  |
| **Sum n (%)** | 1(50.0) | 2 | 2(33.3) | 2 | 2(33.3) | 3 | 5(83.3) | 8 | 3(50.0) | 4 | 5(83.3) | 12 | 5(50.0) | 8 |
| **Investigations n (%)** | 0(0) | 0 | 1(16.7) | 1 | 1(16.7) | 1 | 2(33.3) | 4 | 2(33.3) | 3 | 2(33.3) | 3 | 3(30.0) | 6 |
| Elevated NAG enzyme n (%) | 0(0) | 0 | 0(0) | 0 | 0(0) | 0 | 2(33.3) | 2 | 0(0) | 0 | 1(16.7) | 1 | 0(0) | 0 |
| Positive urine occult blood n (%) | 0(0) | 0 | 0(0) | 0 | 1(16.7) | 1 | 0(0) | 0 | 0(0) | 0 | 0(0) | 0 | 2(20.0) | 2 |
| Elevated blood uric acid n (%) | 0(0) | 0 | 0(0) | 0 | 0(0) | 0 | 0(0) | 0 | 0(0) | 0 | 1(16.7) | 1 | 1(10.0) | 1 |
| Low leucocyte count n (%) | 0(0) | 0 | 0(0) | 0 | 0(0) | 0 | 0(0) | 0 | 1(16.7) | 1 | 0(0) | 0 | 0(0) | 0 |
| Elevated leucocyte count n (%) | 0(0) | 0 | 1(16.7) | 1 | 0(0) | 0 | 0(0) | 0 | 0(0) | 0 | 0(0) | 0 | 0(0) | 0 |
| Positive urine protein n (%) | 0(0) | 0 | 0(0) | 0 | 0(0) | 0 | 2(33.3) | 1 | 0(0) | 0 | 0(0) | 0 | 0(0) | 0 |
| Abnormal ECG t wave n (%) | 0(0) | 0 | 0(0) | 0 | 0(0) | 0 | 0(0) | 0 | 1(16.7) | 1 | 0(0) | 0 | 0(0) | 0 |
| ECG high voltage n (%) | 0(0) | 0 | 0(0) | 0 | 0(0) | 0 | 0(0) | 0 | 0(0) | 0 | 0(0) | 0 | 1(10.0) | 3 |
| Elevated serum creatinine n (%) | 0(0) | 0 | 0(0) | 0 | 0(0) | 0 | 0(0) | 0 | 0(0) | 0 | 1(16.7) | 1 | 0(0) | 0 |
| Low neutrophil count n (%) | 0(0) | 0 | 0(0) | 0 | 0(0) | 0 | 0(0) | 0 | 1(16.7) | 1 | 0(0) | 0 | 0(0) | 0 |
| **Gastrointestinal disorder n (%)** | 1(50.0) | 1 | 0(0) | 0 | 1(16.7) | 1 | 1(16.7) | 1 | 1(16.7) | 1 | 2(33.3) | 5 | 2(20.0) | 2 |
| Thirst n (%) | 0(0) | 0 | 0(0) | 0 | 1(16.7) | 1 | 1(16.7) | 1 | 0(0) | 0 | 1(16.7) | 1 | 2(20.0) | 2 |
| Diarrhea n (%) | 0(0) | 0 | 0(0) | 0 | 0(0) | 0 | 0(0) | 0 | 0(0) | 0 | 2(33.3) | 2 | 0(0) | 0 |
| Abdominal distension n (%) | 0(0) | 0 | 0(0) | 0 | 0(0) | 0 | 0(0) | 0 | 0(0) | 0 | 1(16.7) | 1 | 0(0) | 0 |
| Oral microsites n (%) | 0(0) | 0 | 0(0) | 0 | 0(0) | 0 | 0(0) | 0 | 0(0) | 0 | 1(16.7) | 1 | 0(0) | 0 |
| Toothache n (%) | 1(50.0) | 1 | 0(0) | 0 | 0(0) | 0 | 0(0) | 0 | 0(0) | 0 | 0(0) | 0 | 0(0) | 0 |
| Gum pain n (%) | 0(0) | 0 | 0(0) | 0 | 0(0) | 0 | 0(0) | 0 | 1(16.7) | 1 | 0(0) | 0 | 0(0) | 0 |
| **General disorders and administration site conditions n (%)** | 0(0) | 0 | 0(0) | 0 | 0(0) | 0 | 1(16.7) | 1 | 0(0) | 0 | 4(66.7) | 4 | 0(0) | 0 |
| Heat sensation n (%) | 0(0) | 0 | 0(0) | 0 | 0(0) | 0 | 1(16.7) | 1 | 0(0) | 0 | 4(66.7) | 4 | 0(0) | 0 |
| **Metabolism and nutrition disorders n (%)** | 0(0) | 0 | 0(0) | 0 | 1(16.7) | 1 | 1(16.7) | 1 | 0(0) | 0 | 0(0) | 0 | 0(0) | 0 |
| Dyslipidemia n (%) | 0(0) | 0 | 0(0) | 0 | 1(16.7) | 1 | 1(16.7) | 1 | 0(0) | 0 | 0(0) | 0 | 0(0) | 0 |
| **Cardiac disorders n (%)** | 1(50.0) | 1 | 1(16.7) | 1 | 0(0) | 0 | 0(0) | 0 | 0(0) | 0 | 0(0) | 0 | 0(0) | 0 |
| Ventricular extra systole n (%) | 1(50.0) | 1 | 1(16.7) | 1 | 0(0) | 0 | 0(0) | 0 | 0(0) | 0 | 0(0) | 0 | 0(0) | 0 |
| **Blood and lymphatic system disorders n (%)** | 0(0) | 0 | 0(0) | 0 | 0(0) | 0 | 1(16.7) | 1 | 0(0) | 0 | 0(0) | 0 | 0(0) | 0 |
| Anemia n (%) | 0(0) | 0 | 0(0) | 0 | 0(0) | 0 | 1(16.7) | 1 | 0(0) | 0 | 0(0) | 0 | 0(0) | 0 |

The AEs are coding with MedDRA (version 23.0), and SOCs and PTs are sorted by descending order of AE number.

Number of cases：One subject is only counted once in the same SOC or PT.

Cases: One subject was counted based on the actual number of occurrences in the same SOC or PT.

N and % represented the number and percentage of subjects

Table S2. The C_max_, AUC_0-t_, AUC_0-∞_ and dose relationship of BMA and BHA

| **Analyte** | **Dose** | **Parameter** | **Estimated Value** | **SE** | **95%CI** |
| --- | --- | --- | --- | --- | --- |
| BMA | 25g~100g | C_max_ |  | | |
|  |  | α | 2.889 | 0.722 | 1.392～4.386 |
|  |  | β | 0.634 | 0.178 | 0.264～1.003 |
|  |  | AUC_0-t_ |  | | |
|  |  | α | 3.366 | 1.451 | 0.357～6.375 |
|  |  | β | 1.037 | 0.359 | 0.293～1.780 |
|  |  | AUC_0-∞_ |  | | |
|  |  | α | 6.245 | 1.756 | 2.583～9.908 |
|  |  | β | 0.541 | 0.427 | -0.349～1.432 |
| BHA | 25g~100g | C_max_ |  | | |
|  |  | α | 3.018 | 0.788 | 1.384～4.653 |
|  |  | β | 0.467 | 0.195 | 0.063～0.870 |
|  |  | AUC_0-t_ |  | | |
|  |  | α | 7.501 | 0.738 | 5.971～9.032 |
|  |  | β | 0.266 | 0.182 | -0.112～0.644 |
|  |  | AUC_0-∞_ |  | | |
|  |  | α | 8.182 | 0.743 | 6.642～9.722 |
|  |  | β | 0.159 | 0.183 | -0.221～0.540 |
|  | 25g~75g | C_max_ |  |  |  |
|  |  | α | 1.693 | 0.856 | -0.121～3.508 |
|  |  | β | 0.839 | 0.223 | 0.367～1.311 |
|  |  | AUC_0-t_ |  |  |  |
|  |  | α | 5.891 | 0.666 | 4.479～7.303 |
|  |  | β | 0.719 | 0.173 | 0.352～1.086 |
|  |  | AUC_0-∞_ |  |  |  |
|  |  | α | 6.801 | 0.756 | 5.199～8.404 |
|  |  | β | 0.548 | 0.197 | 0.131～0.965 |
|  | 25g~50g | C_max_ |  |  |  |
|  |  | α | 1.775 | 0.894 | -0.216～3.766 |
|  |  | β | 0.815 | 0.249 | 0.260～1.371 |
|  |  | AUC_0-t_ |  |  |  |
|  |  | α | 4.779 | 0.917 | 2.734～6.823 |
|  |  | β | 1.046 | 0.256 | 0.475～1.616 |
|  |  | AUC_0-∞_ |  |  |  |
|  |  | α | 5.819 | 1.164 | 3.225～8.413 |
|  |  | β | 0.836 | 0.325 | 0.112～1.560 |

**2.2 Supplementary Figures**


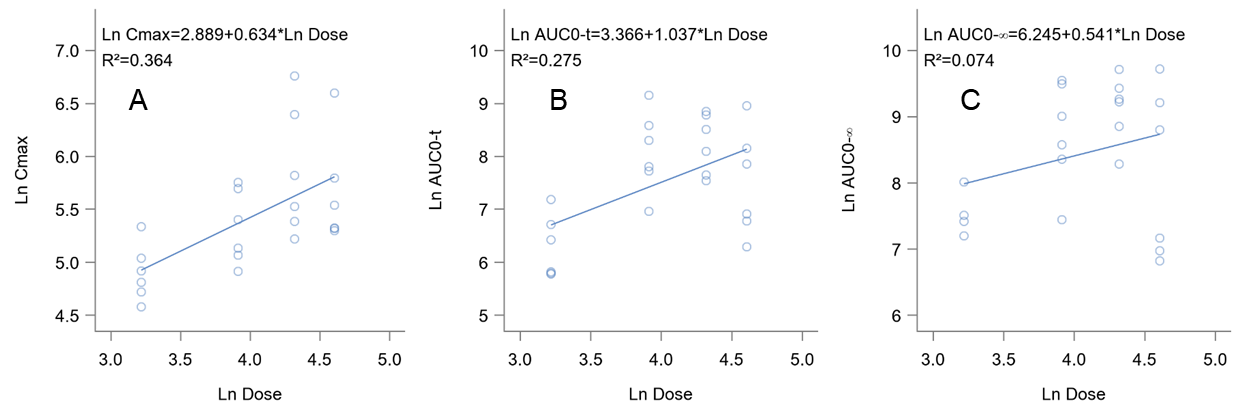


Fig. S1. The C_max_ (A), AUC_0-t_ (B), AUC_0-∞_ (C) and dose relationship of BMA (25–100 g).


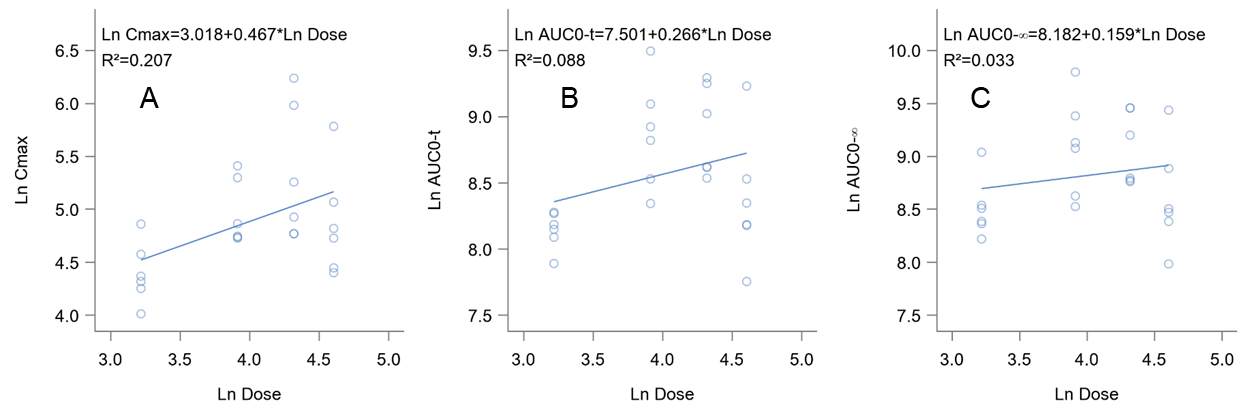


Fig. S2. The C_max_ (A), AUC_0-t_ (B), AUC_0-∞_ (C) and dose relationship of BHA (25–100 g).


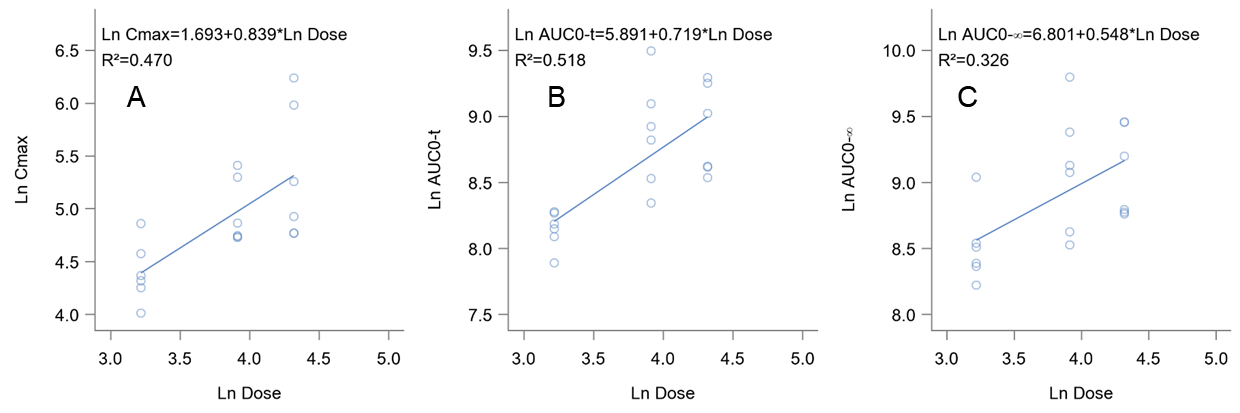


Fig. S3. The C_max_ (A), AUC_0-t_ (B), AUC_0-∞_ (C) and dose relationship of BHA (25–75 g).


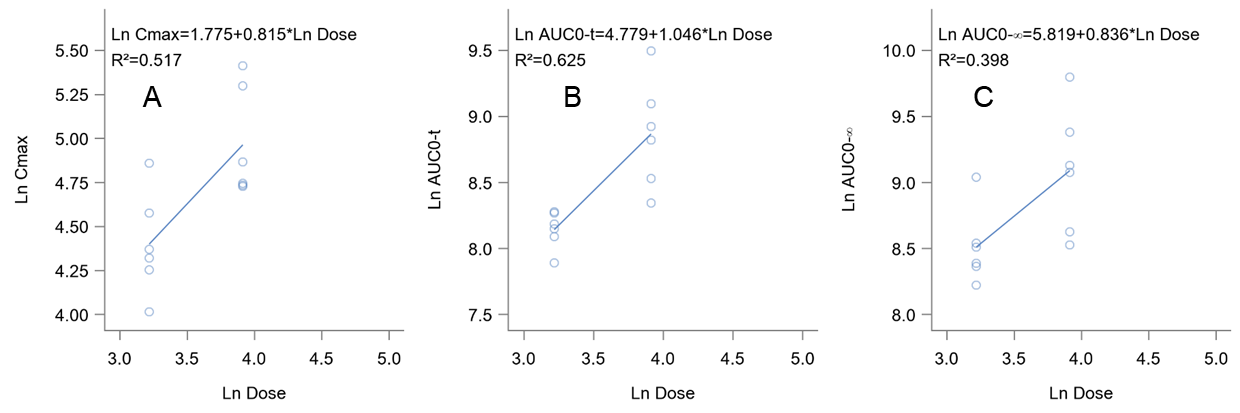


Fig. S4. The C_max_ (A), AUC_0-t_ (B), AUC_0-∞_ (C) and dose relationship of BHA (25–50 g).


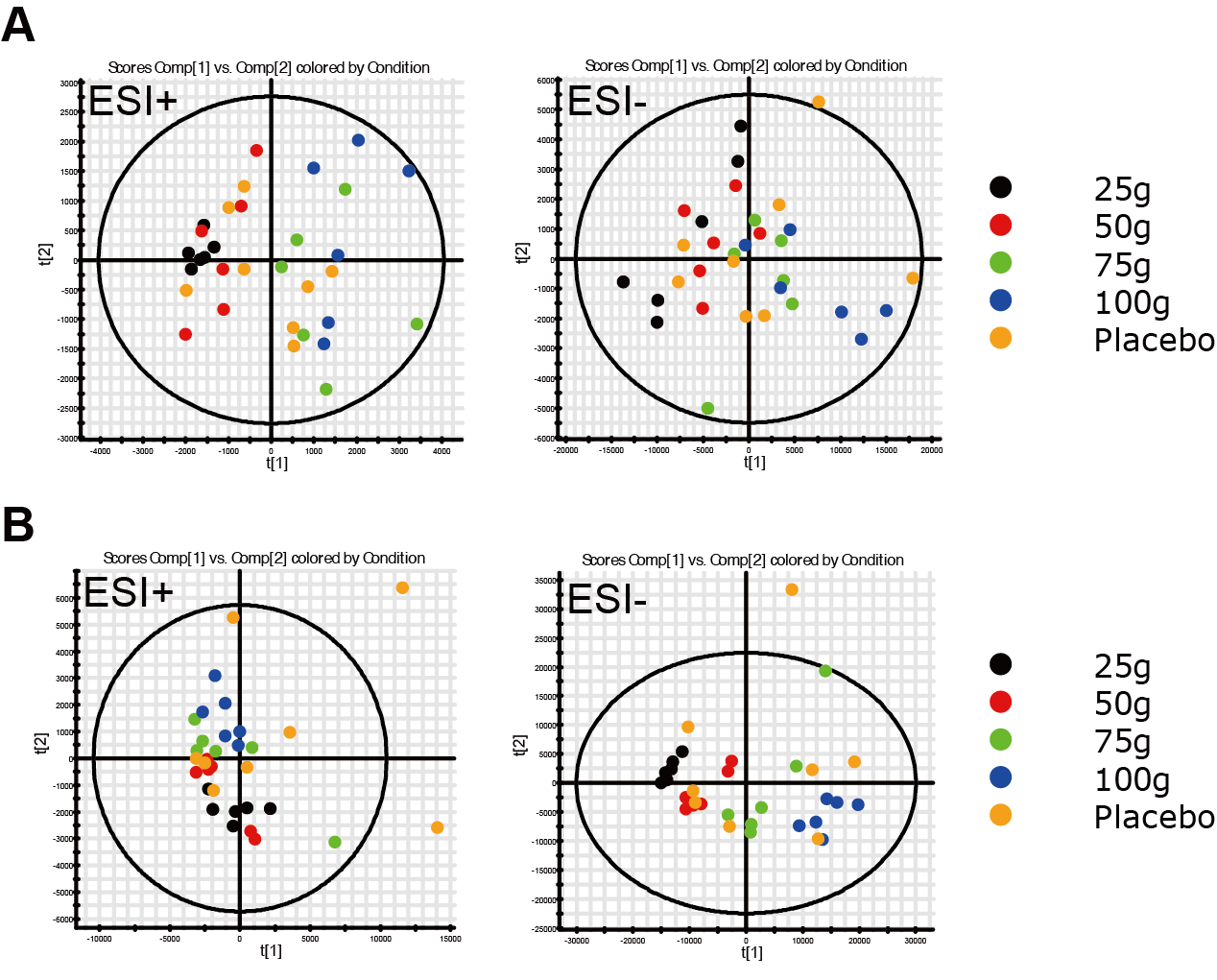


Fig. S5. Metabolomic characteristics induced by the different dosages of FQGBG. (A) PCA scoring plot of plasma collected 24h after treatment in both positive and negative ion modes. (ESI+ scores: R2X = 0.609, Q2 =0.144; ESI- scores: R2X = 0.891, Q2 =0.756). (B) PCA scoring plot of urine collected 48h after treatment in both positive and negative ion modes. (ESI+ scores: R2X = 0.767, Q2 =0.352; ESI- scores: R2X = 0.777, Q2 =0.523).


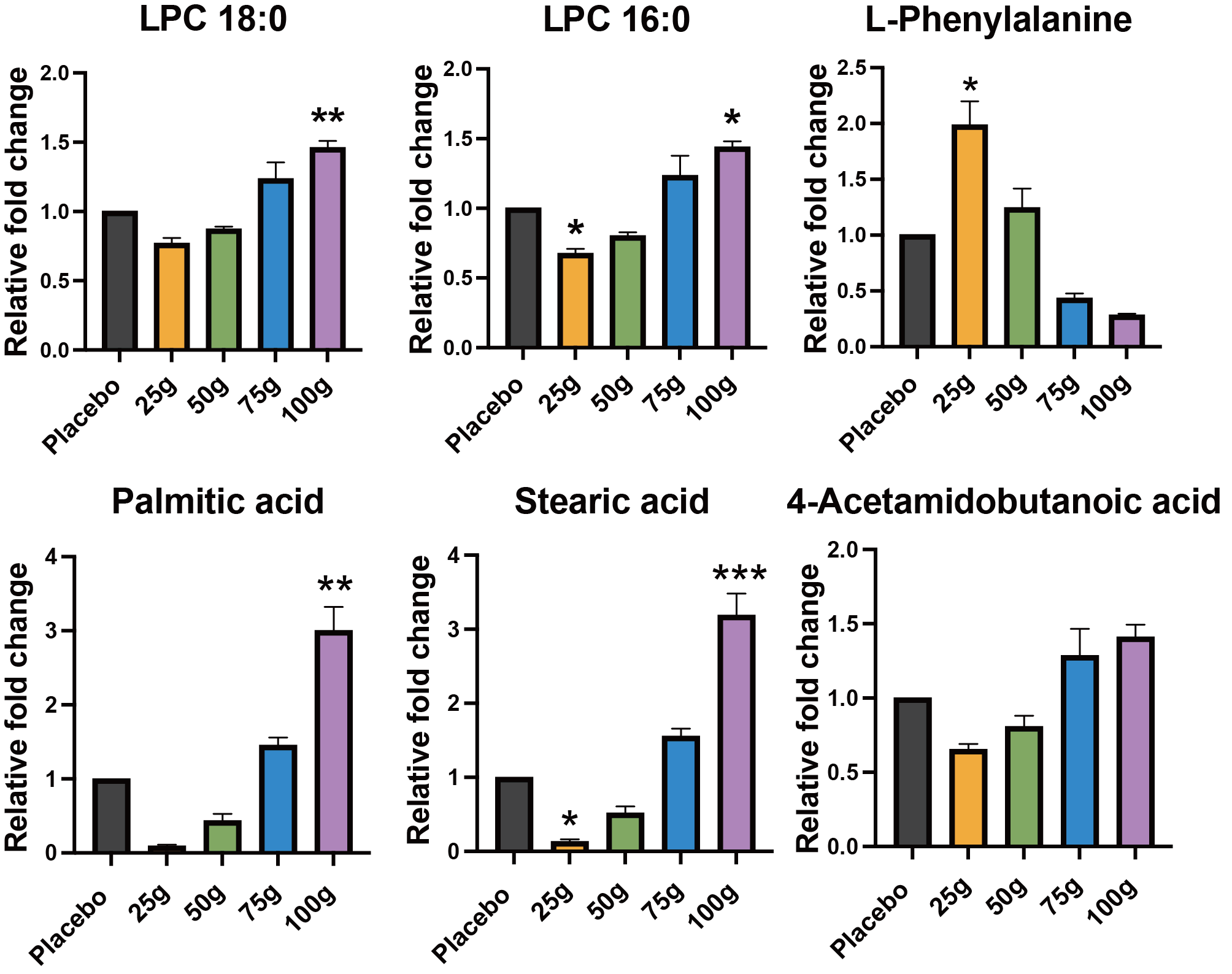


Fig. S6. The common changed metabolites induced by the different dosages of FQGBG. *p < 0.05, **p < 0.01, ***p < 0.001.

Fig. S7. MS/MS spectra of metabolites that displayed significant changes in response to the differential FQGBG treatments.

Stearoylcarnitine

| HMDB | 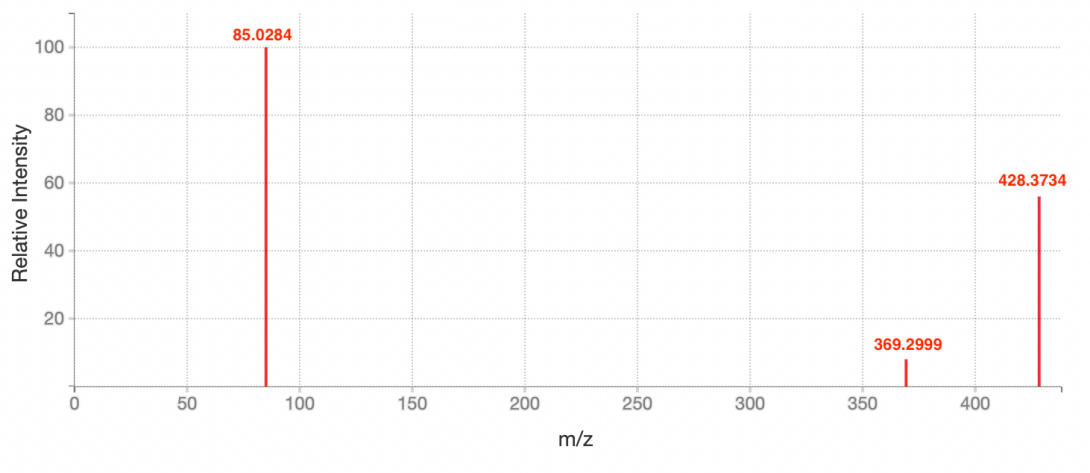 |
| --- | --- |
| Sample | 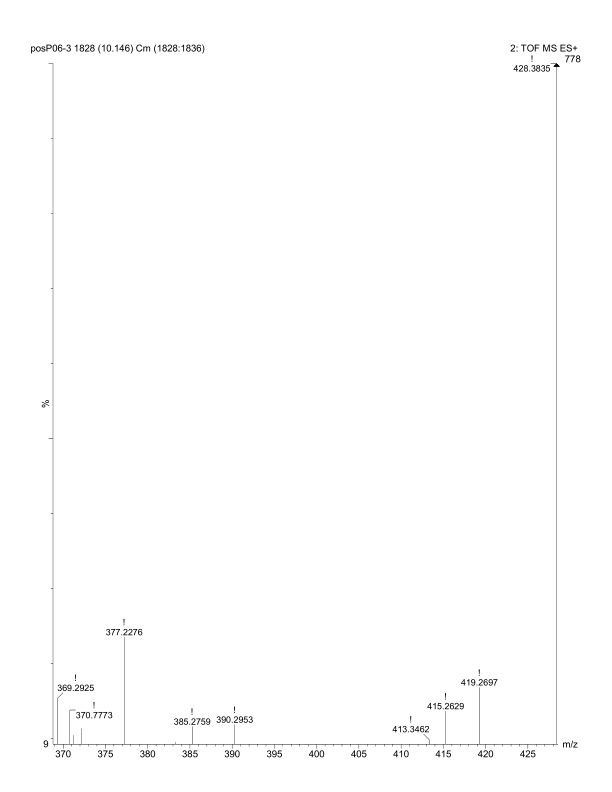 |

LPC18:0

| HMDB | 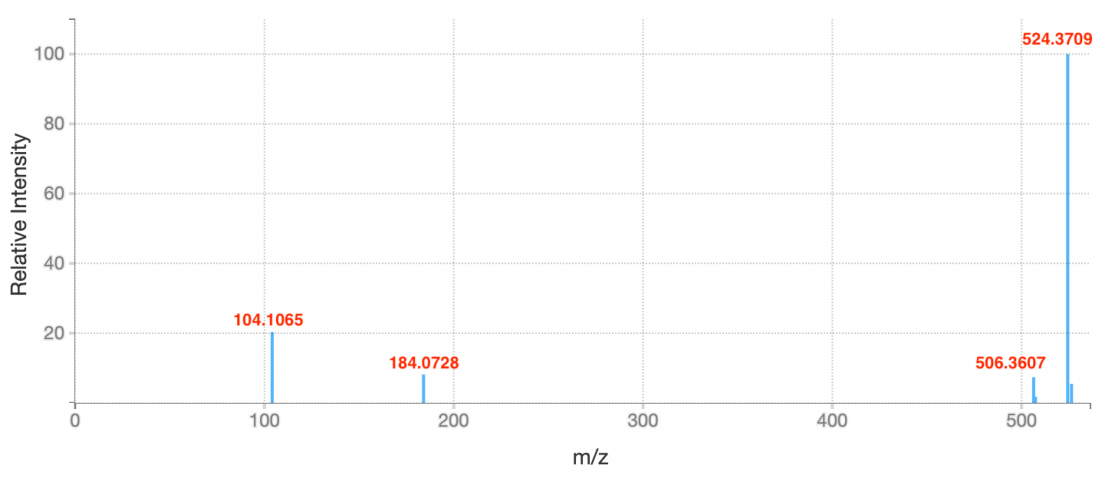 |
| --- | --- |
| Sample | 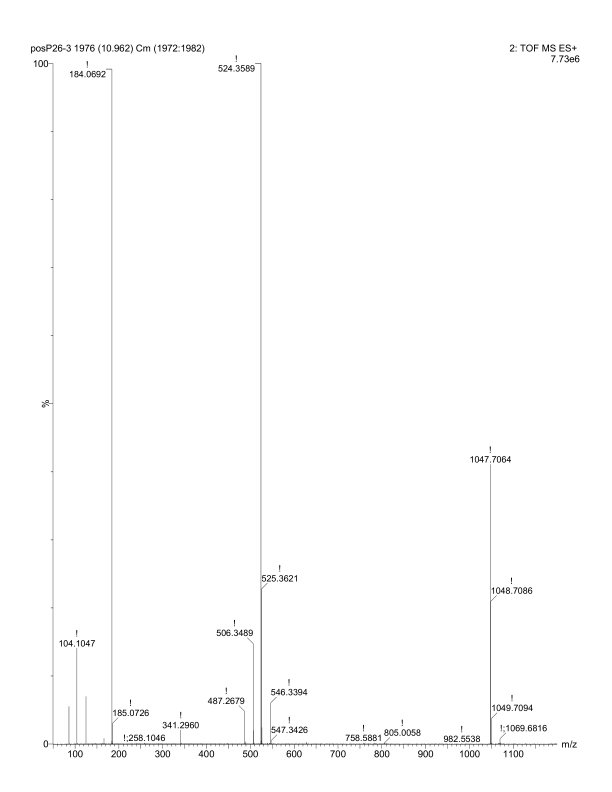 |

LPC(18:3(6Z,9Z,12Z))

| HMDB | 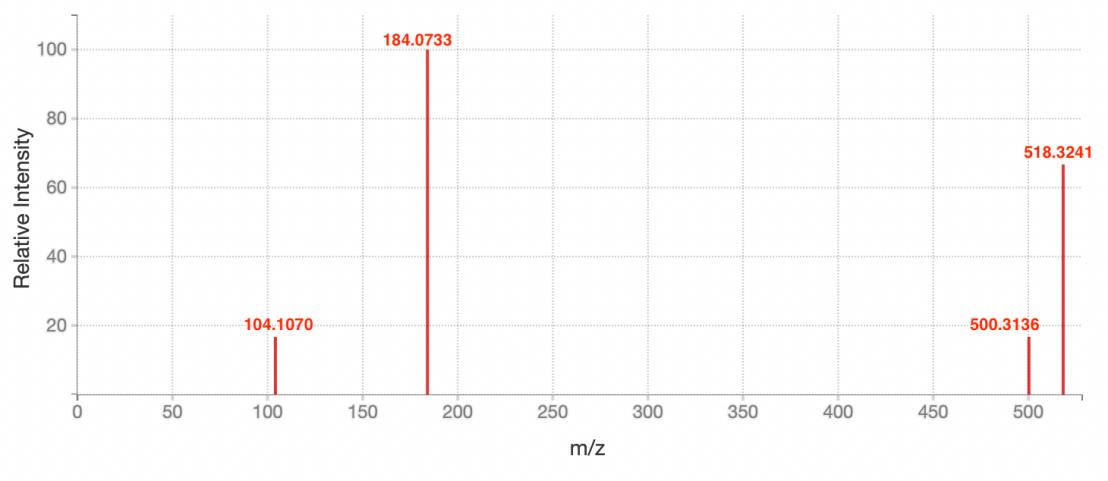 |
| --- | --- |
| Sample | 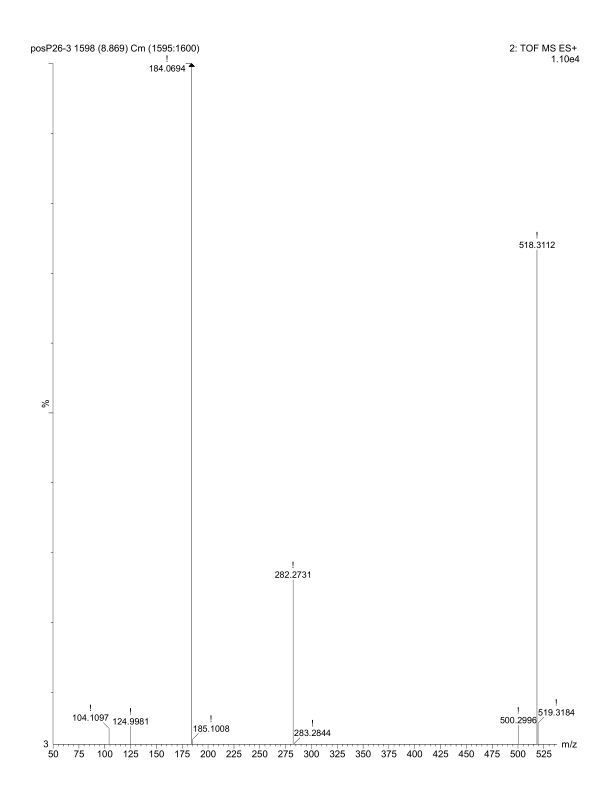 |

LPC(20:3(5Z,8Z,11Z))

| HMDB | 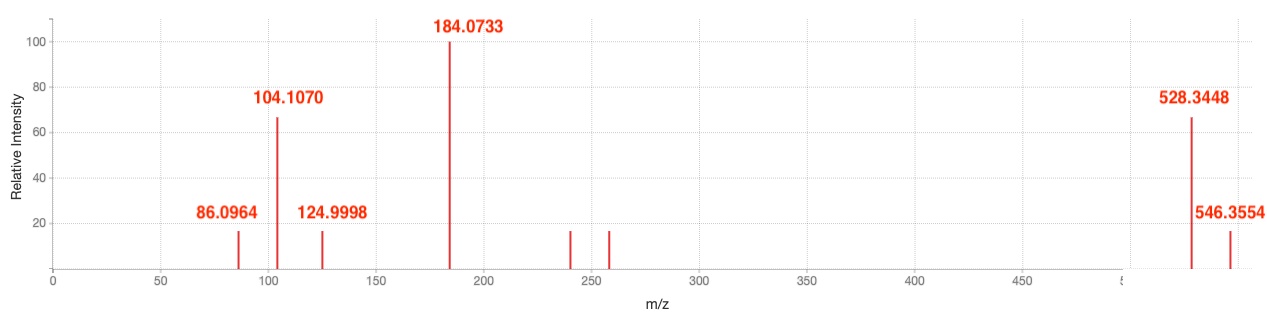 |
| --- | --- |
| Sample | 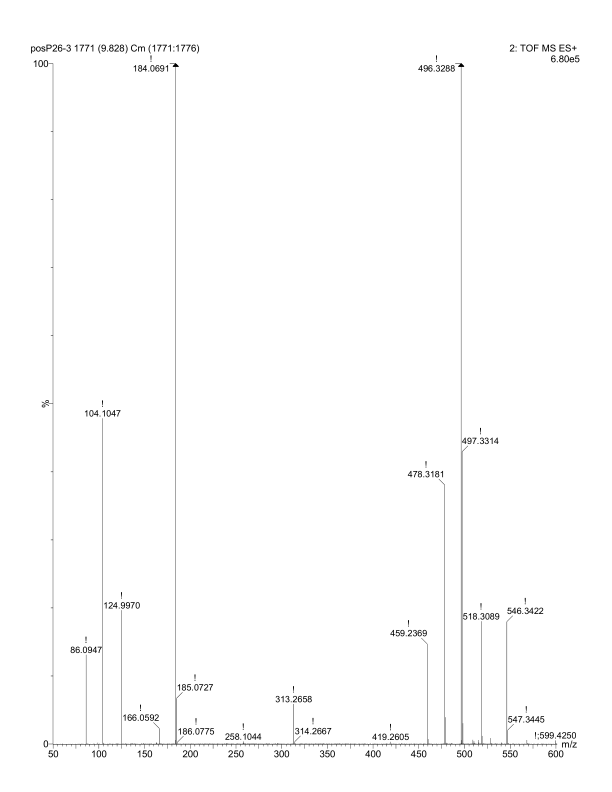 |

Oleic acid

| HMDB | 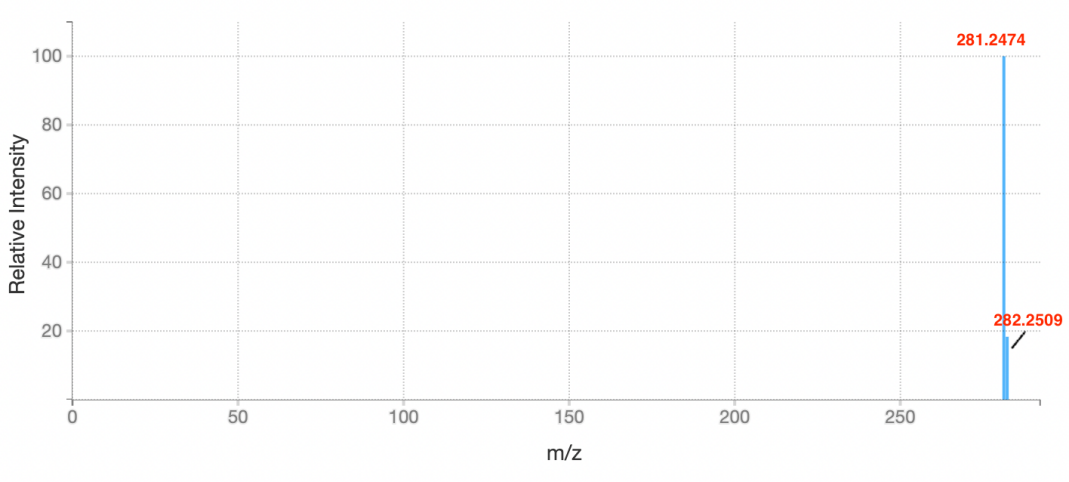 |
| --- | --- |
| Sample | 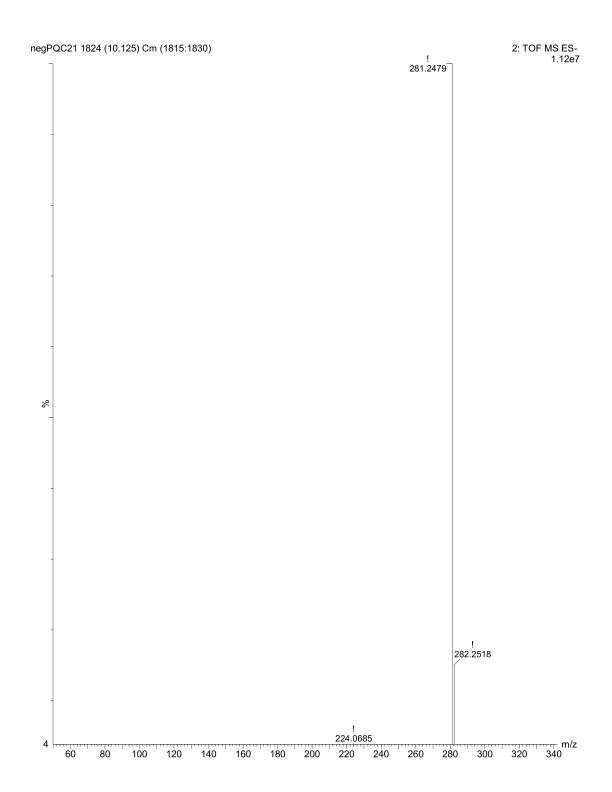 |

Stearic acid

| HMDB | 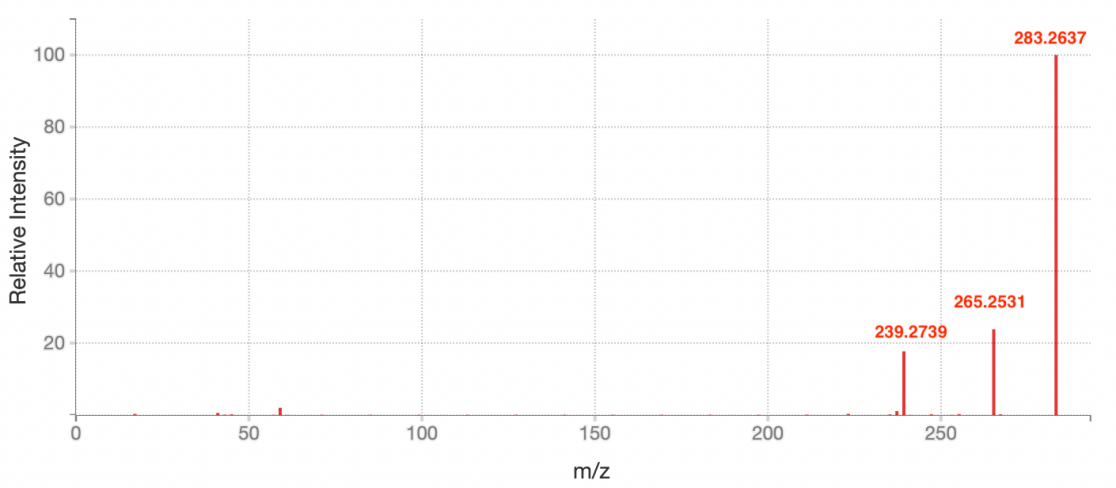 |
| --- | --- |
| Sample | 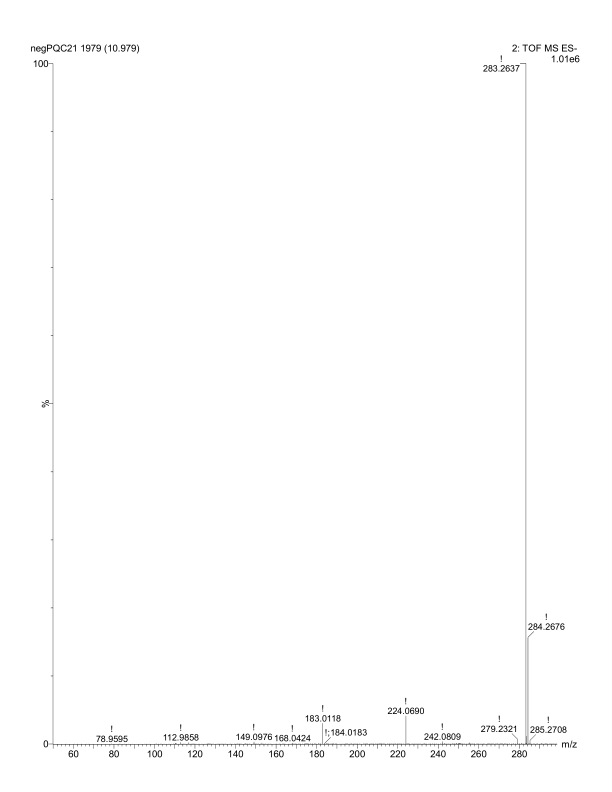 |

Palmitic acid

| HMDB | 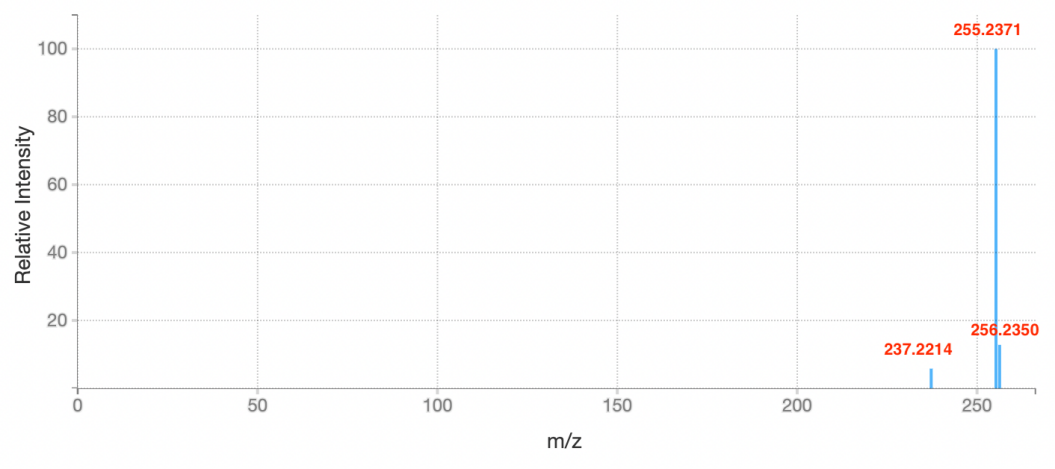 |
| --- | --- |
| Sample | 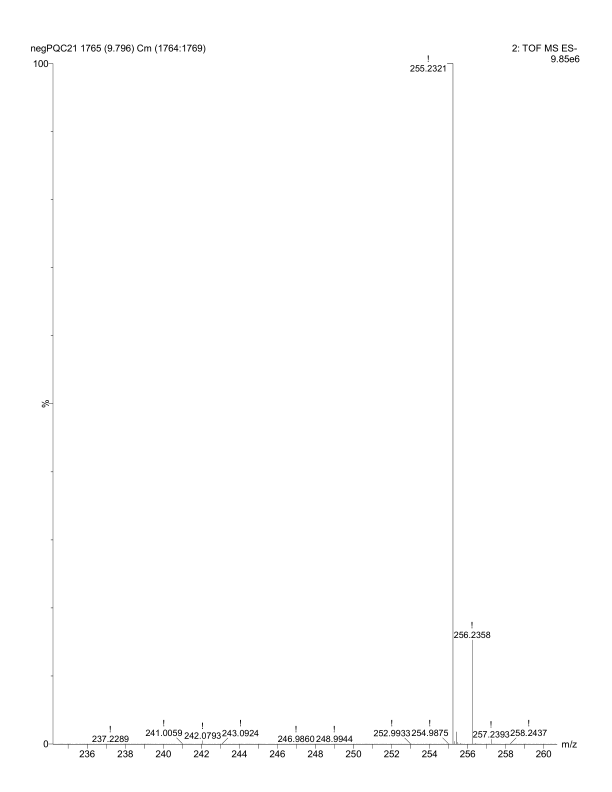 |

4-acetamidobutanoic acid

| HMDB | 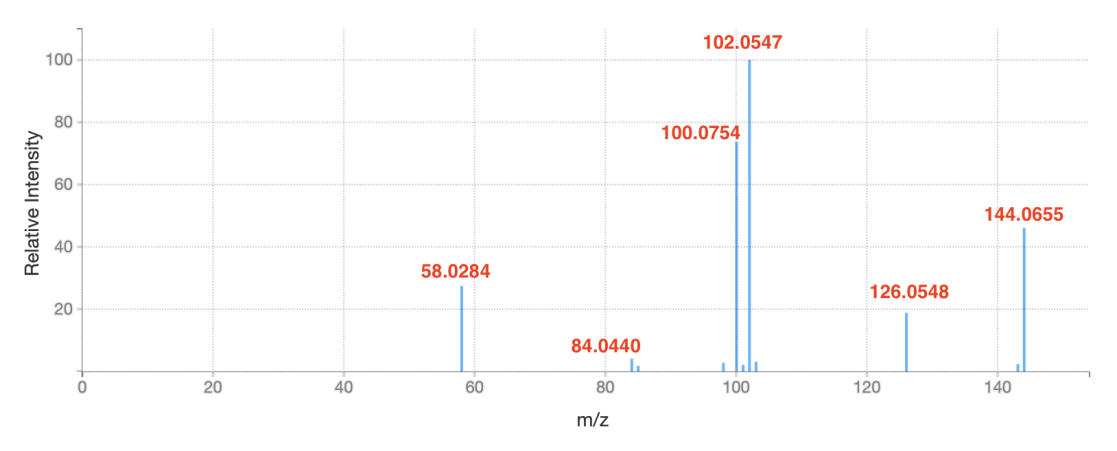 |
| --- | --- |
| Sample | 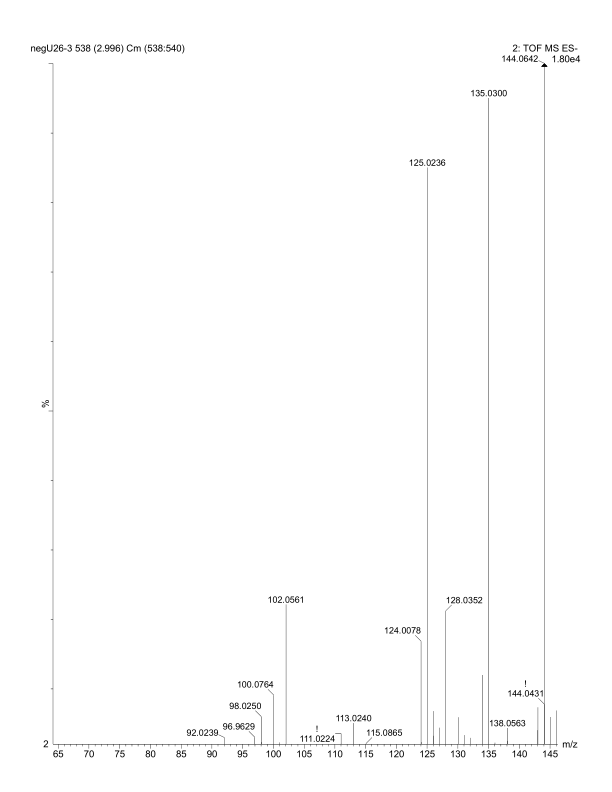 |

Ornithine

| HMDB | 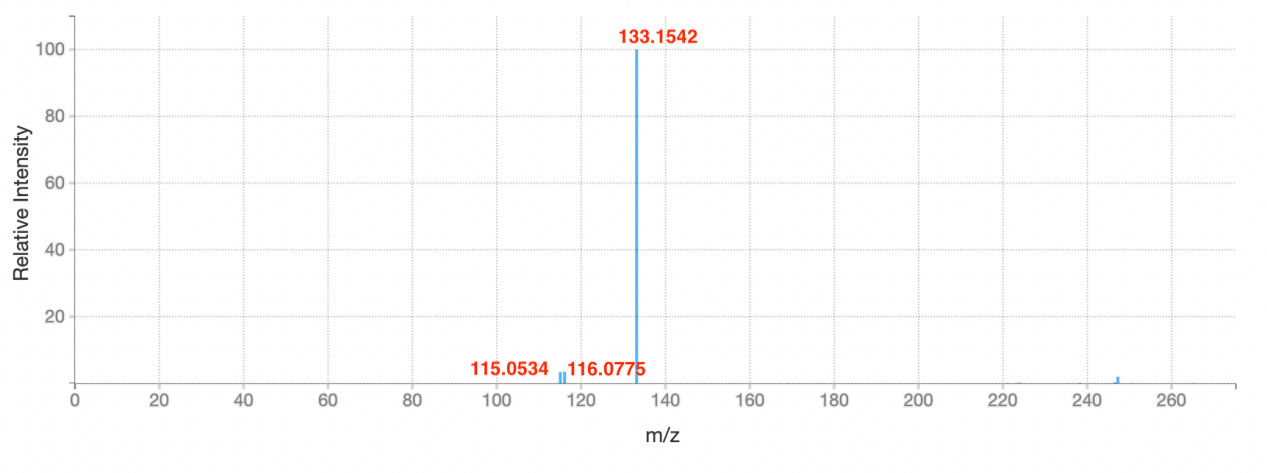 |
| --- | --- |
| Sample | 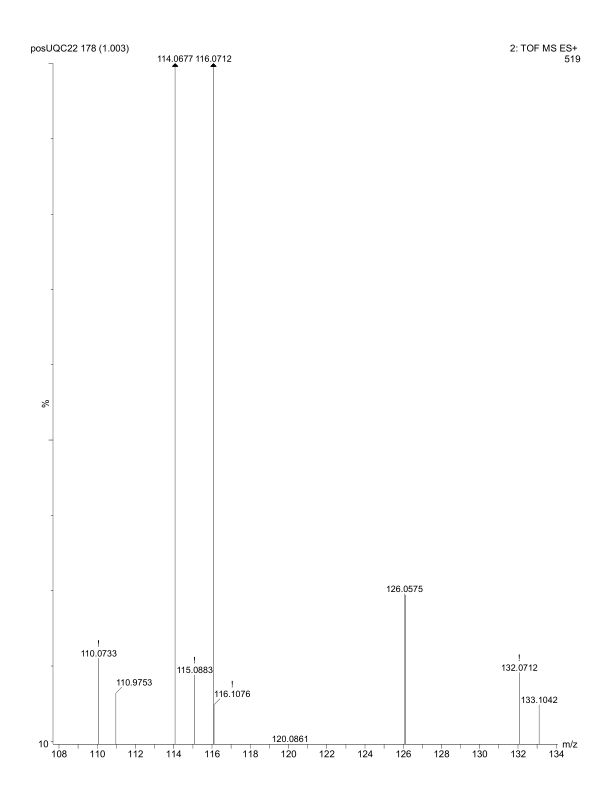 |

L-Proline

| HMDB | 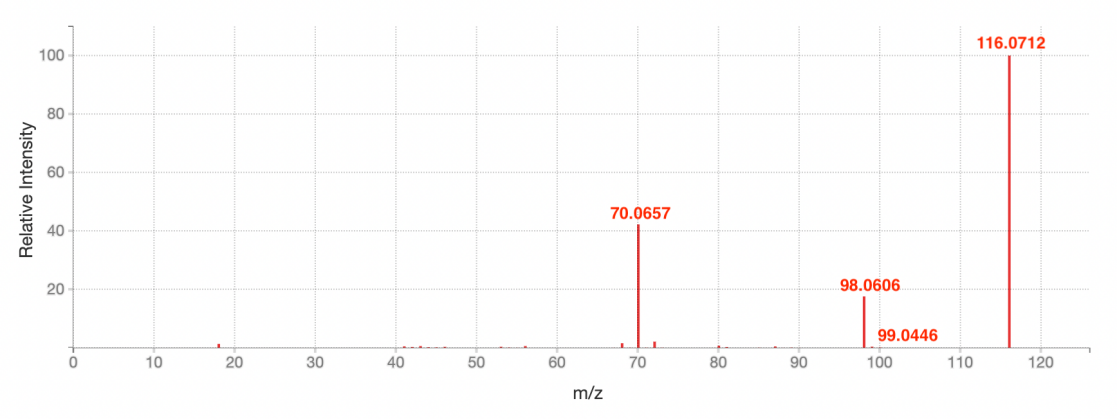 |
| --- | --- |
| Sample | 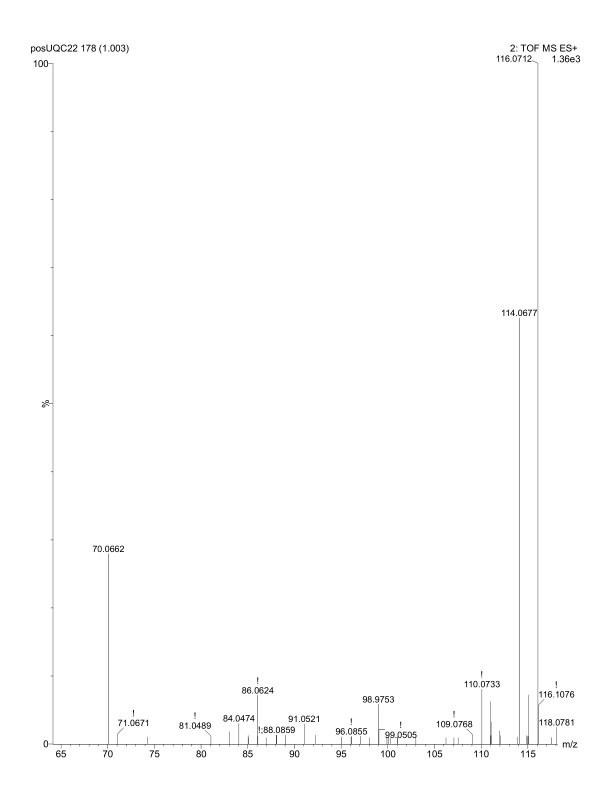 |

Tryptophan

| Mass  Bank | 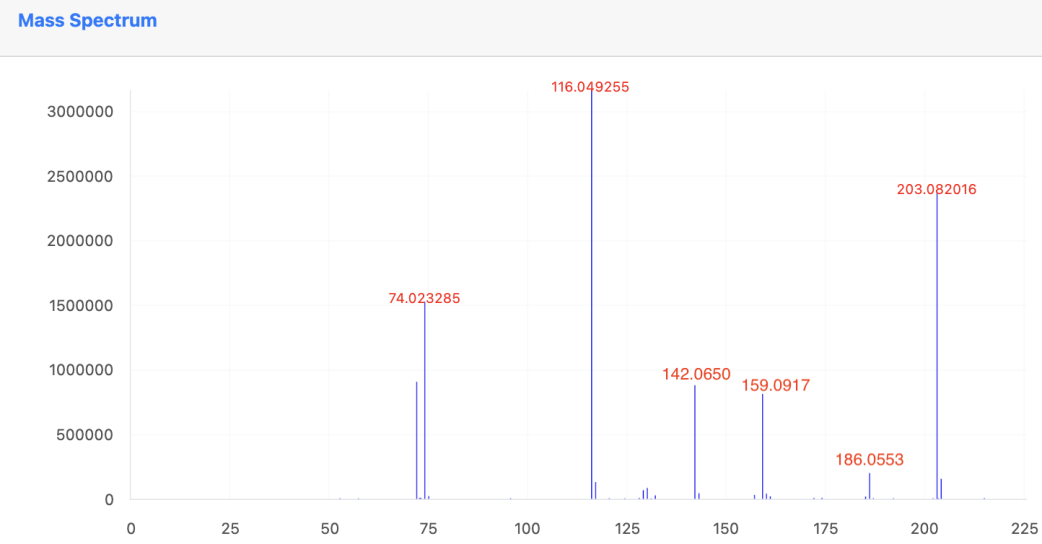 |
| --- | --- |
| Sample | 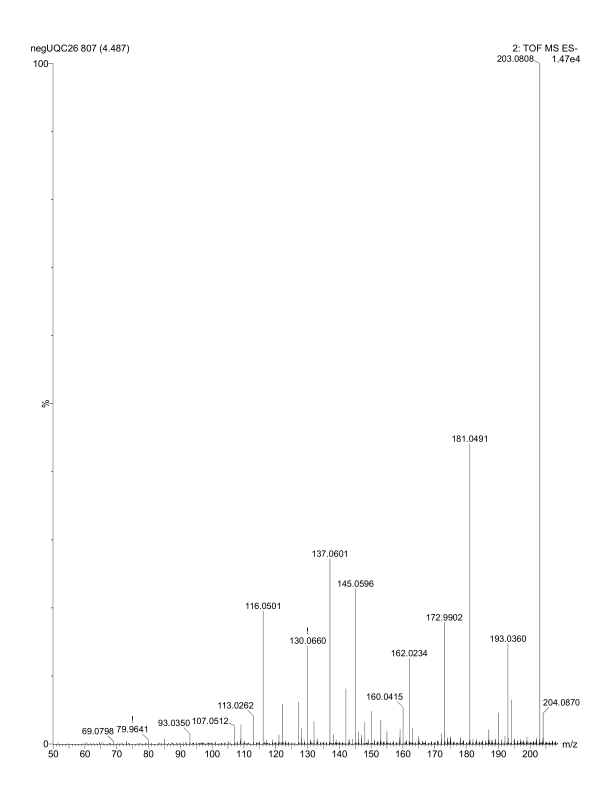 |

Uric acid

| Mass  Bank | 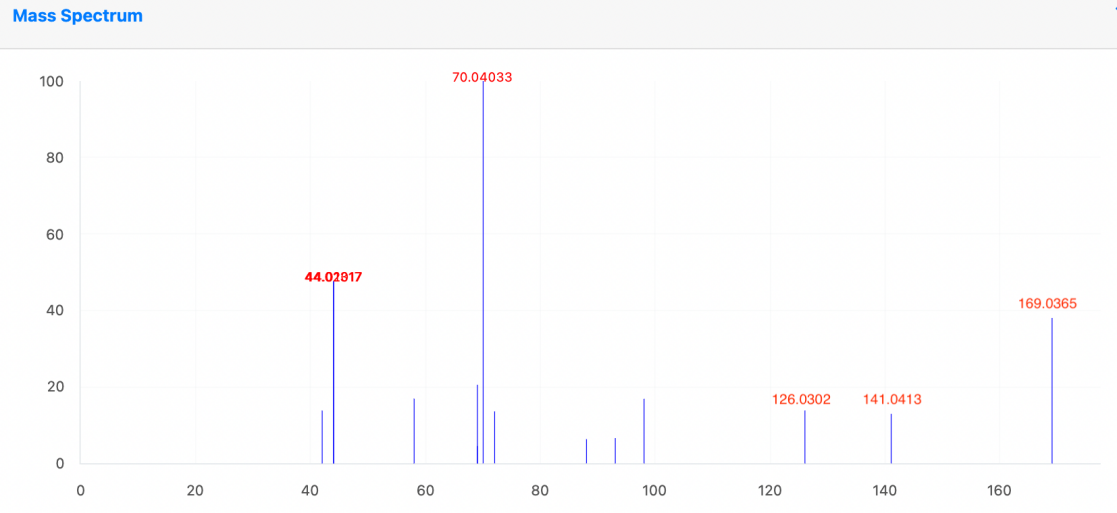 |
| --- | --- |
| Sample | 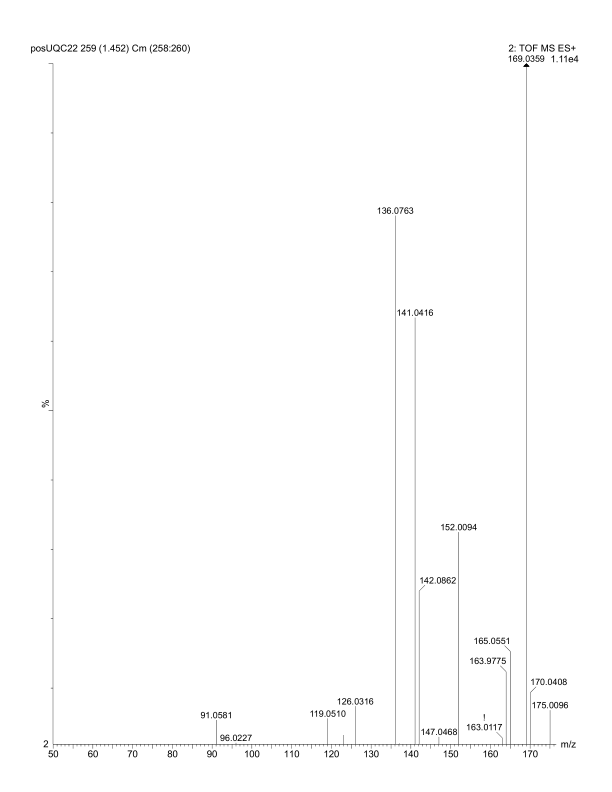 |

1-Methylguanine

| HMDB | 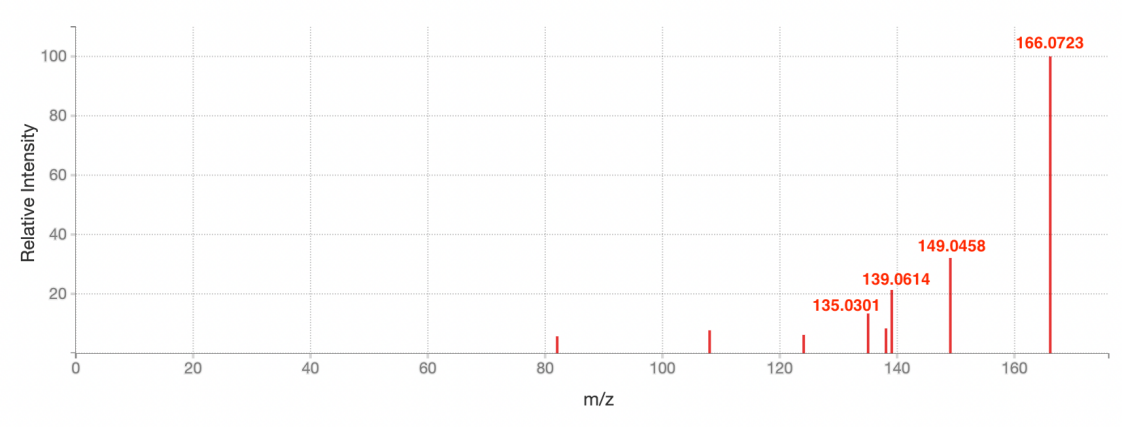 |
| --- | --- |
| Sample | 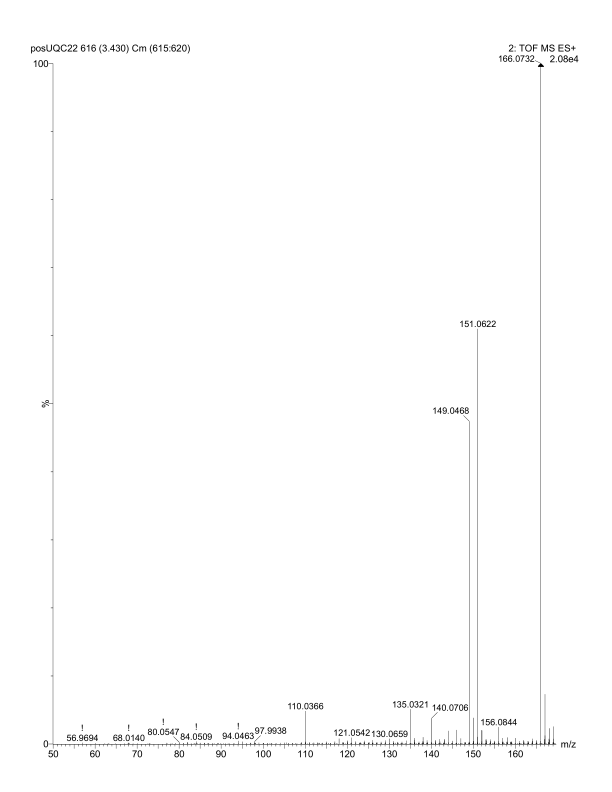 |

Succinyladenosine

| HMDB | 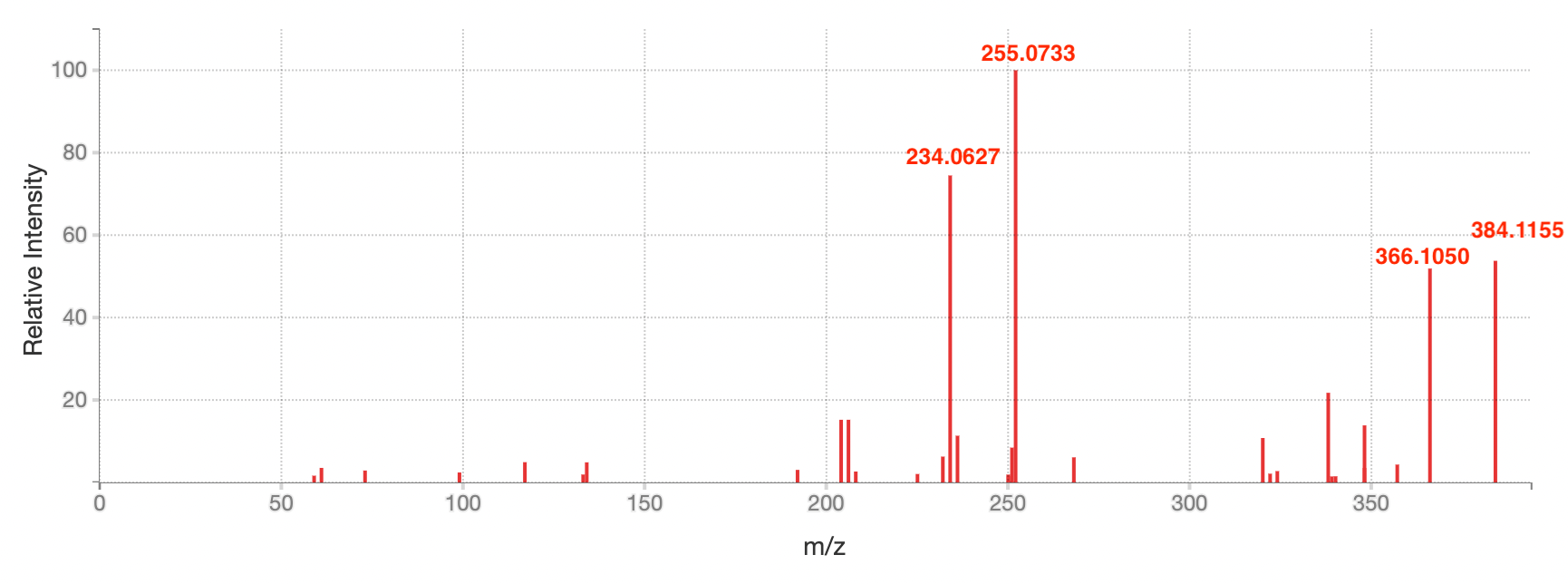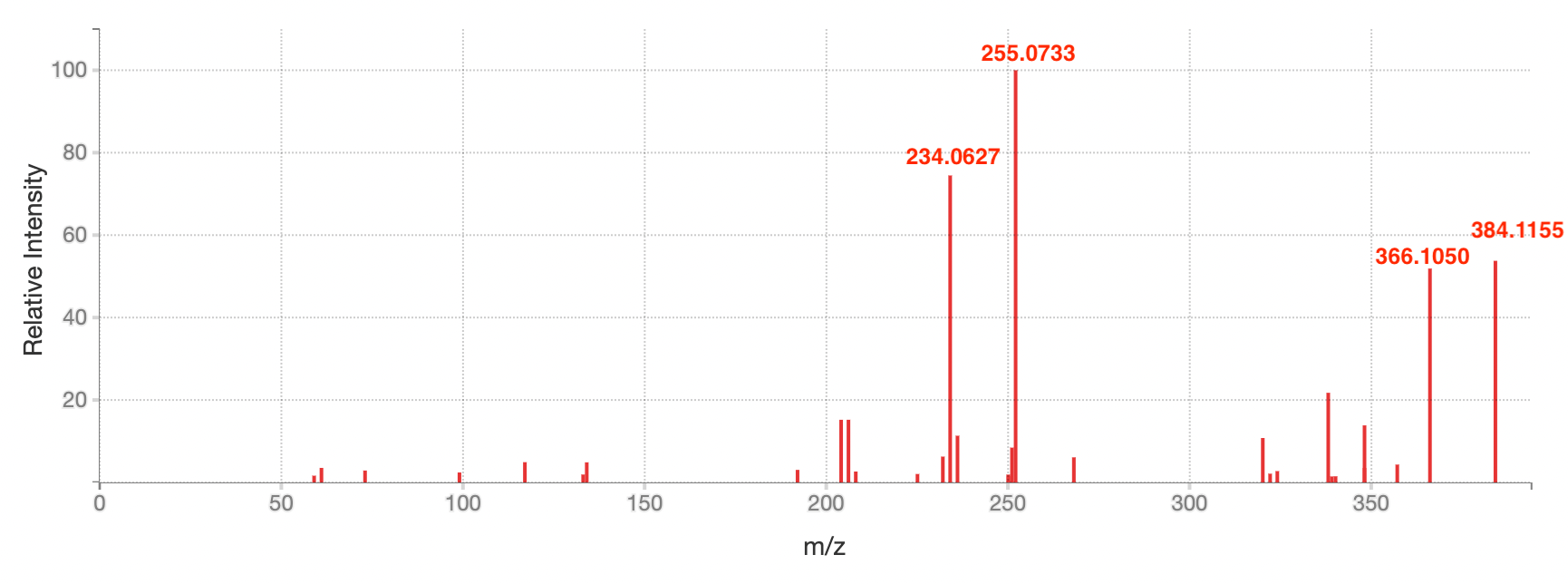 |
| --- | --- |
| Sample | 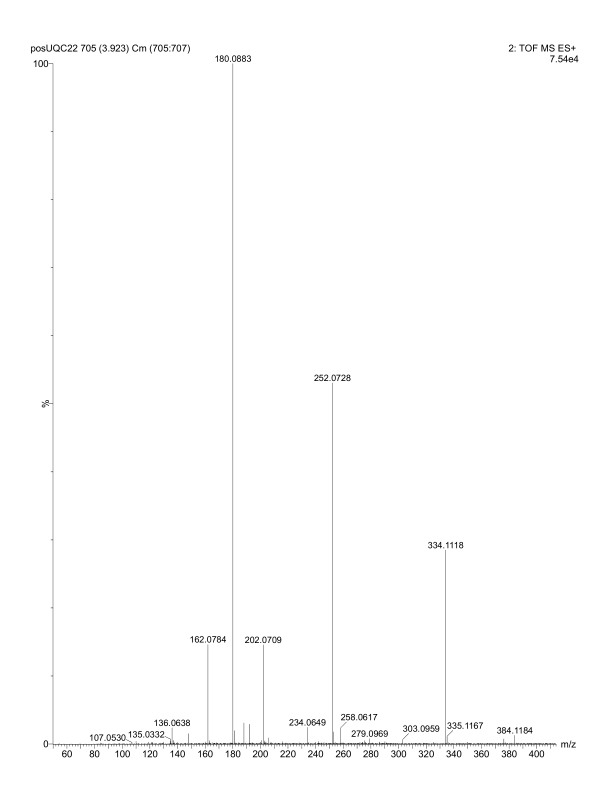 |

5-Hydroxyindoleacetaldehyde

| HMDB | 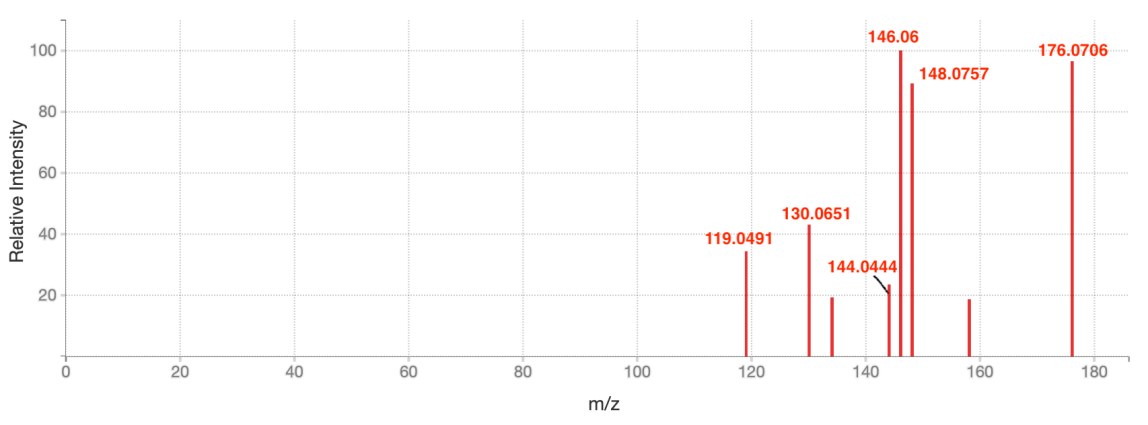 |
| --- | --- |
| Sample | 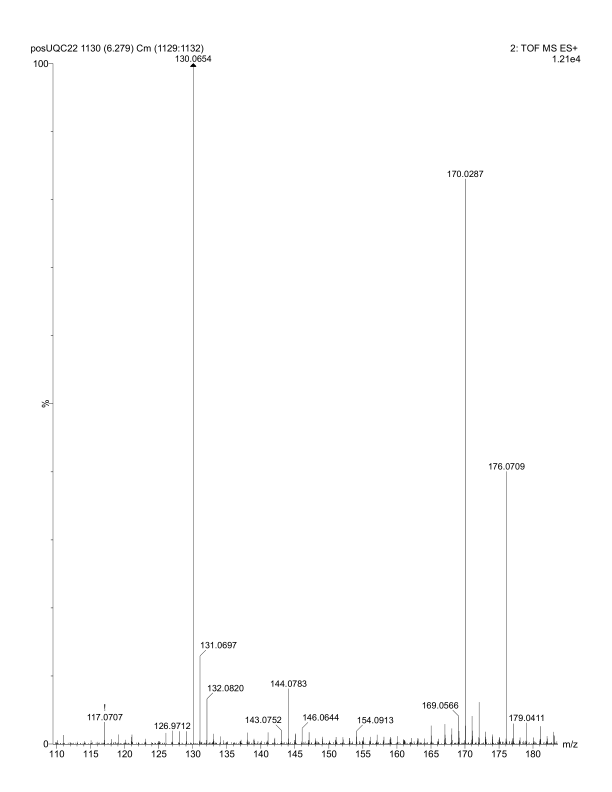 |

2-Indolecarboxylic acid

| HMDB | 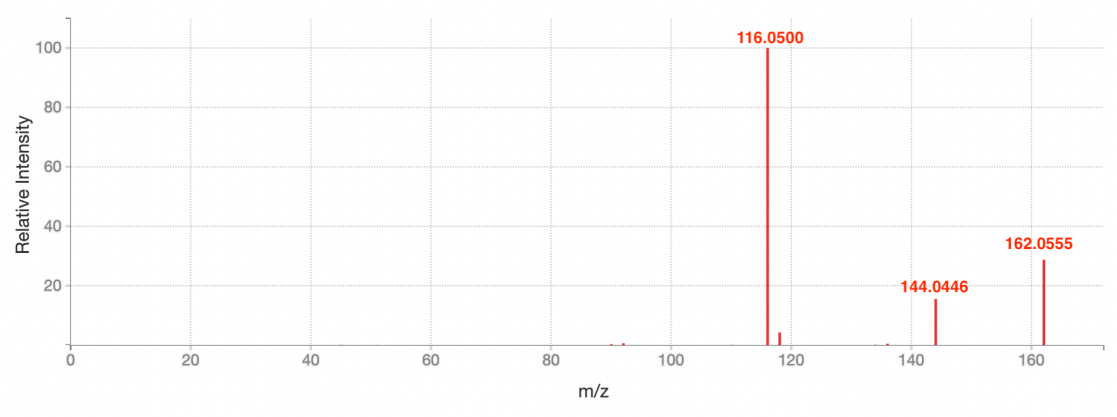 |
| --- | --- |
| Sample | 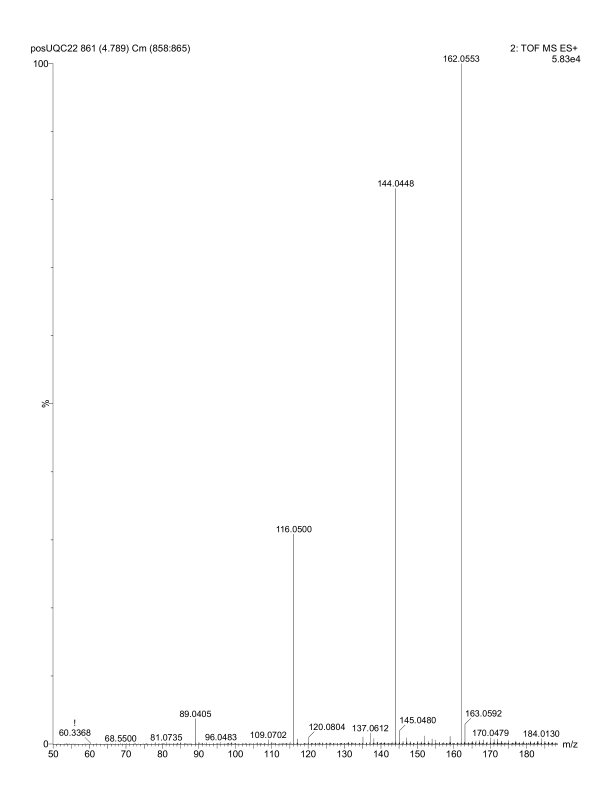 |

Kynurenic acid

| HMDB | 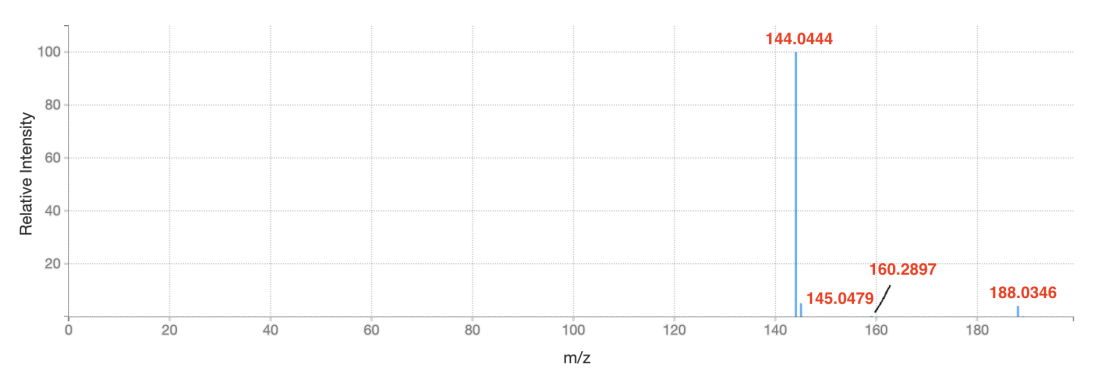 |
| --- | --- |
| Sample | 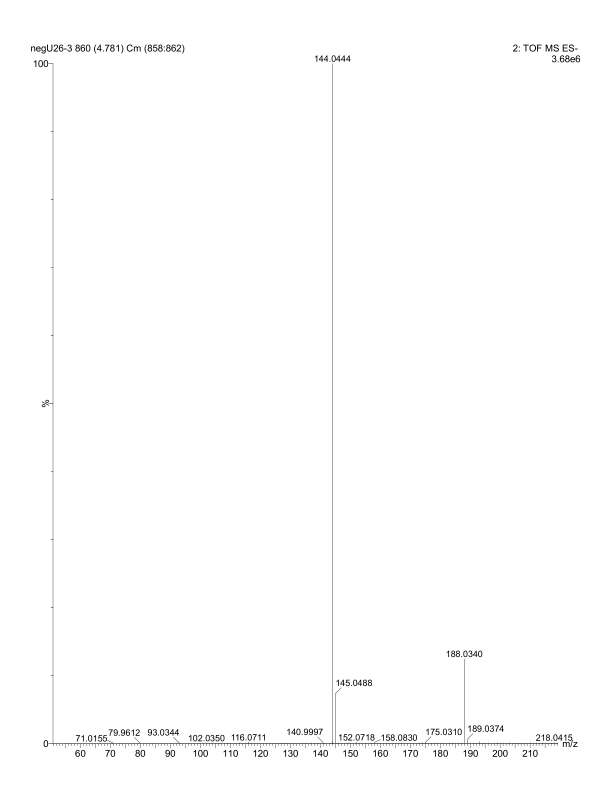 |

Glutaric acid

| HMDB | 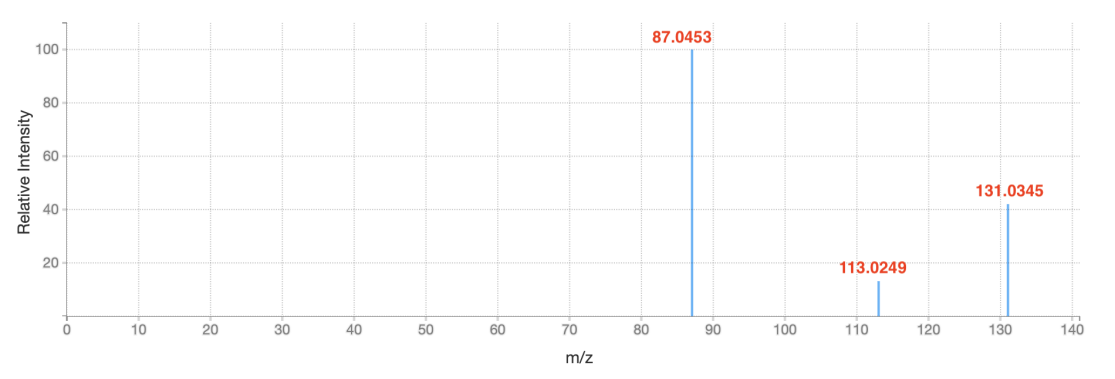 |
| --- | --- |
| Sample | 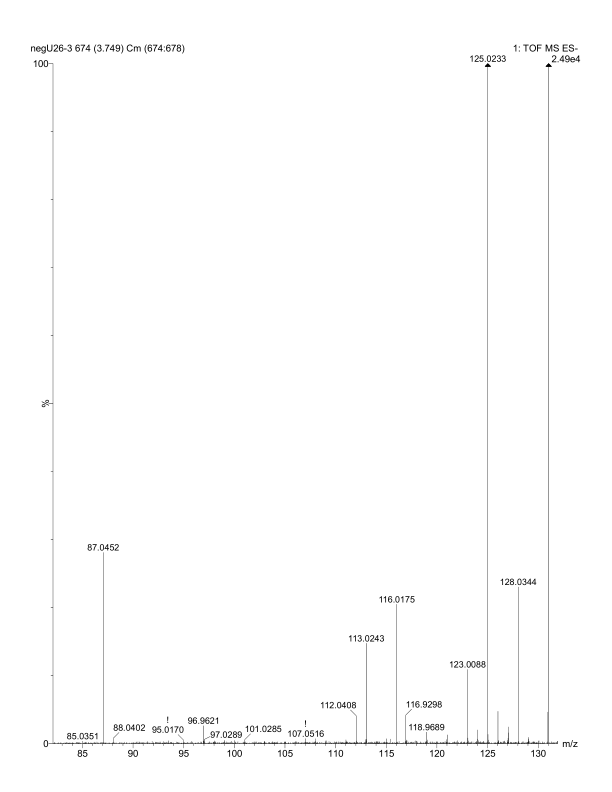 |

4-Hydroxyproline

| HMDB | 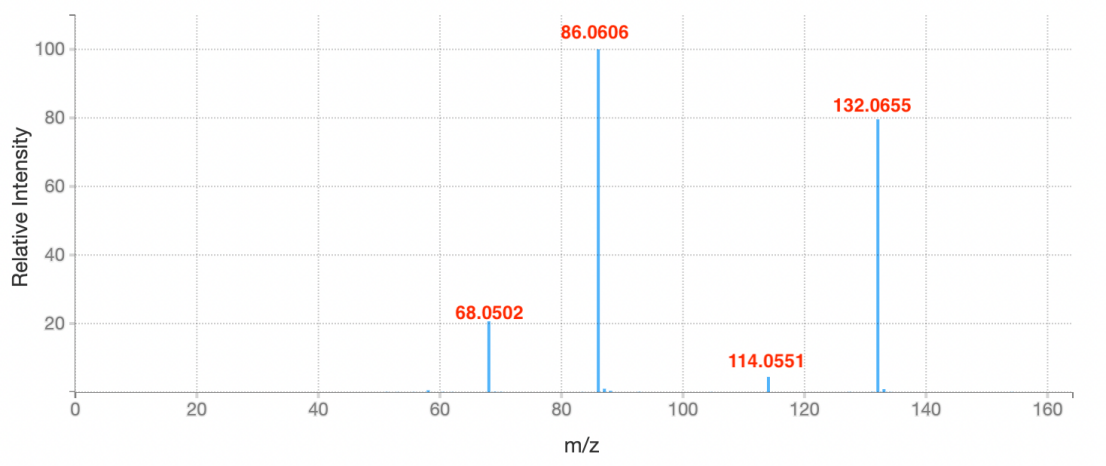 |
| --- | --- |
| Sample | 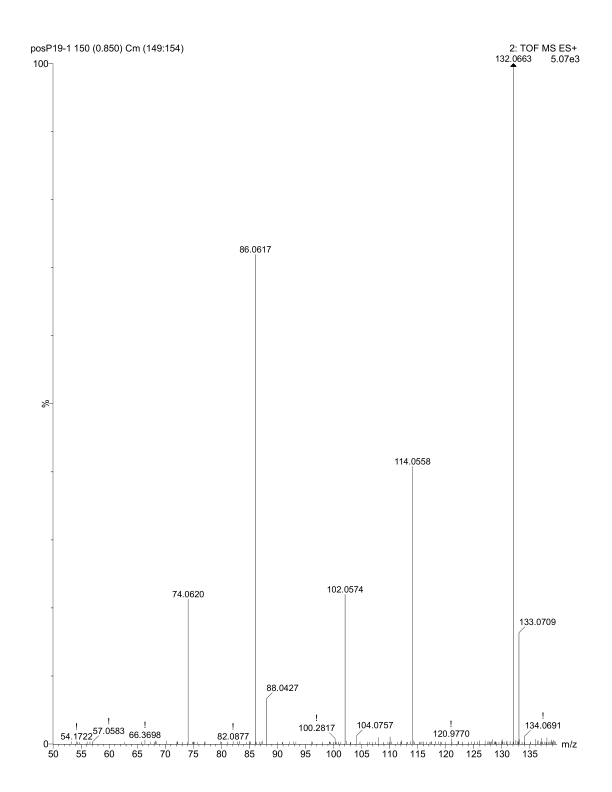 |

L-Phenylalanine

| Mass  Bank | 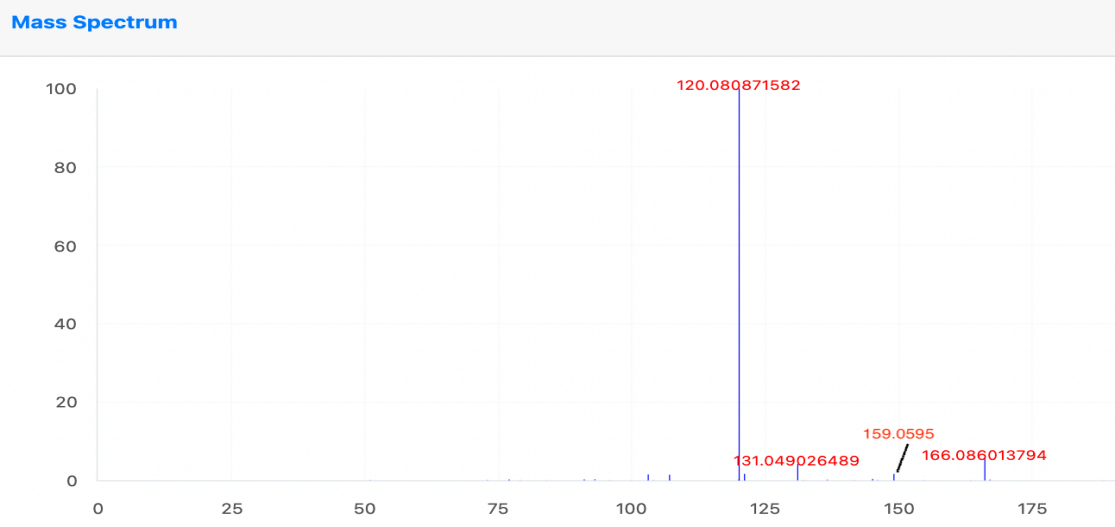 |
| --- | --- |
| Sample | 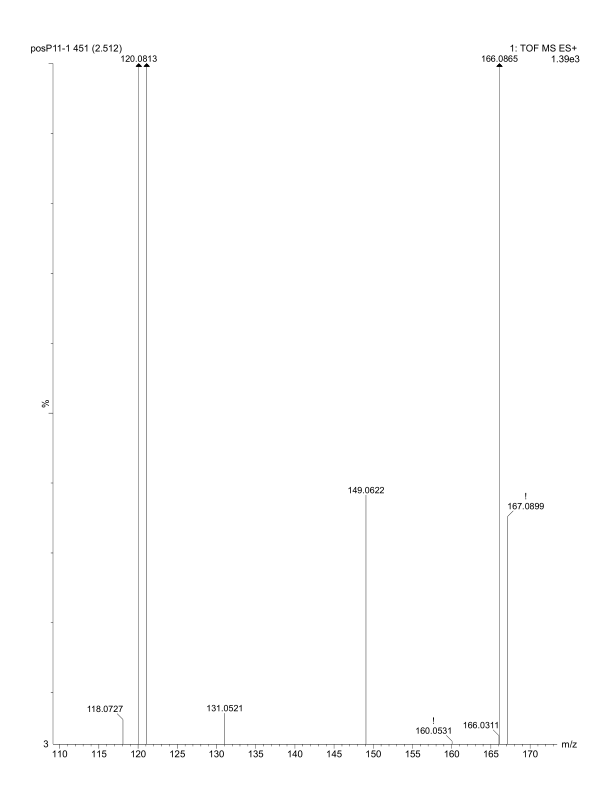 |

LPC16:0

| HMDB | 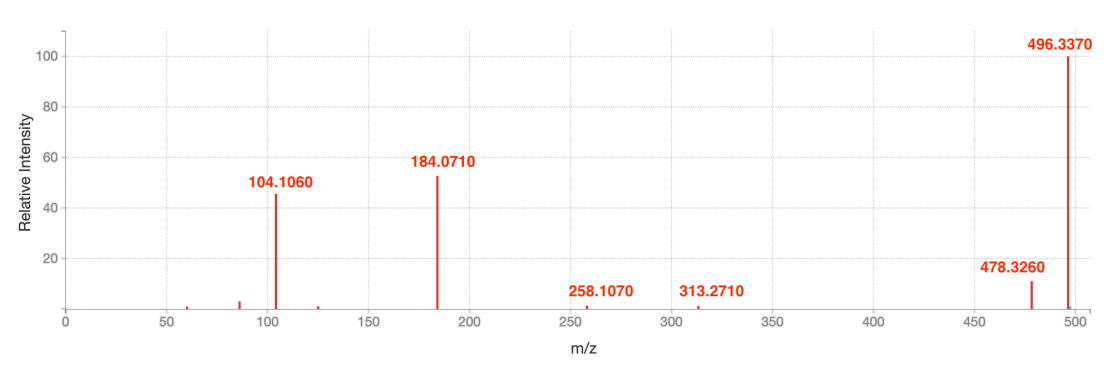 |
| --- | --- |
| Sample | 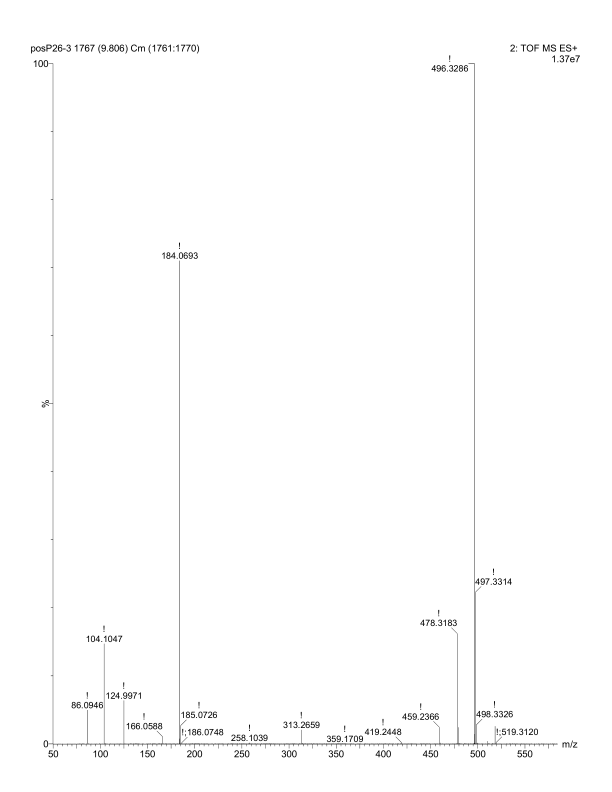 |

Heptadecanoic acid

| Mass  Bank | 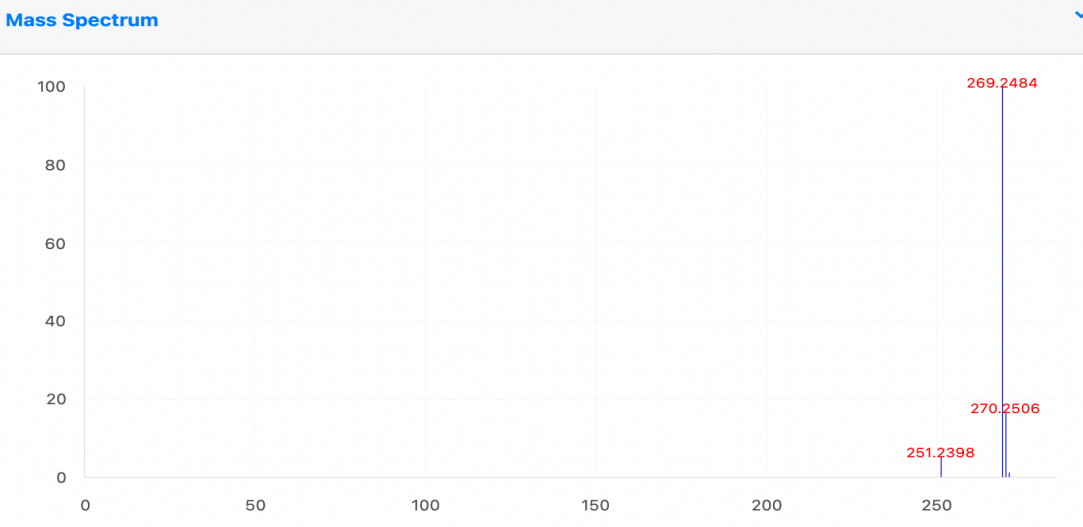 |
| --- | --- |
| Sample | 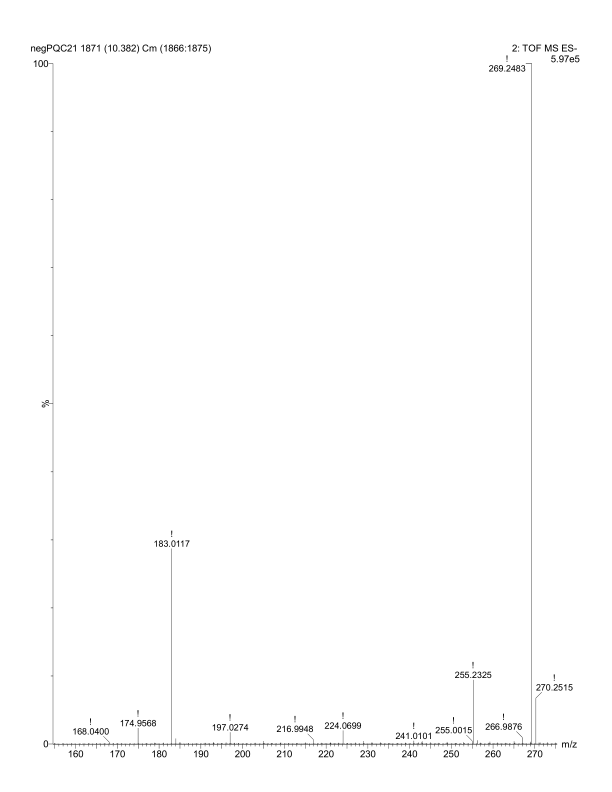 |

Myristic acid

| Mass  Bank | 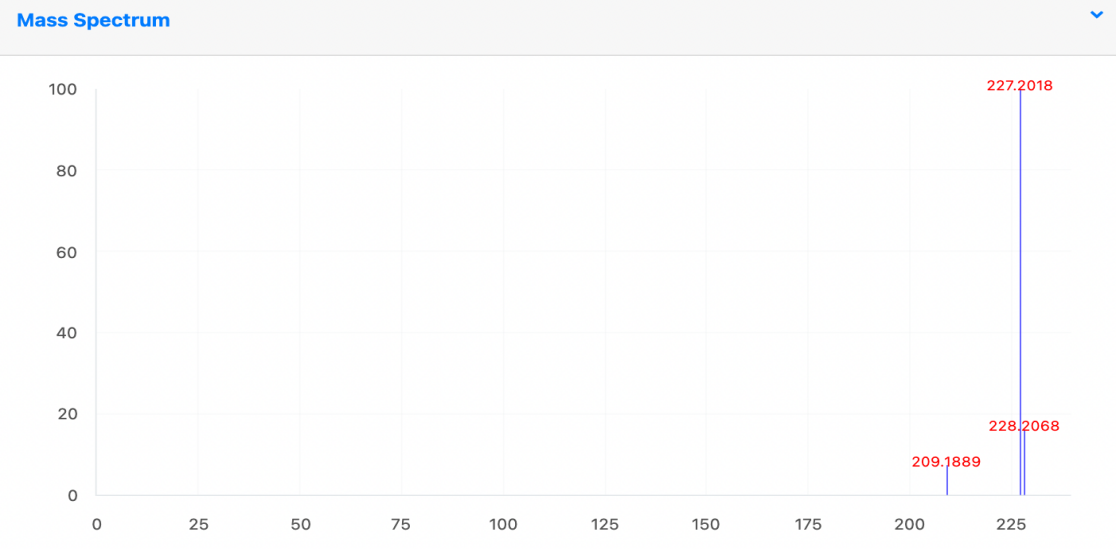 |
| --- | --- |
| Sample | 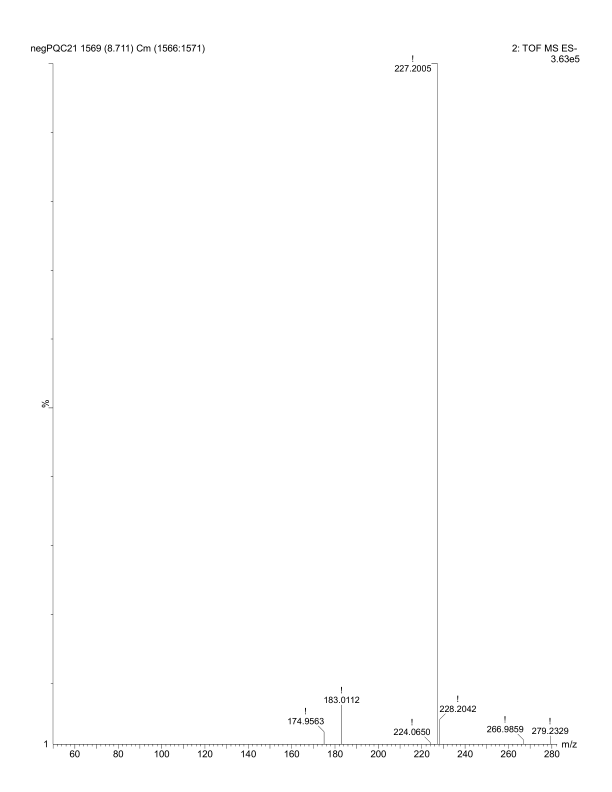 |

Linoelaidic acid

| Mass  Bank | 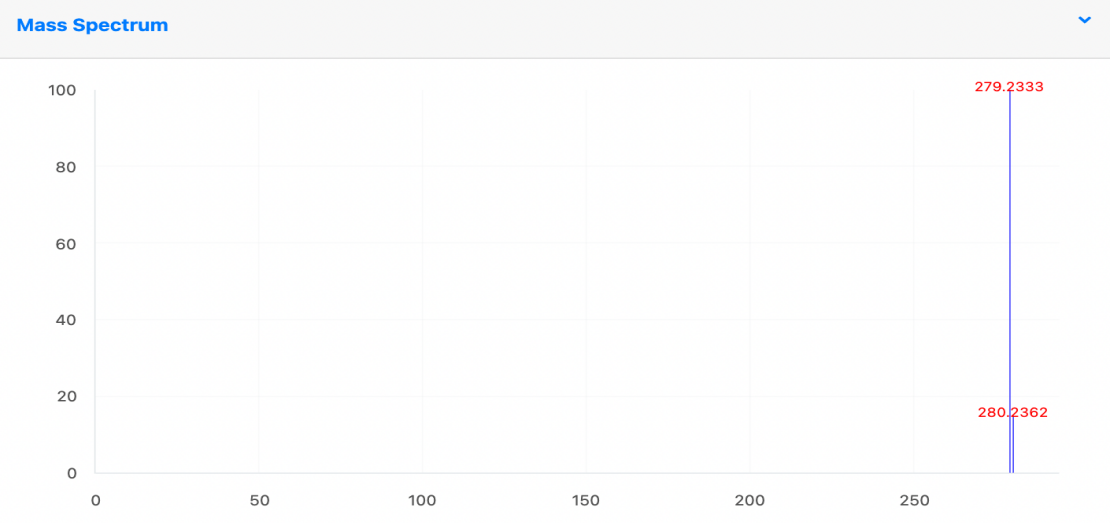 |
| --- | --- |
| Sample | 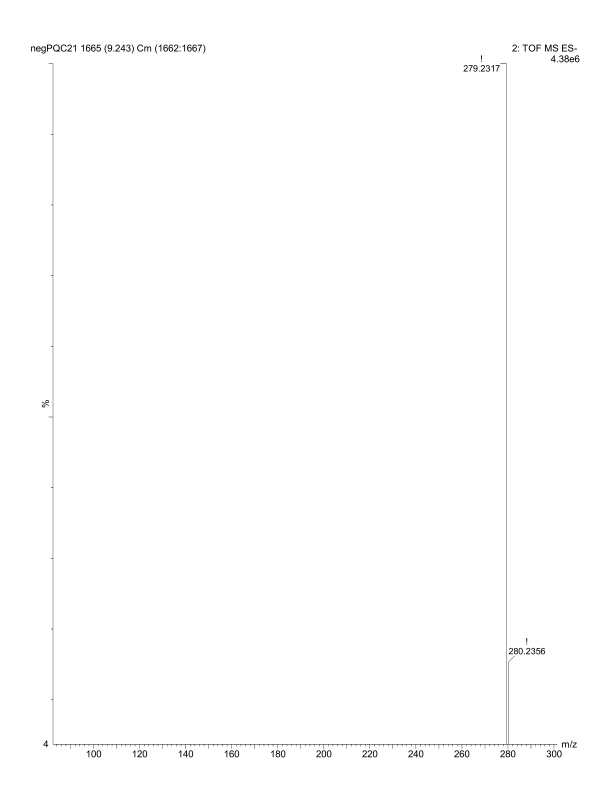 |

8-Hydroxy-7-methylguanine

| HMDB | 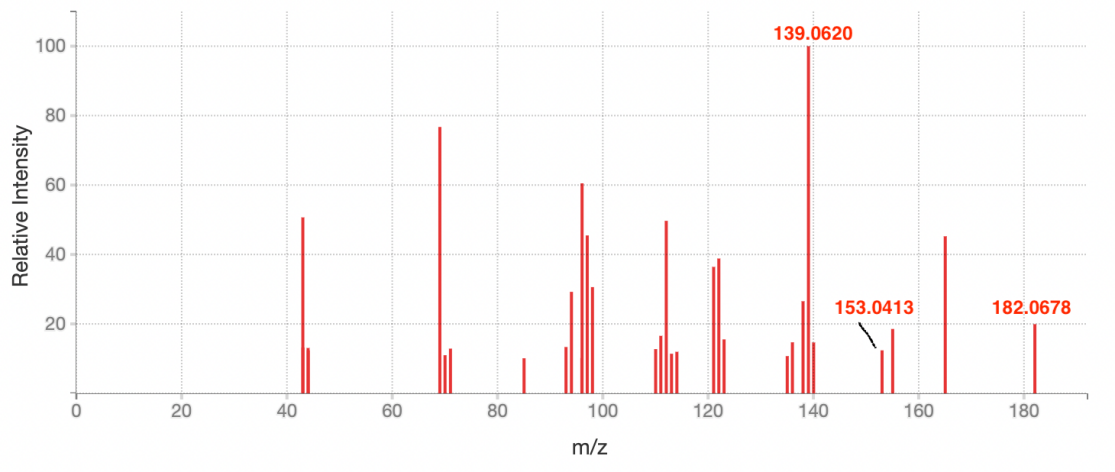 |
| --- | --- |
| Sample | 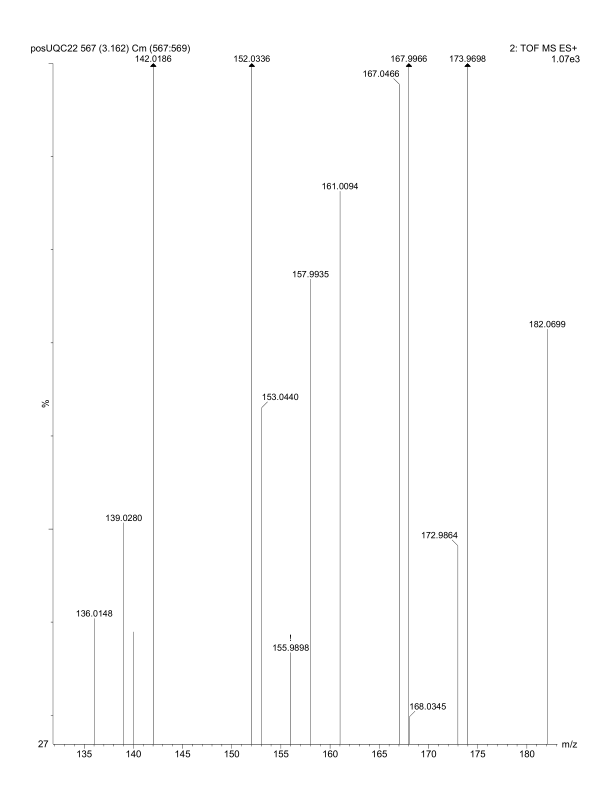 |

3-Hydroxyoctanoyl carnitine

| HMDB | 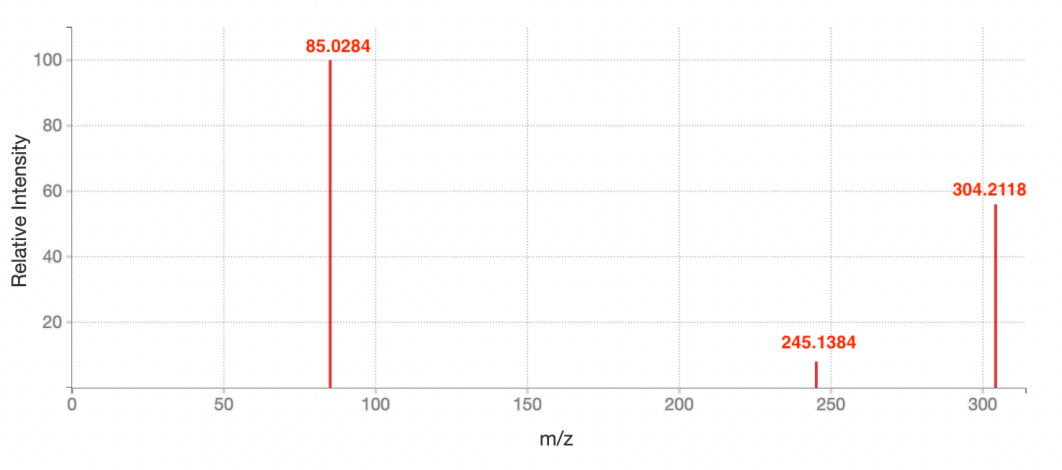 |
| --- | --- |
| Sample | 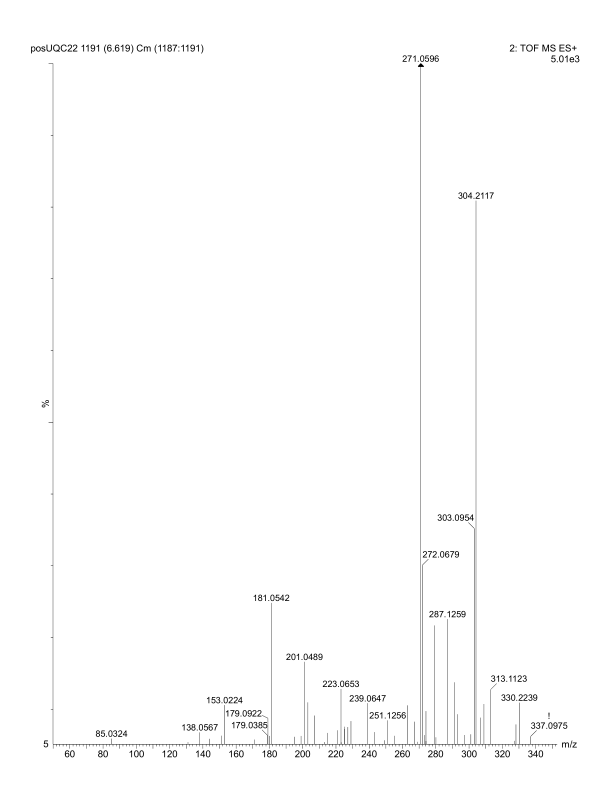 |

PC(18:3(6Z,9Z,12Z)/16:0)

| HMDB | 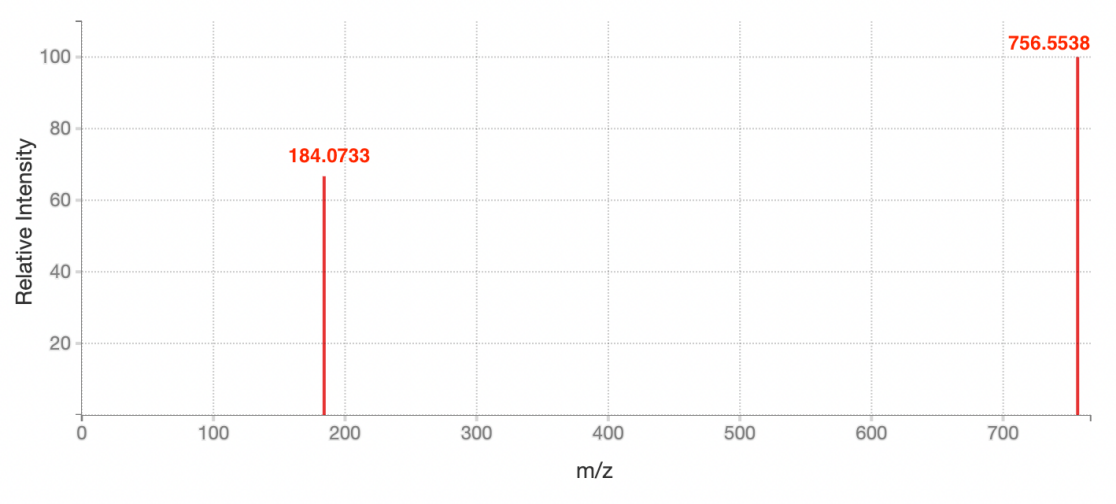 |
| --- | --- |
| Sample | 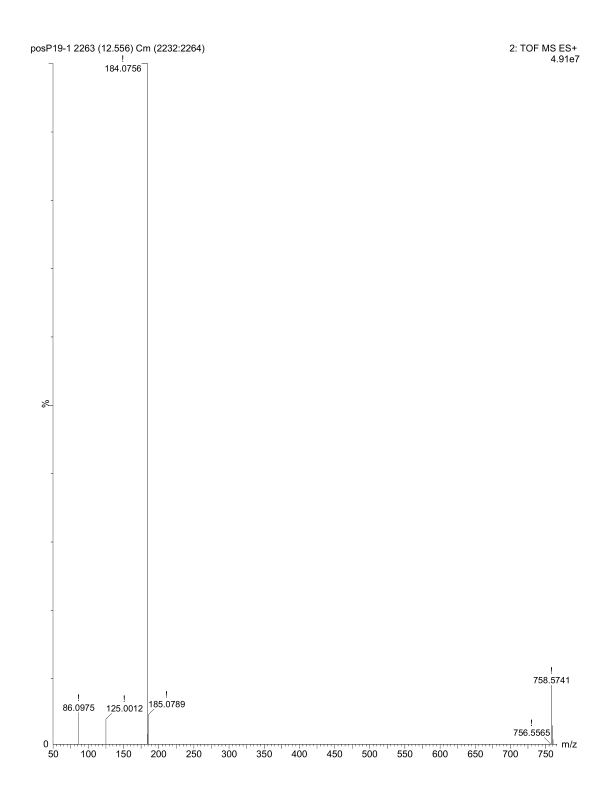 |

L-Tryptophan

| Mass  Bank | 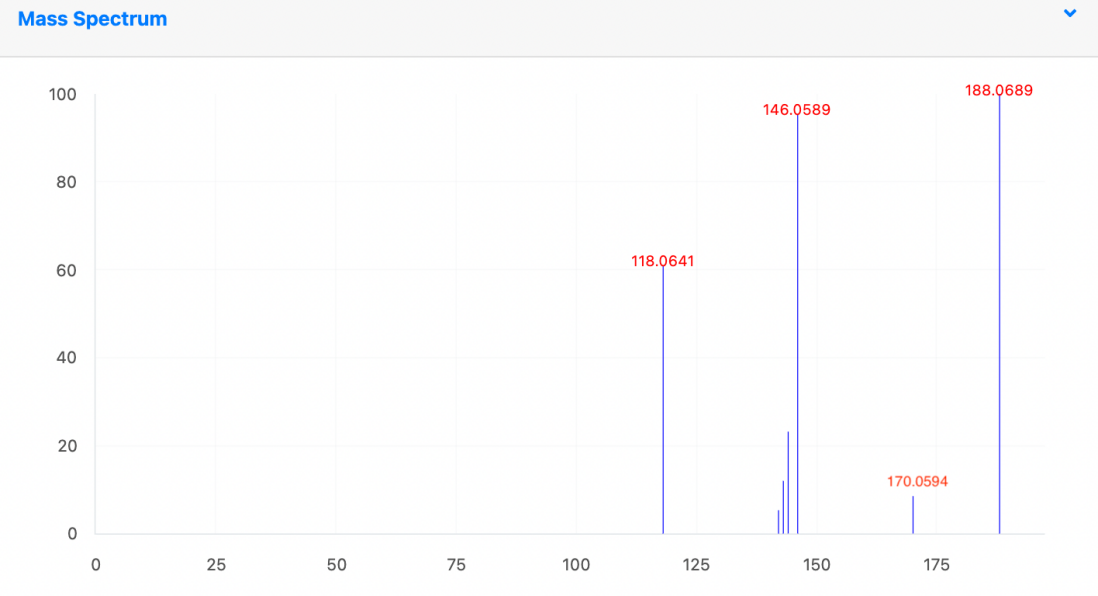 |
| --- | --- |
| Sample | 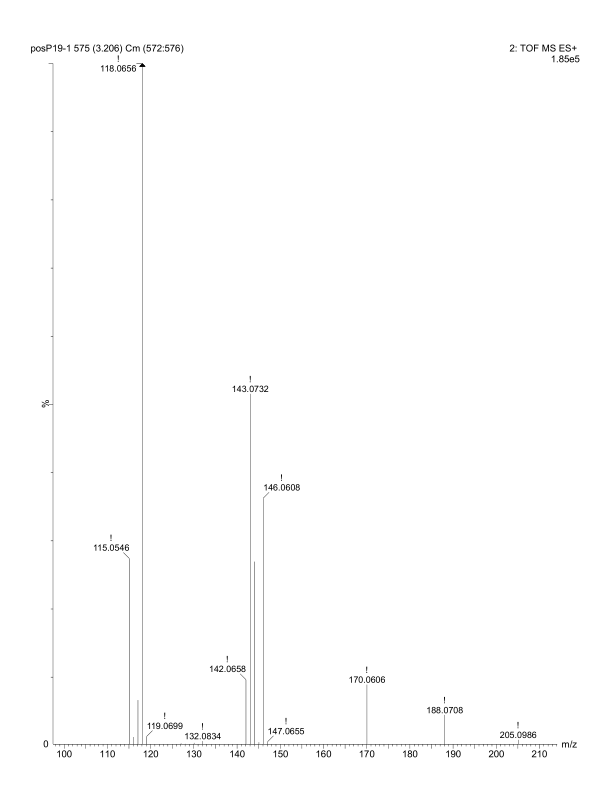 |

Xanthine

| HMDB | 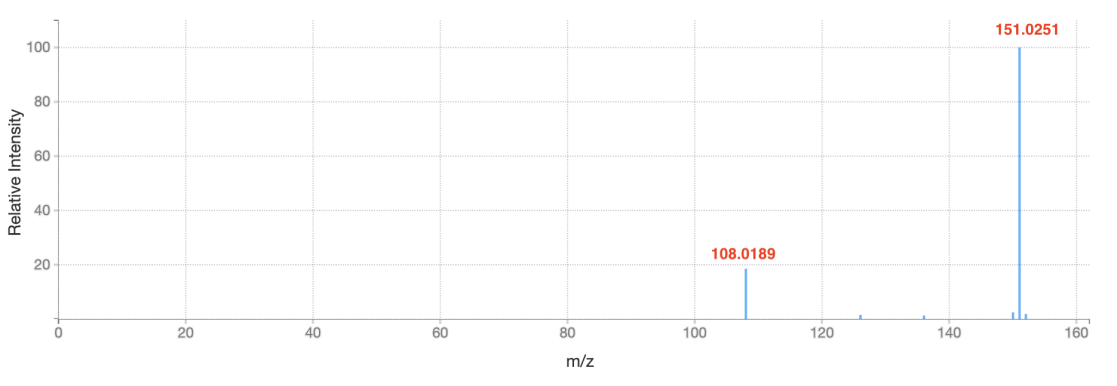 |
| --- | --- |
| Sample | 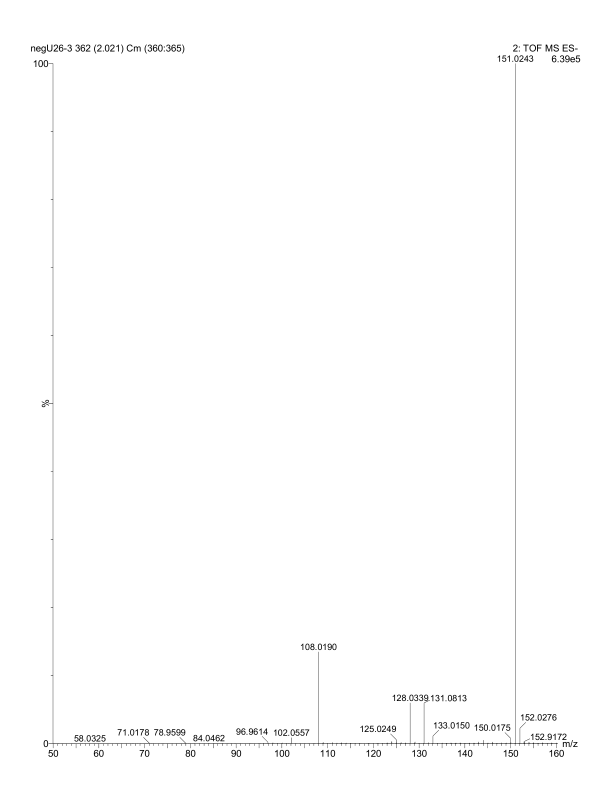 |

LPC(18:2(9Z,12Z))

| HMDB | 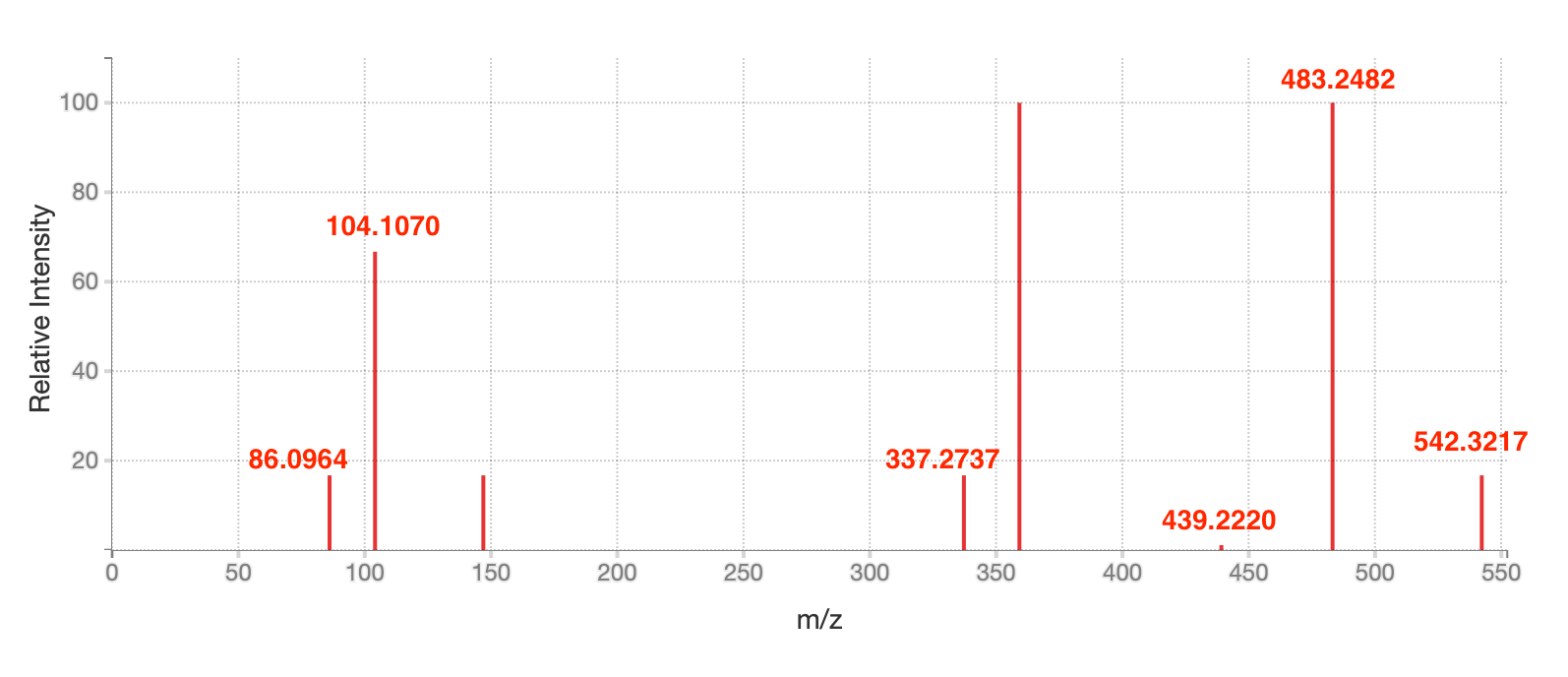 |
| --- | --- |
| Sample | 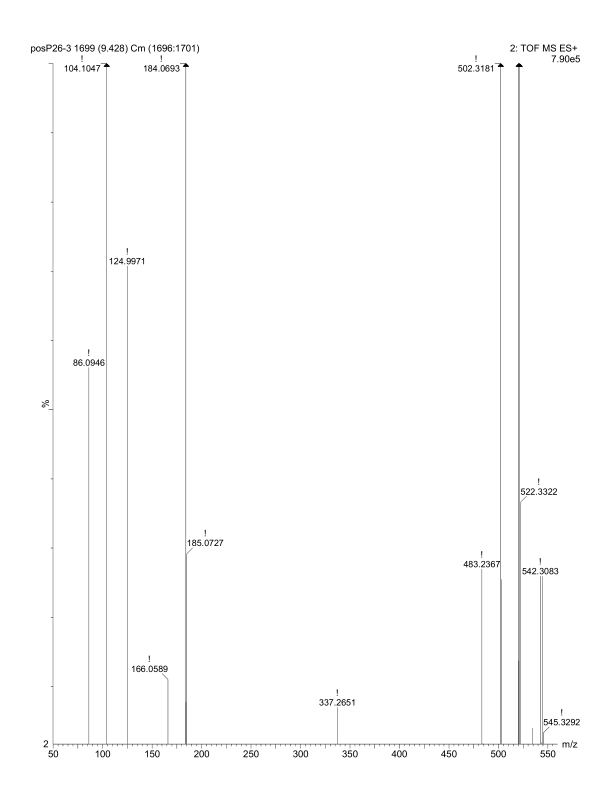 |

Cerulenin

| HMDB | 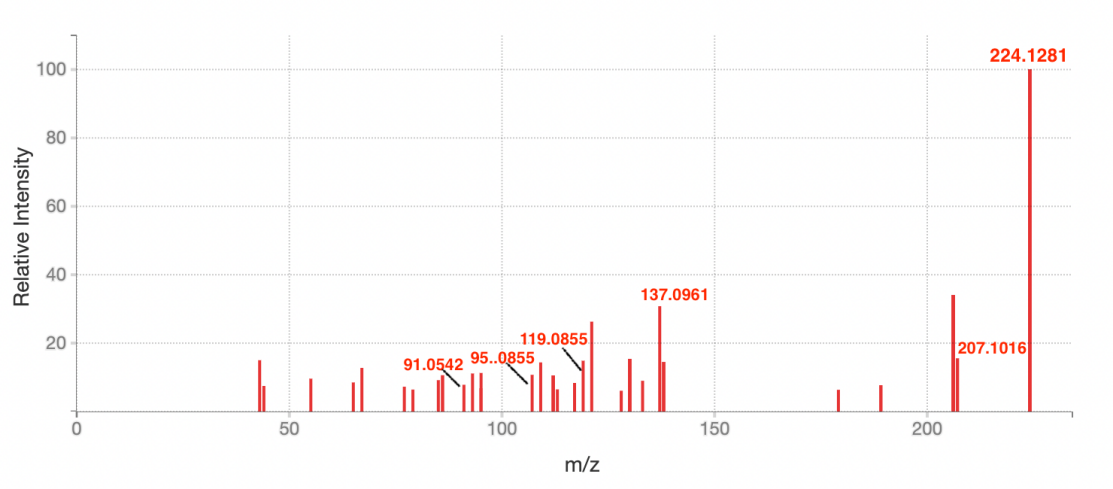 |
| --- | --- |
| Sample | 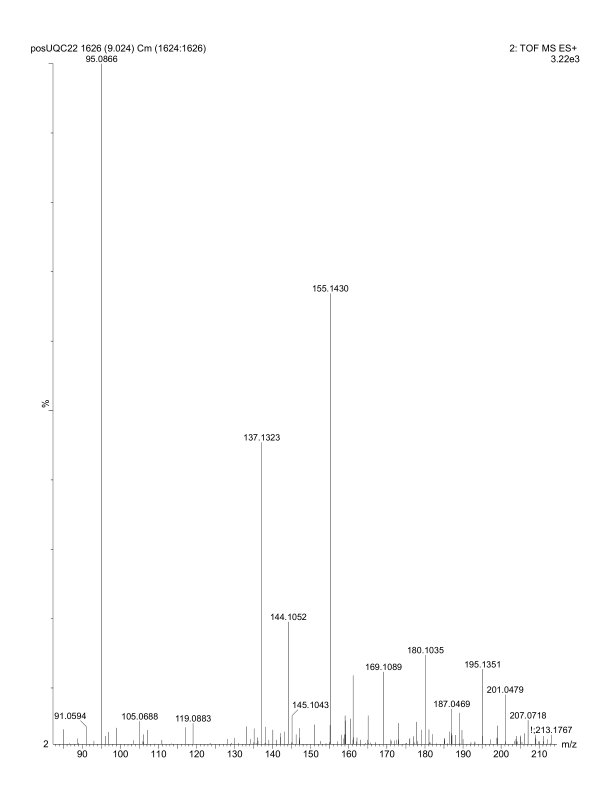 |

Pyroglutamic acid

| Mass  Bank | 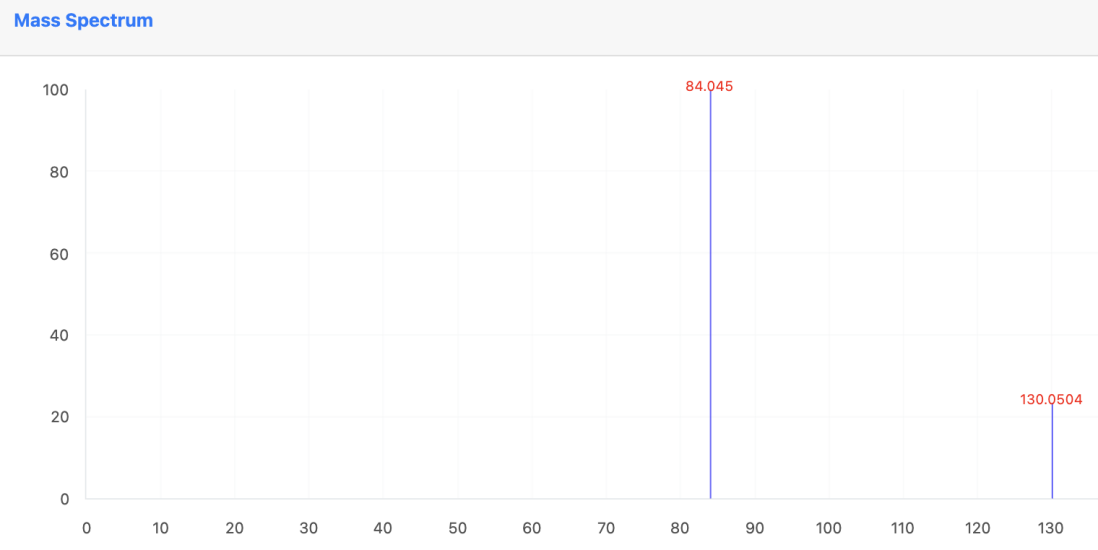 |
| --- | --- |
| Sample | 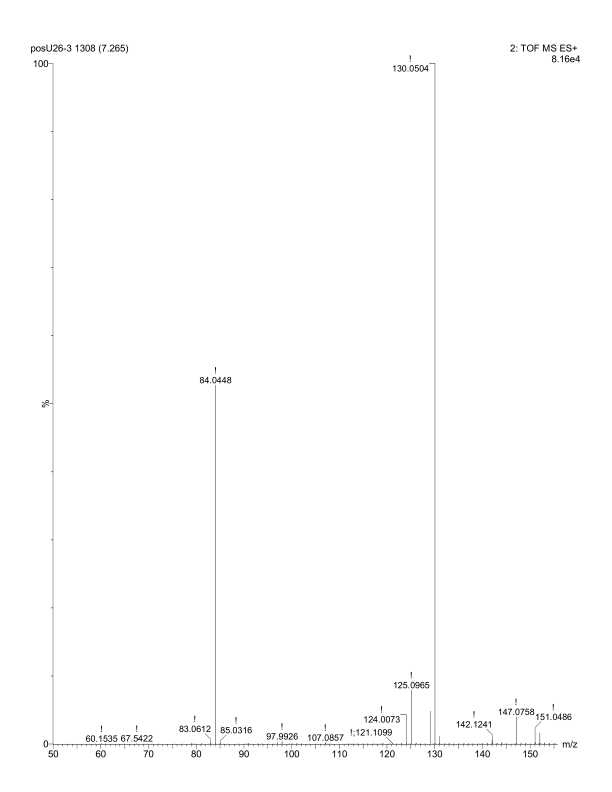 |
